# Automated Workflow for Preparation of cDNA for Cap Analysis of Gene Expression on a Single Molecule Sequencer

**DOI:** 10.1371/journal.pone.0030809

**Published:** 2012-01-30

**Authors:** Masayoshi Itoh, Miki Kojima, Sayaka Nagao-Sato, Eri Saijo, Timo Lassmann, Mutsumi Kanamori-Katayama, Ai Kaiho, Marina Lizio, Hideya Kawaji, Piero Carninci, Alistair R. R. Forrest, Yoshihide Hayashizaki

**Affiliations:** 1 Omics Science Center, RIKEN Yokohama Institute, Tsurumi-ku, Yokohama, Kanagawa, Japan; 2 K. K. Dnaform, Ono-cho, Tsurumi-ku, Yokohama, Kanagawa, Japan; Massachusetts General Hospital, United States of America

## Abstract

**Background:**

Cap analysis of gene expression (CAGE) is a 5′ sequence tag technology to globally determine transcriptional starting sites in the genome and their expression levels and has most recently been adapted to the HeliScope single molecule sequencer. Despite significant simplifications in the CAGE protocol, it has until now been a labour intensive protocol.

**Methodology:**

In this study we set out to adapt the protocol to a robotic workflow, which would increase throughput and reduce handling. The automated CAGE cDNA preparation system we present here can prepare 96 ‘HeliScope ready’ CAGE cDNA libraries in 8 days, as opposed to 6 weeks by a manual operator.We compare the results obtained using the same RNA in manual libraries and across multiple automation batches to assess reproducibility.

**Conclusions:**

We show that the sequencing was highly reproducible and comparable to manual libraries with an 8 fold increase in productivity. The automated CAGE cDNA preparation system can prepare 96 CAGE sequencing samples simultaneously. Finally we discuss how the system could be used for CAGE on Illumina/SOLiD platforms, RNA-seq and full-length cDNA generation.

## Introduction

The appearance of massively parallel next generation sequencing (NGS) technology has revolutionized the way we approach biology. Large scale sequencing projects are now more affordable and the data more broadly used in the research community. Massive scale projects aimed at understanding human variability and evolution such as the Thousand Genome Project (http://www.1000genome.org/) which is sequencing 1000 human individuals from various ethnic backgrounds [1], and the genome 10 K project [2] which will produce whole genome sequences for over 10000 vertebrate species would not be possible without this technology.

Similarly large scale efforts directed at understanding transcriptional regulation such as ENCODE and the Epigenome Roadmap have rapidly advanced with these technologies [3]. These projects use protocols such as RNA-seq [4]–[9], cap analysis of gene expression (CAGE) [10]–[12], chromatin immunoprecipitation [13]–[16] and bisulphite sequencing [17]–[19] to examine the transcribed regions of the genome, the association of transcription factors and arrangement and modifications of histones and DNA methylation to build an integrated overview of how the genome works. For instance, CAGE was used to produce extensive map of the mouse and human promoterome [20] and to prove that retrotransposon elements are specifically expressed in mammalian cells and tissues [21]. All of these technologies have been adapted from prior low throughput methods to generate libraries compatible for NGS systems.

**Figure 1:**
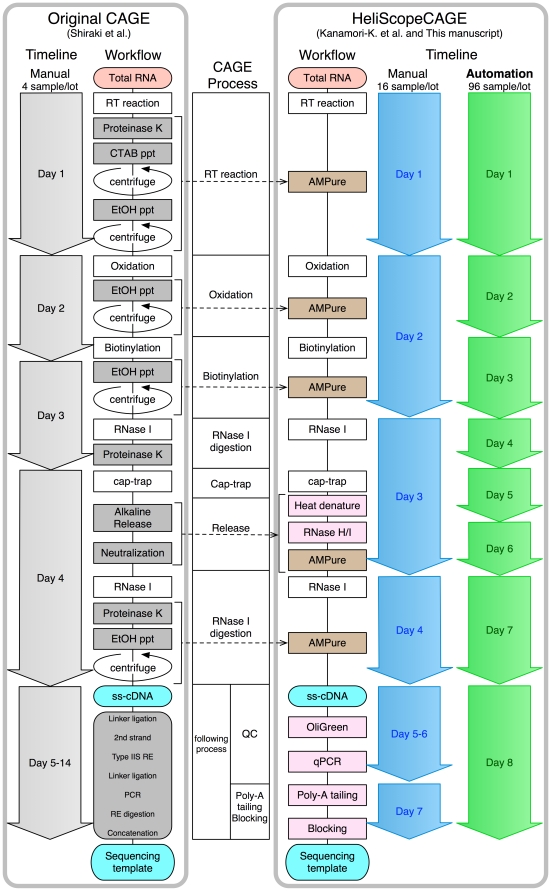
Simplification of the cap-trap process using SPRI and comparison between manual and automated processes. A: Flow chart comparing the original [34] and simplified cap-trapping protocols. All enzymatic inactivation and buffer exchanges and by alcohol precipitation were substituted with SPRI technology, AMPure purification. B: Comparison of time frame and throughput using the manual and automated workflows.

Despite the high throughput nature of the data generation, there is an increasing need to make these libraries in a high throughput and reproducible manner. Previously, we developed CAGE to comprehensively analyze transcription start sites (TSS). As CAGE both identifies TSS and measures expression levels, we have used this to measure activity of specific promoters and predict the transcription factors that regulate each [22]. We have adapted this protocol to all of the major 2nd generation sequencers (454, Illumina, and SOLiD) and most recently the HeliScope system [23], however until now CAGE has been a labor intensive manual protocol involving a large number of steps not easily amenable to automation [10](Figure 1A).

The original protocol consisted of reverse transcription (RT), oxidation, biotinylation, RNase I digestion, cap-trapping with streptavidin beads, cDNA release, 1st linker ligation, 2nd strand synthesis, type IIS restriction digestion, 2nd linker ligation, and PCR amplification with enzymatic deactivation and purification steps at each step [10](Figure 1A). The protocol also employed proteinase digestion, organic solvent extraction and alcohol precipitation for enzymatic inactivation and purification all of which are not easily amenable for high throughput library generation.

With our recent adaptation of CAGE to the HeliScope system we significantly simplified this protocol [23]. Basically we directly sequence the 3′ end of random primed CAP-trapped first strand cDNAs. Encouraged by recent reports on high throughput genomic template preparation systems [24], [25], we set out to adapt HeliScopeCAGE to an automated workflow. This was necessary as a single HeliScope machine requires 48 libraries every 10 days to be constantly running. Using the manual HeliScope CAGE protocol, even with a skilled operator, takes 4 days per 16 samples, and closer to 3 weeks to generate sufficient libraries for 1 HeliScope run of 48 samples, leading to down time, or increased operator costs. Moreover, manual preparation has more potential for human errors such as mis-orienting an 8-tube strip or swapping strips. Here, we report our automated workflow for 96-well plate format CAGE cDNA preparation. This system produces 96 sequencing-ready libraries per 8 days, generating enough libraries to keep 2 HeliScope machines running continuously and reduces operator costs while reducing potential human errors.

## Results

### Previous manual CAGE cDNA preparation workflow

In the original cap-trapping protocol for CAGE [10], there were 17 steps including a proteinase K digestion, phenol/chloroform extraction, and ethanol precipitations after every enzymatic reaction as shown in Figure 1A. These organic extractions and alcohol precipitations need centrifugation, which is not amenable to high throughput robotic automation. Therefore, in our recent manual HeliScope CAGE protocol [23] we replaced these purification steps with para-magnetic bead-based solid phase reversible immobilization (SPRI) technology [26], [27]. We chose AMPure RNAClean XP and AMPure XP (Beckman Coulter) for the purification of RNA/DNA hybrids or single strand (ss)-DNA, respectively. After the cap-trapping process, the yield of ss-cDNA was estimated using OliGreen (Life Technologies) and qPCR against ribosomal RNA and Beta actin were used to estimate the ribosomal content of each library. After this poly-A tailing/blocking is carried out prior to HeliScope sequencing [23]. Following this protocol using an 8 channel multi-pipette a technician can prepare 16 samples every 8 days. Therefore, 3 weeks (or 3 technicians for one week) are needed to produce enough libraries (48 samples) for one run of HeliScope.

### Layout of the automated library system

Given the timeframe detailed above and the capacity of next generation sequencers we sought to adapt the protocol to a 96 well automated workflow. Out first step was to configure a robotic solution to carry out all of the necessary liquid handling, incubations and purifications. Our system is based on a TECAN Freedom Evo 150 platform with an 8-channel liquid handling arm (LiHa), and a robotic manipulator arm (RoMa) (Männedolf, Switzerland) as described in Figure S1. The stage was configured with chilling, heating and room temperature blocks and reagent reservoirs (4°C, 37°C, RT) for incubations and dispensing of reagents. A thermal cycler with auto-hot bonnet (Bio-Rad) and a fluorescence plate reader (TECAN) was also integrated into the system. The layout and all scripts are described in Figure S1 and Text S1. This system can perform the full CAGE cDNA preparation process including RT reaction, oxidation of diol groups, biotinylation, cap-trapping, release, and quantification of produced ss-cDNA.

Using this system 1 technician can prepare 96 libraries in 8 days which is enough for two HeliScope runs and in practice two systems can be used in parallel by the same technician increasing the throughput to 192 per 8 days.

### Modifications of the protocol to improve handling and yield on the automated system

A simple transfer of the manual protocol to the automated system was not enough to achieve our goal of a fully automated system as several technical issues lead to reduced library yields. Starting from our manual protocol, we made several adjustments to the SPRI steps that were critical to reduce foaming in the mixing steps and bead loss during washing steps. To avoid bead loss, we added ethanol during the bead purification stage. After fixing beads by magnetic rings, the machine aspirates the supernatant to just above upper edge of the bead rings, and then 100% ethanol was added at the final concentration of 70% to tighten the beads ring. We also added isopropanol to 25% volume just before adding the AMPure slurry to avoid foaming by pipetting in the oxidation and biotinylation steps (these steps are more susceptible to foaming). Further details of the modifications are described in Material and Methods and Table S1. In principal adjustment of the alcohol concentration may result in shorter cDNA products being included in the final libraries however we confirmed neither adjusting the ethanol or isopropanol concentrations had any effect on the size selection of AMPure by checking purification of a range of molecular weight markers (25 bp ladder) under the above conditions. We also analyzed the fold change distributions between manual and automated CAGE sequencing for genes of different lengths in Figure S3 and found no significant difference between them. In addition as we demonstrate below, manual and automated library protocols on the same RNAs are highly correlated indicating no systematic difference is introduced. Note these modifications should not affect removal of enzymes or efficiency of buffer exchange (the key reason for using this technology). Further details of these and other minor adjustments are listed in Table S1.

### CAGE cDNA yield and quality

For the evaluation of our automated system, we prepared 2 batches of 96 libraries and included 18 replicate wells of the same RNA (THP-1 [28] total RNA) distributed across the columns and rows of the plate to assess well-to-well reproducibility (Figure S2). The remaining wells were filled with a diverse set of RNA samples collected for the FANTOM5 (Functional Annotation of Mammalian Genome 5) project to assess general variability in a real production scenario.

The yield of cDNA from 5 µg of total RNA for each library was measured using OliGreen. For manual libraries the yield for THP-1 was 15.2±2.3 ng while the automated THP-1 libraries ranged from 8.9 to 22.8 ng with an average yield of 12.5±3.5 ng (Table 1 and Table S2). These values were slightly lower but produced more than the 5 ng of material required for loading on the HeliScope. For the 78 FANTOM5 production samples, the yield ranged from 3.9 to 13.9 ng with an average of 8.9 ng.

**Table 1:** Evaluation of replicate THP-1 CAGE libraries.

Preparation	Manual	Automation1	Automation2
Yield (ng)	15.2±2.3	12.5±3.5	15.1±3.0
ACTB Ct	14.5±0.4	14.6±0.2	14.7±0.2
18S rRNA Ct	15.0±0.2	13.7±0.4	14.0±0.6
delta Ct	−0.6±0.4	0.9±0.3	0.7±0.3
Promoter ratio(%)	68.8±8.3	53.4±3.5	63.4±1.8
rRNA ratio (%)	1.5±0.2	4.1±0.5	2.1±0.4

The quality control values for all replicate THP-1 CAGE libraries and sequencing/mapping evaluation are listed. All values are averages with standard deviations. The Ct values of ACTB and 18S rRNA are representatives of non-capped and capped transcripts. The primers were designed at near 5′ end of each transcript. The promoter ratio and rRNA ratio are the rates of reads mapped at 5′ end per total filtered reads. The detail information is shown in Table S2.

To check the quality of the cap-trapping, we also carried out qPCR against the both 5′ end of the beta actin gene and the 18S ribosomal RNA. If cap-trapping is working efficiently, the beta actin 5′ end should be captured at a higher rate than the 5′ end of 18S rRNA that is not capped. Delta Ct was calculated between the beta actin and 18S rRNA for the manual and automated THP-1 libraries. The quality check by qPCR for the replicates showed quite similar values among samples; average actin beta 5′ end qPCR Ct was 14.6±0.2, and delta Ct to rRNA was 0.9±0.3. If we carry out this same quality check on standard cDNA without cap-trapping we see a delta Ct to rRNA of 5.7±0.2 indicating enrichment of capped over uncapped transcripts..

Based upon the OliGreen results ∼10 ng of 1st strand cDNA libraries were then poly-A tailed and blocked [29], [30]. After tailing, half of the poly-A tailed/blocked samples were then loaded onto a HeliScope flow cell channel manually using the HeliScope Sample Loader [30].

### Quality of CAGE libraries assessed by sequencing

**Figure 2:**
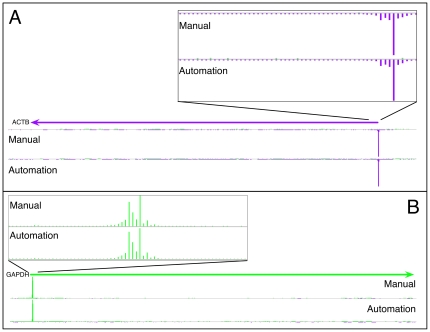
Genomic view of selected genes for comparison between manual and automation methods. CAGE mapped read counts were displayed as linear scale histogram on ACTB (A) and GAPDH (B) genes. The transcription initiation regions are magnified to demonstrate the tag density distribution is consistent between manual and automated libraries. Green and purple indicate plus and minus strands, respectively.

To further evaluate the libraries, we carried out HeliScope sequencing of the above libraries plus a second automation batch. The sequencing output is shown in Table 1 and Table S2. After filtering for poor quality reads approximately 20 million reads were obtained for each library. Filtered reads were then aligned to human genome (Hg19) and high quality alignments kept for further analysis (see methods for description of Alignment and filtering). This yielded approximately 6 million high quality alignments. The ratio of promoter-associated tags was then estimated by counting the number of aligned tags within 500 bases of the 5′ end of mapped Refseq transcripts. For the manual libraries prepared using THP-1, 68.8±8.3% of the aligned tags were promoter associated, while for the automated libraries it was lower with a rate of 53.4±3.5% and 63.4±0.6% on average for both 2 batches. Despite this we obtained more than 2 million promoter mapped reads from these libraries. Example genome browser images are shown for the GAPDH and ACTB loci. There is good agreement of CAGE tag distribution in the transcription initiation regions for both of these genes using the automated and manual library preparations (Figure 2). Applying the protocol to different RNA sources we observe similar results, e.g. the promoter mapping rates of replicate HeLa [31] samples from automated preparation batch 1 and 2 were 59.0% and 66.5% while mouse whole embryo (E17.5) RNA yielded 51.8–53.1% promoter associated tags (Table S2.). We have observed that promoter ratios in production libraries typically vary between 50–75% depending on the source of the RNA. Well studied RNA sources (e.g. HeLa, fibroblast) containing a large fraction of transcripts well represented in Refseq typically have higher promoter hit rates while those of rarer cell types have lower hit rates (i.e. Our estimated promoter ratio is a function of which transcripts have been recorded in Refseq). The slightly lower promoter hit rates we report in mouse whole embryo libraries when compared to those of THP-1 or HeLa libraries are likely to be a function of the annotation depth and quality in each species. We recommend this metric is only comparable for samples from the same species.

### Reproducibility across multiple wells and batches

**Figure 3:**
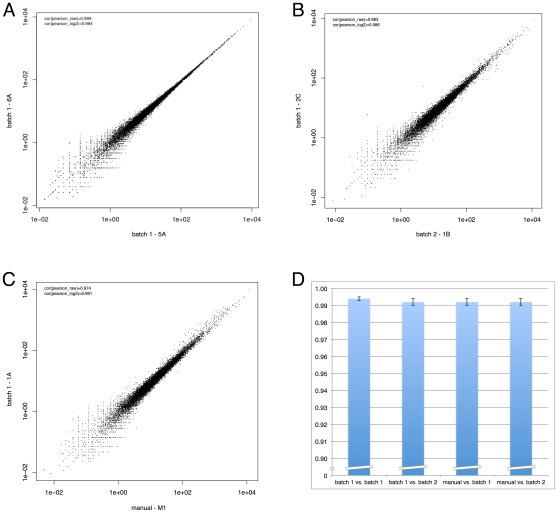
Representative scatter plots demonstrating reproducibility between manual and automated workflows and reproducibility within batches and between batches using automation. A–C: shows scatterplots of TPM normalized gene expression for A, two technical replicates in the same batch; B: between technical replicates from different batches and; C: between manually and automatically prepared technical replicates. Finally D: shows the average correlation coefficient when comparing multiple replicates from automation and manual libraries. Error bars indicate the standard deviation.

To further assess our libraries we measured expression of known genes by counting CAGE tags aligning to the genome within 500 bases of a Refseq 5′ end. These expression profiles were then compared across replicates and batches. The expression profiles for the THP-1 replicates within automation plate1 were highly correlated with a range from 0.990 to 0.996 and with the average of 0.994±0.001 of Pearson coefficient of correlation among these replicates (See Figure 3A, 3D and Table S3).

Similarly technical replicates from different automation batches were highly correlated with coefficients of correlation between libraries from different batches ranging from 0.986 to 0.994 with an average of 0.992±0.002 (Figure 3B and 3D). Finally we also compared the expression from automated libraries with manual libraries. The average coefficients of correlation between manual and each automation batch were both 0.992±0.002 (the ranges were 0.987–0.994 for manual vs. batch 1; 0.990–0.994 for manual vs. batch 2) (Figure 3C and 3D). These data indicate that the CAGE cDNA automatic preparation system can produce highly reproducible CAGE cDNA samples comparable to manually prepared libraries but in a high throughput manner effectively saving 5 operator weeks per 96 samples (Note: the full set of correlations is shown in Table S3).

## Discussion

Here we have reported the development of an automated high throughput 96-well CAGE cDNA preparation system based on the TECAN Freedom Evo 150 system coupled with a thermal cycler with automatic hot bonnet, and fluorescence plate reader. Starting from a 96 well plate of aliquoted RNA the system can go through RT, cap-trapping and the poly-A tailing/blocking necessary for CAGE on HeliScope. The CAGE cDNA prepared by this system showed high reproducibility both within and between batches and is comparable with manually prepared libraries. This system can prepare 96 sequencing-ready cDNA samples in 8 days; equivalent to 2 runs on HeliScope. The automation has been critical in increasing throughput without increased cost (and variability) of additional manual operators. In addition the system does not require full time supervision; therefore it is possible to have one technician operate two of more systems in parallel.

We currently have 2 systems in place allowing us to produce 192 CAGE libraries every 8 days. With this capacity it now becomes possible to carry out large scale projects such as FANTOM5 with greater reproducibility and lower staffing costs. In the FANTOM5 project we are using this system to generate thousands of CAGE libraries to survey transcription initiation across a broad collection of RNAs. Using this data we can predict transcriptional regulatory programs based upon both co-expression of transcription factors and target genes and the presence of transcription factor binding sites in the proximal promoter regions adjacent to the CAGE peaks [22]. Similarly we have reported a pilot experiment using siRNA knockdown and CAGE profiling to build perturbation networks [32]. Without automation such an approach remains at a pilot stage. For large scale network elucidation using deep sequencing approaches (CAGE, RNA-seq, ChIP-seq), the scale of production and price per experiment matters.

Although we have focused in this paper on generating CAGE libraries compatible with the HeliScope system, with some modifications to the workflow or reagents, the same system can also be used for CAGE on the Illumina or SOLiD platforms, RNA-seq and cap trapped full-length cDNA generation. For example to generate an Illumina or SOLiD compatible CAGE library on this system, the cap trapped first strand cDNA generated on the current system could be used. This would then require a 5′ linker ligation step (incorporating the Illumina/SOLiD 5′ primer and a type II restriction enzyme site), a 2nd strand synthesis step, tag cleavage step and a 3′ linker ligation step and PCR step [11]. These additional steps could all be carried out on this system. Obviously such a protocol is more complex than the protocol for HeliScope, but this would allow more users access to the CAGE protocol.

Finally adaptation of the system for strand specific RNA-seq libraries is relatively straight forward. An RNA fragmentation step, linker ligation step and PCR step could all be carried out on this system and would be compatible with indexed linker reagents available from Illumina (TruSeq Small RNA kit) and Life Technologies (SOLiD Total RNA-Seq Kit). Finally by modifying the priming strategy [33] full-length cap trapped cDNAs could be generated on this system. The high throughput generation of full length cDNAs and their subsequent sequencing on 3rd generation sequencing single molecule platforms in development (e.g. Nanopore and PacBio) could be important in elucidating splicing complexity beyond the era of short read RNA-seq.

## Materials and Methods

### RNA for the CAGE cDNA preparation

The RNA was prepared by Qiagen RNeasy Micro kit (Hilden, Germany). Alternatives such as Trizol and Nucleospin have also been tested. The RNA integrity and quality should be evaluated. RNA with a RIN value >7 Agilent BioAnalzer (Santa Clara, CA) and absorbance ratio of 260/230 nm (A_260_/A_230_) >1.8 should be used to ensure data quality. Starting amount of RNA should be 4.5∼5 µg.

### Reagents for Automatic workflow

All reagents for CAGE cDNA preparation automatic workflow are same as manual workflow described in Text S2 with ethanol and isopropanol. All reagents must be RNase-free.

### 16 sample manual protocol for CAGE on HeliScope

The manual protocol (Text S2) uses an 8-channel multi-pipette. The whole process consists of 9 major steps involving 1st strand synthesis by RT, oxidation with sodium periodate, biotinylation with biotin hydrazide, RNase I treatment to remove 3′ end biotin, cap-trapping with magnetic streptavidin beads, RNase H and RNase I digestion, quantification by fluorescence assay using OliGreen fluorescence assay kit (Life Technologies), mass normalization, and poly-A tailing/blocking for HeliScope sequencing. Before quantification, DNA/RNA molecules were purified by RNAClean or AMPure XP. The purified ssDNA was then concentrated using a SpeedVac, and then dissolved in 12 µl of water. 1 µl of sample was then used for quantification by Quant-iT OliGreen ssDNA Reagent (Life Technologies) and qualification by qPCR as described in Quality Check of CAGE cDNA, respectively.

### Robotic optimized CAGE protocol

The HeliScope manual protocol described in Text S2 was modified to optimize for robotizing. In the AMPure purification step, we needed to modify the protocol to avoid aspirating magnetic beads carrying the cDNA while removing the supernatant. This was primarily because of loose aggregation of the beads. To avoid this we carried out the aspiration in two steps. First we loosely aggregate the beads using a magnet ring array and removed supernatant above the ring (leaving ca. 60 µl). Then, 140 µl of 100% ethanol was added to tighten beads aggregation, and the remaining supernatant was then removed.

Similarly at the nucleic acid binding step, pipetting and dispensing can cause bubbles to the slurry, causing unevenness of aspiration volume and/or machine halt problems because of liquid surface sensing failure. To solve this problem, isopropanol was added to 25% to avoid bubbling. Also, the mixing by pipetting was performed by adjusting the tip height position by sensing the liquid surface. At the suspension step of elution in AMPure purification or washing and elution step in streptavidin beads selection, good re-suspension is important to wash and recover the bound nucleic acid. However, normal pipetting at the center position of the well had problems re-suspending tightly aggregated pellets. Therefore, we shifted the pipet tip position from center to slightly near the wall. This generated a disarranged liquid flow which efficiently dispersed the aggregated beads. We also use ‘dolphin tubes’ (Sorenson Bioscience Inc.) whose bottom is more slender than the usual 2 ml tube for the AMPure slurry reservoir to make the suspension more uniform. All modifications for robotizing are shown in Table S1. At the poly-A tailing/blocking step [29], we decided to use 10 ng or a half if the yield was less than 20 ng. Therefore, the system picked 10 ng or half aliquots, and then subjected to poly-A/blocking reaction. After the reaction, the half of poly-A tailed/blocked samples were used for the loading on HeliScope flow cell channel manually using HeliScope Sample Loader. We loaded the samples following Helicos Low-Volume Sample Loading Protocol, LB-017.

### Quality Check of CAGE cDNA

Cap-trapping efficiency was checked by examining the yields and Ct values of qPCR using the primer sets against a capped transcript; ACTB 5′ end (Human ACTB Fw: 5′-GGCATGGGTCAGAAGGATT-3′; Human ACTB Rv: 5′-AGGTGTGGTGCCAGATTTTC-3′), (Mouse ACTB Fw: 5′- TATCGCTGCGCTGGTCGTCG-3′; Mouse ACTB Rv: 5′- TAGGGCGGCCCACGATGGAG-3′) and uncapped transcript 18S rRNA 5′ end (Human 18S rRNA Fw: 5′-CTGGTTGATCCTGCCAGTAG-3′; Human 18S rRNA Rv: 5′-TCTAGAGTCACCAAAGCCGC-3′), (Mouse 18S rRNA Fw: 5′- GCCATGCATGTCTAAGTACGCACG-3′; Mouse 18S rRNA Rv: 5′- TCAGCGCCCGTCGGCATGTA-3′).

The amplification detection was done by using SYBR Green on ABI PRISM 7900 HT (Life Technologies). The thermal cycle program was 95°C for 15 min, then 40 cycle of 94°C for 15 sec, 60°C for 30 sec, and 72°C for 30 sec, then 95°C for 15 sec, 60°C for 15 sec, and finally 95°C for 15 sec. The data analysis condition was thresh hold: 0.2 and Manual baseline: 3–13.

### HeliScope sequencing

Sequencing on the HeliScope Genetic Analysis System (Helicos Biosciences) was performed following manufacturer's manual. Depending on the yield we either used 5 ng or a quarter of the library if the yield was less than 20 ng for sequencing. Aliquots were taken and then pol-A tailed and blocked [29]. After the reaction, half of the poly-A tailed/blocked samples were loaded on the HeliScope flow cell channel manually using the HeliScope Sample Loader. We loaded the samples following Helicos Low-Volume Sample Loading Protocol, LB-017. The sequencing data from this study have been submitted to the DDBJ Read Archive (http://trace.ddbj.nig.ac.jp/dra/index_e.shtml) under accession no. DRA000496.

### Alignment and filtering

Raw Helicos reads containing base-order addition artifacts and other low quality reads were removed using the filterSMS program supplied by Helicos. In addition reads shorter than 20-nt and longer than 70-nt were removed from further analysis. All filtered reads were then mapped to the human genome (hg19) using Delve (T. Lassmann in preparation). In brief, Delve uses a pair hidden Markov model to iteratively map reads to the genome and estimate position dependent error probabilities. After all error probabilities are estimated, individual reads are placed to a single position on the genome where the alignment has the highest probability to be true according to the pHMM model. Phred scaled mapping qualities, reflecting the likelihood of the alignment at a given genome position, are also reported. Reads mapping with a quality of less than 10 (<90% chance of true) were discarded.

## Supporting Information

Figure S1 **The layout of TECAN Freedom Evo 150 system for HeliScope CAGE automated preparation.** A: The layout of TECAN Freedom Evo 150 system for HeliScope CAGE automated preparation. All stages, reservoirs, hotels and equipment are shown as position numbers listed in C; B: The outward appearance of the system; C: The list for every stages, reservoirs, hotels and equipment. The position numbers are consistent with the layout A.
(TIFF)

Figure S2 **Evaluation sample layout.** The 96-well PCR plate was used for the preparation. Blue wells were for the replicated samples of THP-1 total RNA. Red was for HeLa total RNA. After the preparation, the samples were split into 2 groups, column 1 to 6 and 7 to 12, for 2 runs on HeliScope. All samples were loaded on flow cells by following the manufacturer's instruction.
(TIFF)

Figure S3 **Boxplot showing fold change distributions for manual CAGE vs automated CAGE measurements for Refseq genes of varying lengths.** The fold change for manual/automated were calculated for CAGE signal within +/−500 bp of all Refseq genes. Plots for Refseq genes of <250, 250–1500 and >1500 bases in length are plotted. No significant difference based on size was observed. Box shows the interquartile range.
(TIF)

Table S1 **Additional improvements for automatic CAGE cDNA preparation.** All additional improvements of every step in automated HeliScope CAGE cDNA preparation process are listed. All steps and script line numbers are indicated in left columns. The script line numbers are consistent with the Table S3.
(XLS)

Table S2 **Whole evaluation samples output and QC values.** All quality control values, sequencing, and mapping results are listed. The evaluation replicates were total RNA derived from THP-1 cell line. The other samples derived from HeLa and mouse embryo 17.5 days are also listed for the reference. Raw count is the total read numbers of sequencing. Filtered count is after the filtering described in Methods. Ribosomal mapped is the read numbers mapped on ribosomal DNA, which are involved in the filtered out reads. rRNA rates are calculated from assembled rRNA counts that are mapped on mature ribosomal RNA per filtered counts. The other categories indicate the mapped positions and its rates.
(XLS)

Table S3 **Correlation coefficients among replicas and batches.** All correlation coefficients among all replicates of THP-1 total RNA prepared by manual and automated process are listed. The top down order of replicates on y-axis is consistent with left-right order of x-axis. The cell color is scale of the values between yellow and green. The maximum and minimal values among them are 0.997 and 0.986, respectively. The average of all replicates is 0.993±0.002.
(XLS)

Text S1 **Robotic optimized CAGE cDNA preparation script for TECAN Freedom Evo 150 system.** The script of whole HeliScope CAGE preparation process for TECAN Freedom Evo 150 is listed. The format is TECAN software output.
(DOC)

Text S2 **Simplified CAGE cDNA preparation protocol for HeliScope sequencing by manual.** The protocol for the manual preparation of HeliScope CAGE cDNA using 8-channel multi-pipette is described. Double exclamation mark starting sentences in red are attention in this protocol. Sharp mark sentences in green are the hints. Asterisk mark sentences in purple are safe rest points to suspend the protocol.
(DOC)
