# Supplementary figures and images for: Automated Workflow for Preparation of cDNA for Cap Analysis of Gene Expression on a Single Molecule Sequencer
Source: PLoS One. 2012 Jan 30;7(1):e30809. doi: 10.1371/journal.pone.0030809 (PMC3268765; doi:10.1371/journal.pone.0030809)

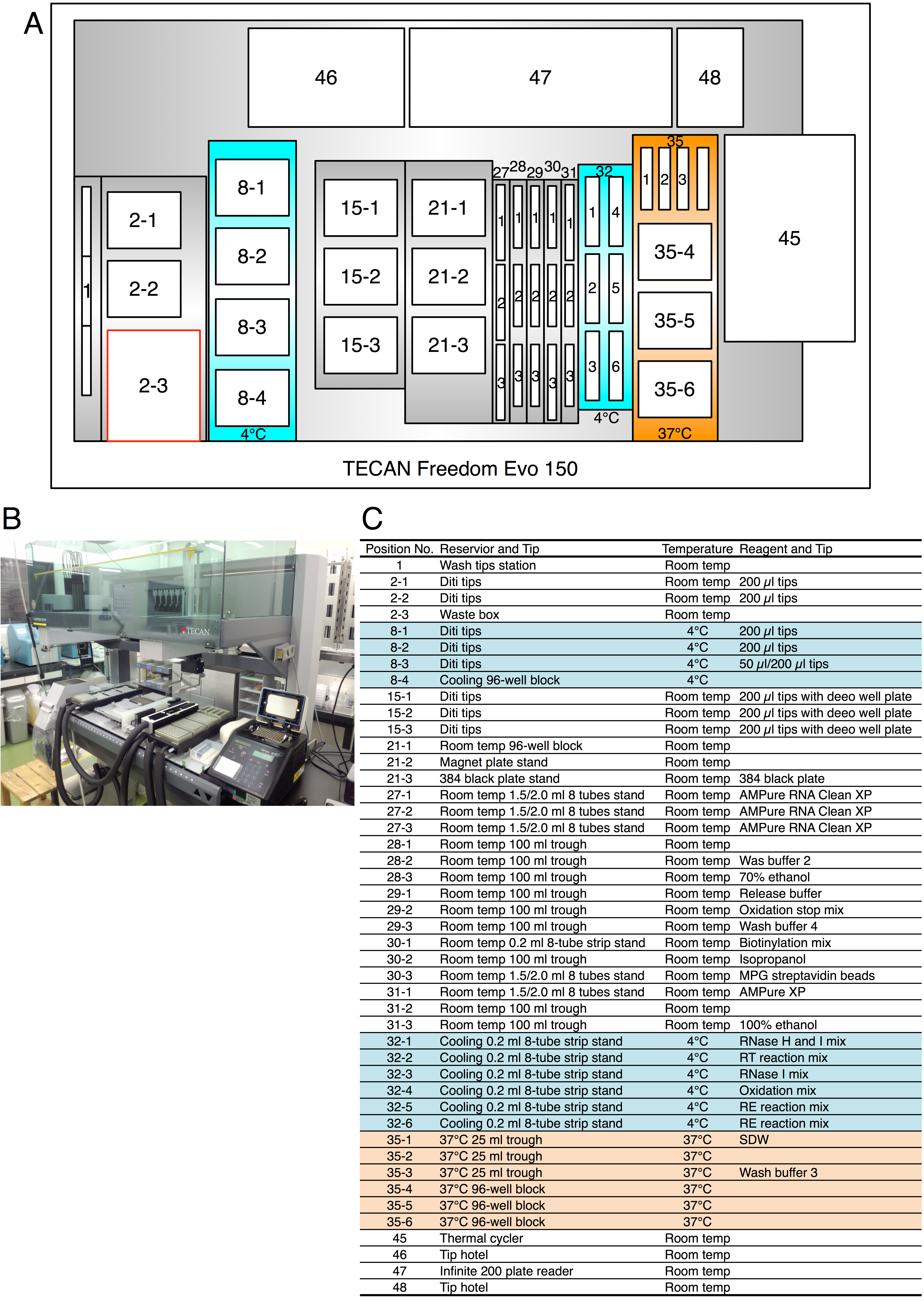

Supplement: Figure S1 — The layout of TECAN Freedom Evo 150 system for HeliScope CAGE automated preparation. A: The layout of TECAN Freedom Evo 150 system for HeliScope CAGE automated preparation. All stages, reservoirs, hotels and equipment are shown as position numbers listed in C; B: The outward appearance of the system; C: The list for every stages, reservoirs, hotels and equipment. The position numbers are consistent with the layout A. (TIFF) [file pone.0030809.s001.tif]

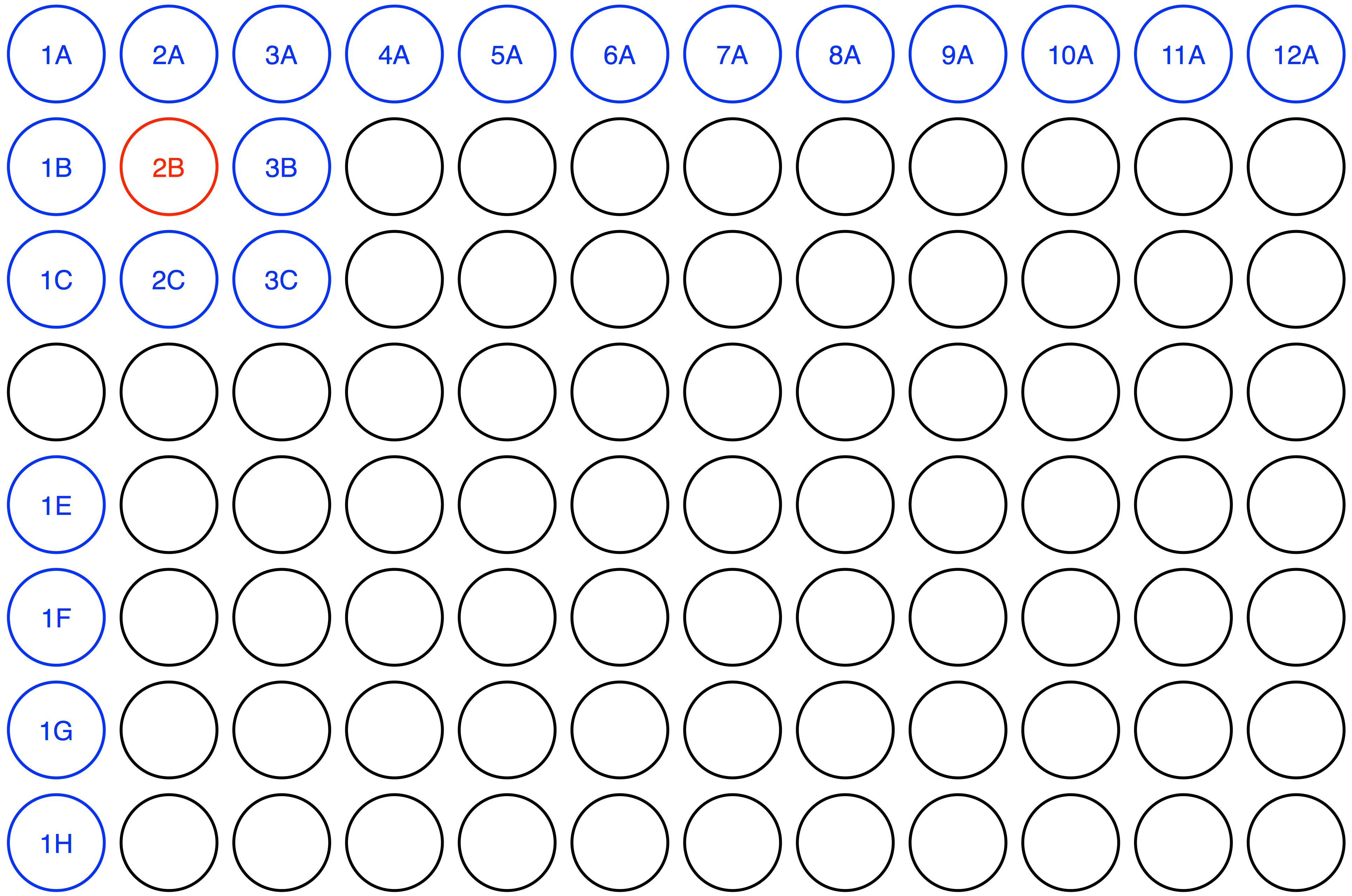

Supplement: Figure S2 — Evaluation sample layout. The 96-well PCR plate was used for the preparation. Blue wells were for the replicated samples of THP-1 total RNA. Red was for HeLa total RNA. After the preparation, the samples were split into 2 groups, column 1 to 6 and 7 to 12, for 2 runs on HeliScope. All samples were loaded on flow cells by following the manufacturer's instruction. (TIFF) [file pone.0030809.s002.tif]

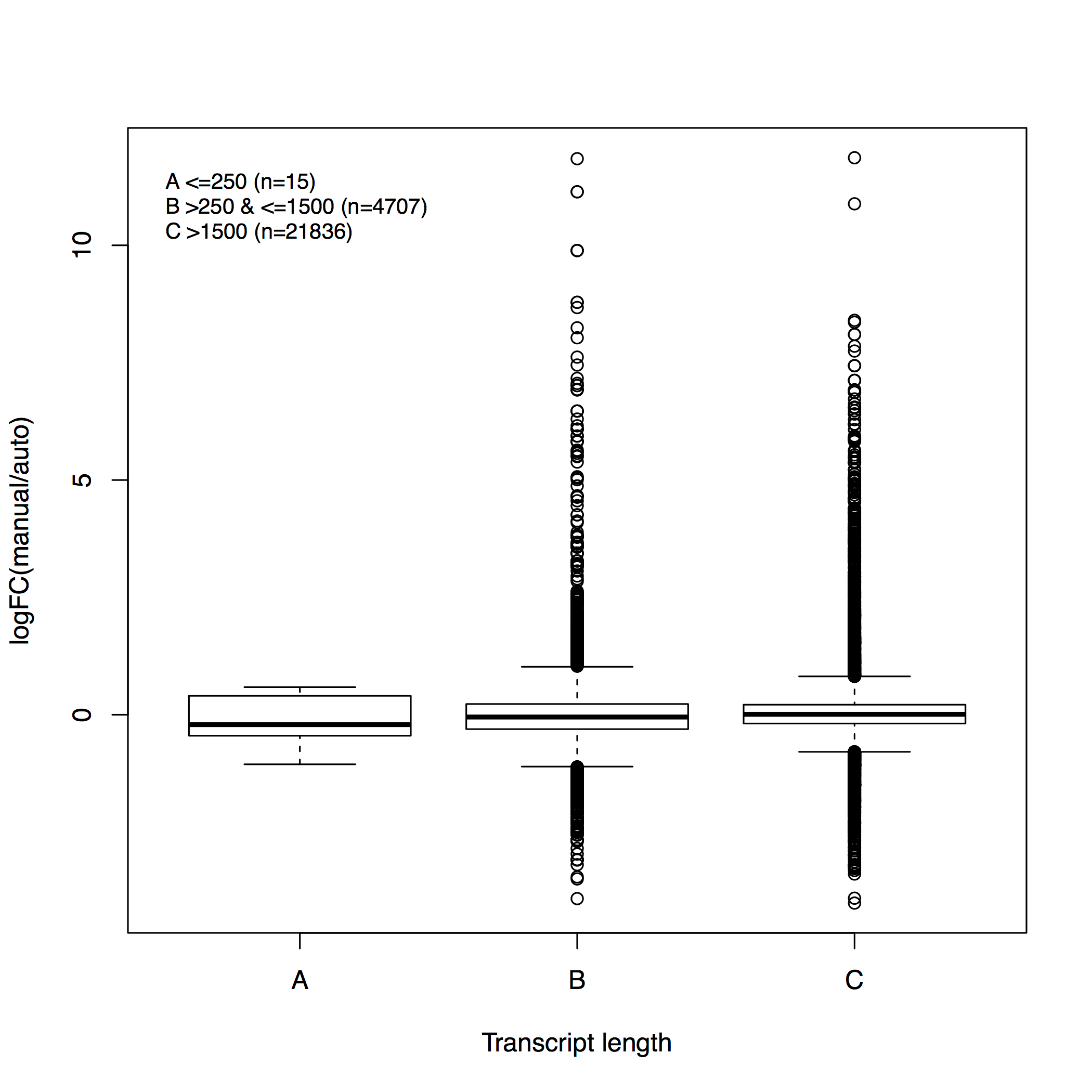

Supplement: Figure S3 — Boxplot showing fold change distributions for manual CAGE vs automated CAGE measurements for Refseq genes of varying lengths. The fold change for manual/automated were calculated for CAGE signal within +/−500 bp of all Refseq genes. Plots for Refseq genes of <250, 250–1500 and >1500 bases in length are plotted. No significant difference based on size was observed. Box shows the interquartile range. (TIF) [file pone.0030809.s003.tif]
