# Supplementary material for: Automated Workflow for Preparation of cDNA for Cap Analysis of Gene Expression on a Single Molecule Sequencer
Source: PLoS One. 2012 Jan 30;7(1):e30809. doi: 10.1371/journal.pone.0030809 (PMC3268765; doi:10.1371/journal.pone.0030809)
Supplement: Text S1 — Robotic optimized CAGE cDNA preparation script for TECAN Freedom Evo 150 system. The script of whole HeliScope CAGE preparation process for TECAN Freedom Evo 150 is listed. The format is TECAN software output. (DOC) [file pone.0030809.s007.doc]

**1. 1st_STRAND_SYNTHESIS_and_PURIFICATION**

**---START---**

C0F84065

20110617_164414 Admin

Administrator

--{ RES }--

V;200

--{ CFG }--

999;219;32;

14;-1;30;8;-1;-1;-1;-1;-1;234;-1;-1;-1;-1;-1;-1;96;-1;-1;-1;-1;-1;34;-1;-1;-1;-1;-1;246;244;245;243;242;238;-1;-1;239;-1;-1;-1;-1;-1;-1;-1;-1;-1;-1;-1;-1;-1;-1;-1;-1;-1;-1;-1;-1;-1;-1;-1;-1;-1;-1;-1;-1;-1;-1;-1;-1;-1;-1;-1;-1;-1;-1;-1;-1;-1;-1;-1;-1;-1;-1;-1;-1;-1;-1;-1;-1;-1;-1;-1;-1;-1;-1;-1;-1;-1;-1;-1;

998;0;

998;3;Wash Station Cleaner shallow;Wash Station Waste;Wash Station Cleaner deep;

998;;;;

998;3;;DiTi 200 ul EtOH USE;DiTi Waste;

998;;Diti 200- EtOH USE;Waste;

998;0;

998;0;

998;0;

998;0;

998;0;

998;4;DiTi 200 ul;DiTi 200 ul;DiTi 50ul 2;96 Well PCR Plate;

998;Diti 200 -A;Diti 200 -B;DiTi 50ul;Cold PCR plate;

998;0;

998;0;

998;0;

998;0;

998;0;

998;0;

998;3;DiTi 200 ul MIX1;;;

998;Diti AMP and CAPT 2;;;

998;0;

998;0;

998;0;

998;0;

998;0;

998;3;96 Well RT PCR;96 Well MAGNET PCR;;

998;RT PCR plate;MAGNET PCR;;

998;0;

998;0;

998;0;

998;0;

998;0;

998;3;Tube 8pos RT Amp1;;;

998;A1;;;

998;3;;;Trough 100ml;

998;;;E70;

998;3;;;;

998;;;;

998;3;;;;

998;;;;

998;3;;;Trough 100ml ET 2;

998;;;E100;

998;6;;0.5 ml Eppendorf Tube Enz2;;;0.5 ml Eppendorf Tube Enz;0.5 ml Eppendorf Tube Enz 3;

998;;Enz 2;;;Enz 1;Enz 3;

998;0;

998;0;

998;7;Trough 25ml SD;;96 Well Microplate HOT;;;;;

998;SD;;Extd Liquid 1 and 4;;;;;

998;0;

998;0;

998;0;

998;0;

998;0;

998;0;

998;0;

998;0;

998;0;

998;0;

998;0;

998;0;

998;0;

998;0;

998;0;

998;0;

998;0;

998;0;

998;0;

998;0;

998;0;

998;0;

998;0;

998;0;

998;0;

998;0;

998;0;

998;0;

998;0;

998;0;

998;0;

998;0;

998;0;

998;0;

998;0;

998;0;

998;0;

998;0;

998;0;

998;0;

998;0;

998;0;

998;0;

998;0;

998;0;

998;0;

998;0;

998;0;

998;0;

998;0;

998;0;

998;0;

998;0;

998;0;

998;0;

998;0;

998;0;

998;0;

998;0;

998;0;

998;0;

998;0;

998;0;

998;2;

998;86;41;

998;241;10;

998;5;

998;4;0;System;

998;0;3;Magellan;

998;0;0;Thermal Cycler;

998;0;1;Hotel 4Pos DeepWell;

998;0;2;Hotel 2Pos DitiRack 1;

998;3;

998;236;96 Well PCR Plate;

998;86;96 Well PCR Plate;

998;241;DiTi 200 ul;

998;1;

998;1;

998;45;

998;41;

998;10;

996;0;0;

--{ RPG }--

Notification(1,"EVO Team","Error: EVO 1st Information","Error",1);

Comment("Enzyme Mix:Mixing step of Enzyme was changed.Oct 19th Tera");

Comment("Enzyme Mix: thired reserver has been added.Oct 7th Tera");

Comment("50ul Diti has been applied, Oct 7th 2010 Tera");

Comment("***Start Position : Cooling Rack***");

Comment("Oxidation mix loop -> change -> RT enzyme mix loop 8th Feb Kojima");

Comment("Delete 1 times oxidation mix loop 8th Feb Kojima");

Comment("Change AMPure step 24th Feb");

Variable(TEST_MODE,"1",0,"TEST=0, REAL SUMPLE=1",0,0.000000,1.000000,0,2,0,0);

Variable(NC,"12",0,"何列分処理しますか？",0,1.000000,12.000000,0,2,0,0);

Set_DITI_Counter2("DiTi 200 ul","8","1","1",0);

Set_DITI_Counter2("DiTi 50ul 2","8","3","1",0);

Command("C5SSP800,90,0",1,1,,,2,2,0);

Group("(1) First strand Synthesis");

Comment("RNA/primer mix plate @ Four Degrees Plate (site)");

Comment("Move Four Degrees Plate --> Thermal Cycler 65 degrees ( wait 5 mins )");

Vector("FourDegreesPlate <-> SAFE","8","4",0,1,0,1,1,0);

FACTS("ThermalCycler","ThermalCycler_OpenLid","1","0","");

Vector("SAFE <> Cycler","45","1",0,1,2,0,1,0);

Vector("SAFE <> Cycler","45","1",0,0,0,1,1,0);

Vector("SAFE <> Cycler","45","1",1,0,0,2,1,0);

FACTS("ThermalCycler","ThermalCycler_CloseLid","1,0","0","");

IfThen("TEST_MODE",0,"0");

StartTimer("1");

WaitTimer("1","10");

Else();

StartTimer("1");

FACTS("ThermalCycler","ThermalCycler_RunBlock","1,FS,BLOCK,ON,300","0","");

WaitTimer("1","350");

EndIf();

FACTS("ThermalCycler","ThermalCycler_OpenLid","1","0","");

Comment("Move from Thermal Cycler --> Four Degrees Plate ( wait 1 min )");

Vector("SAFE <> Cycler","45","1",0,1,0,1,1,0);

Vector("FourDegreesPlate <-> SAFE","8","4",0,1,2,0,1,0);

Vector("FourDegreesPlate <->PUSHdown","8","4",0,1,0,0,0,0);

StartTimer("1");

WaitTimer("1","60");

Comment("Add enzyme mix 32ul");

Vector("Roma FAR RIGHT","35","5",0,1,2,2,0,0);

IfThen("TEST_MODE",0,"0");

Variable(F,"2",0,"",0,0.000000,0.000000,0,2,0,0);

Else();

Variable(F,"4",0,"",0,0.000000,0.000000,0,2,0,0);

EndIf();

Comment("Pipetting test");

Comment("AAA 10.19");

BeginLoop("F","Add RT enzyme mix (Lane 1-4) loop");

Wash(255,1,1,1,0,"5",500,"1",500,10,70,30,1,0,1000,0);

GetDITI2(255,"DiTi 50ul 2",1,0,10,70);

Aspirate(255,"Clearing BEAD contents in TIP","5","5","5","5","5","5","5","5",0,0,0,0,32,4,1,"0108ｯ1",0,0);

Mix(255,"Enzyme Liquid Level","35","35","35","35","35","35","35","35",0,0,0,0,32,4,1,"0108ｯ1",2,0,0);

Aspirate(255,"Enzyme Liquid Level2","32","32","32","32","32","32","32","32",0,0,0,0,32,4,1,"0108ｯ1",0,0);

Dispense(255,"Enzyme free dispense blow out","32","32","32","32","32","32","32","32",0,0,0,0,8,3,1,"0C08ｯ1000000000000",1,"Add RT enzyme mix (Lane 1-4) loop",0,1,0);

Aspirate(255,"Aspiration Small Vol","3","3","3","3","3","3","3","3",0,0,0,0,8,3,1,"0C08ｯ1000000000000",1,"Add RT enzyme mix (Lane 1-4) loop",0,1,0);

BeginLoop("5","RT enzyme mix loop");

Aspirate(255,"Mixing Enzyme inside Plate Slower","22","22","22","22","22","22","22","22",0,0,0,0,8,3,1,"0C08ｯ1000000000000",1,"Add RT enzyme mix (Lane 1-4) loop",0,1,0);

Dispense(255,"Mixing Enzyme inside Plate Slower","22","22","22","22","22","22","22","22",0,0,0,0,8,3,1,"0C08ｯ1000000000000",1,"Add RT enzyme mix (Lane 1-4) loop",0,1,0);

EndLoop();

Aspirate(255,"Mixing Enzyme inside Plate Slower","22","22","22","22","22","22","22","22",0,0,0,0,8,3,1,"0C08ｯ1000000000000",1,"Add RT enzyme mix (Lane 1-4) loop",0,1,0);

Dispense(255,"Mixing Enzyme inside Plate Slower2","22","22","22","22","22","22","22","22",0,0,0,0,8,3,1,"0C08ｯ1000000000000",1,"Add RT enzyme mix (Lane 1-4) loop",0,1,0);

Dispense(255,"Aspiration Small Vol","3","3","3","3","3","3","3","3",0,0,0,0,8,3,1,"0C08ｯ1000000000000",1,"Add RT enzyme mix (Lane 1-4) loop",0,1,0);

StartTimer("1");

WaitTimer("1","2");

Dispense(255,"Blow out slow","5","5","5","5","5","5","5","5",0,0,0,0,8,3,1,"0C08ｯ1000000000000",1,"Add RT enzyme mix (Lane 1-4) loop",0,1,0);

Aspirate(255,"Clearing BEAD contents in TIP","25","25","25","25","25","25","25","25",0,0,0,0,8,3,1,"0C08ｯ1000000000000",1,"Add RT enzyme mix (Lane 1-4) loop",0,1,0);

Dispense(255,"Clearing BEAD contents in TIP","25","25","25","25","25","25","25","25",0,0,0,0,8,3,1,"0C08ｯ1000000000000",1,"Add RT enzyme mix (Lane 1-4) loop",0,1,0);

Detect_Liquid(255,"Mixing Beads with Sample small fast",8,3,1,"0C08ｯ1000000000000",1,"Add RT enzyme mix (Lane 1-4) loop",0,1,0);

DropDITI(255,2,2,10,70,0);

EndLoop();

BeginLoop("F","Add RT enzyme mix (Lane 5-8) loop");

Wash(255,1,1,1,0,"2",500,"1",500,10,70,30,1,0,1000,0);

GetDITI2(255,"DiTi 50ul 2",1,0,10,70);

Aspirate(255,"Clearing BEAD contents in TIP","5","5","5","5","5","5","5","5",0,0,0,0,32,1,1,"0108ｯ1",0,0);

Mix(255,"Enzyme Liquid Level","32","32","32","32","32","32","32","32",0,0,0,0,32,1,1,"0108ｯ1",2,0,0);

Aspirate(255,"Enzyme Liquid Level2","32","32","32","32","32","32","32","32",0,0,0,0,32,1,1,"0108ｯ1",0,0);

Dispense(255,"Enzyme free dispense blow out","32","32","32","32","32","32","32","32",0,0,0,0,8,3,1,"0C080000 O00000000",1,"Add RT enzyme mix (Lane 5-8) loop",0,1,0);

Aspirate(255,"Aspiration Small Vol","3","3","3","3","3","3","3","3",0,0,0,0,8,3,1,"0C080000 O00000000",1,"Add RT enzyme mix (Lane 5-8) loop",0,1,0);

BeginLoop("5","RT enzyme mix loop");

Aspirate(255,"Mixing Enzyme inside Plate Slower","22","22","22","22","22","22","22","22",0,0,0,0,8,3,1,"0C080000 O00000000",1,"Add RT enzyme mix (Lane 5-8) loop",0,1,0);

Dispense(255,"Mixing Enzyme inside Plate Slower","22","22","22","22","22","22","22","22",0,0,0,0,8,3,1,"0C080000 O00000000",1,"Add RT enzyme mix (Lane 5-8) loop",0,1,0);

EndLoop();

Aspirate(255,"Mixing Enzyme inside Plate Slower","22","22","22","22","22","22","22","22",0,0,0,0,8,3,1,"0C080000 O00000000",1,"Add RT enzyme mix (Lane 5-8) loop",0,1,0);

Dispense(255,"Mixing Enzyme inside Plate Slower2","22","22","22","22","22","22","22","22",0,0,0,0,8,3,1,"0C080000 O00000000",1,"Add RT enzyme mix (Lane 5-8) loop",0,1,0);

Dispense(255,"Aspiration Small Vol","3","3","3","3","3","3","3","3",0,0,0,0,8,3,1,"0C080000 O00000000",1,"Add RT enzyme mix (Lane 5-8) loop",0,1,0);

StartTimer("1");

WaitTimer("1","2");

Dispense(255,"Blow out slow","5","5","5","5","5","5","5","5",0,0,0,0,8,3,1,"0C080000 O00000000",1,"Add RT enzyme mix (Lane 5-8) loop",0,1,0);

Aspirate(255,"Clearing BEAD contents in TIP","25","25","25","25","25","25","25","25",0,0,0,0,8,3,1,"0C080000 O00000000",1,"Add RT enzyme mix (Lane 5-8) loop",0,1,0);

Dispense(255,"Clearing BEAD contents in TIP","25","25","25","25","25","25","25","25",0,0,0,0,8,3,1,"0C080000 O00000000",1,"Add RT enzyme mix (Lane 5-8) loop",0,1,0);

Detect_Liquid(255,"Mixing Beads with Sample small fast",8,3,1,"0C080000 O00000000",1,"Add RT enzyme mix (Lane 5-8) loop",0,1,0);

DropDITI(255,2,2,10,70,0);

EndLoop();

BeginLoop("F","Add RT enzyme mix (Lane 9-12) loop");

Wash(255,1,1,1,0,"2",500,"1",500,10,70,30,1,0,1000,0);

GetDITI2(255,"DiTi 50ul 2",1,0,10,70);

Aspirate(255,"Clearing BEAD contents in TIP","5","5","5","5","5","5","5","5",0,0,0,0,32,5,1,"0108ｯ1",0,0);

Mix(255,"Enzyme Liquid Level","32","32","32","32","32","32","32","32",0,0,0,0,32,5,1,"0108ｯ1",2,0,0);

Aspirate(255,"Enzyme Liquid Level2","32","32","32","32","32","32","32","32",0,0,0,0,32,5,1,"0108ｯ1",0,0);

Dispense(255,"Enzyme free dispense blow out","32","32","32","32","32","32","32","32",0,0,0,0,8,3,1,"0C08000000000ｮ3000",1,"Add RT enzyme mix (Lane 9-12) loop",0,1,0);

Aspirate(255,"Aspiration Small Vol","3","3","3","3","3","3","3","3",0,0,0,0,8,3,1,"0C08000000000ｮ3000",1,"Add RT enzyme mix (Lane 9-12) loop",0,1,0);

BeginLoop("5","RT enzyme mix loop");

Aspirate(255,"Mixing Enzyme inside Plate Slower","22","22","22","22","22","22","22","22",0,0,0,0,8,3,1,"0C08000000000ｮ3000",1,"Add RT enzyme mix (Lane 9-12) loop",0,1,0);

Dispense(255,"Mixing Enzyme inside Plate Slower","22","22","22","22","22","22","22","22",0,0,0,0,8,3,1,"0C08000000000ｮ3000",1,"Add RT enzyme mix (Lane 9-12) loop",0,1,0);

EndLoop();

Aspirate(255,"Mixing Enzyme inside Plate Slower","22","22","22","22","22","22","22","22",0,0,0,0,8,3,1,"0C08000000000ｮ3000",1,"Add RT enzyme mix (Lane 9-12) loop",0,1,0);

Dispense(255,"Mixing Enzyme inside Plate Slower2","22","22","22","22","22","22","22","22",0,0,0,0,8,3,1,"0C08000000000ｮ3000",1,"Add RT enzyme mix (Lane 9-12) loop",0,1,0);

Dispense(255,"Aspiration Small Vol","3","3","3","3","3","3","3","3",0,0,0,0,8,3,1,"0C08000000000ｮ3000",1,"Add RT enzyme mix (Lane 9-12) loop",0,1,0);

StartTimer("1");

WaitTimer("1","2");

Dispense(255,"Blow out slow","5","5","5","5","5","5","5","5",0,0,0,0,8,3,1,"0C08000000000ｮ3000",1,"Add RT enzyme mix (Lane 9-12) loop",0,1,0);

Aspirate(255,"Clearing BEAD contents in TIP","25","25","25","25","25","25","25","25",0,0,0,0,8,3,1,"0C08000000000ｮ3000",1,"Add RT enzyme mix (Lane 9-12) loop",0,1,0);

Dispense(255,"Clearing BEAD contents in TIP","25","25","25","25","25","25","25","25",0,0,0,0,8,3,1,"0C08000000000ｮ3000",1,"Add RT enzyme mix (Lane 9-12) loop",0,1,0);

Detect_Liquid(255,"Mixing Beads with Sample small fast",8,3,1,"0C08000000000ｮ3000",1,"Add RT enzyme mix (Lane 9-12) loop",0,1,0);

DropDITI(255,2,2,10,70,0);

EndLoop();

Comment("End AAA 10.19");

Comment("JUMP3 new line Oct 7");

FACTS("ThermalCycler","ThermalCycler_OpenLid","1","0","");

Comment("Move Cold PCR Plate -> Thermal Cycler");

Vector("FourDegreesPlate <-> SAFE","8","4",0,1,0,1,1,0);

Vector("SAFE <> Cycler","45","1",0,1,2,0,1,0);

Vector("SAFE <> Cycler","45","1",0,0,0,1,1,0);

Vector("SAFE <> Cycler","45","1",1,0,0,2,1,0);

FACTS("ThermalCycler","ThermalCycler_CloseLid","1,0","0","");

IfThen("TEST_MODE",0,"0");

StartTimer("1");

WaitTimer("1","10");

Else();

ROMA(0,80,75,0,0,0,150,1,0);

StartTimer("1");

FACTS("ThermalCycler","ThermalCycler_RunBlock","1,001F,BLOCK,ON,3630","0","");

WaitTimer("1","3720");

EndIf();

FACTS("ThermalCycler","ThermalCycler_OpenLid","1","0","");

Comment("Move RNA/primer mix Plate to RT Assay Area 1");

Vector("SAFE <> Cycler","45","1",0,1,0,1,1,0);

Vector("RT Assay 1 <> SAFE","21","1",0,1,2,0,1,0);

Vector("RT Assay 1 <> PUSHdown","21","1",0,1,0,0,0,0);

Vector("RT Assay 1 <> SAFE","21","1",0,0,0,1,1,0);

Vector("RT Assay 1 <> SAFE","21","1",1,0,0,2,1,0);

ROMA(2,80,75,0,0,0,150,1,0);

GroupEnd();

Group("(1) AMpure purification");

Wash(255,1,1,1,0,"2",500,"1",500,10,70,30,1,0,1000,0);

PickUp_DITIs2(255,2,1,"0C08000000pｯ000000",0,"DiTi 200 ul EtOH USE",0);

Aspirate(255,"Aspirate Low Volume Liquid Level","5","5","5","5","5","5","5","5",0,0,0,0,27,0,1,"0108ｯ1",0,0);

IfThen("TEST_MODE",0,"0");

Variable(B,"2",0,"",0,0.000000,0.000000,0,2,0,0);

Else();

Variable(B,"10",0,"",0,0.000000,0.000000,0,2,0,0);

EndIf();

BeginLoop("B","Mix AMPure beads");

Aspirate(255,"Mixing for AMpure in Trough close to Z-max","180","180","180","180","180","180","180","180",0,0,0,0,27,0,1,"0108ｯ1",0,0);

Dispense(255,"Mixing AMPure dropplets high position","180","180","180","180","180","180","180","180",0,0,0,0,27,0,1,"0108ｯ1",0,0);

Mix(255,"Mixing for AMpure in Trough close to Z-max","150","150","150","150","150","150","150","150",0,0,0,0,27,0,1,"0108ｯ1",3,0,0);

EndLoop();

Dispense(255,"Low Volume wet contact dispense with tracking HIGHER Liq Positn","5","5","5","5","5","5","5","5",0,0,0,0,27,0,1,"0108ｯ1",0,0);

Detect_Liquid(255,"Contact dispense with tracking HIGH Liq Positn",27,0,1,"0108ｯ1",0,0);

DropDITI(255,2,2,10,70,0);

Comment("Renew the Script of AMPure 24th Feb Kojima ");

BeginLoop("NC","Add AMPure beads to sample and mixing");

Wash(255,1,1,1,0,"2",500,"1",500,10,70,30,1,0,1000,0);

Subroutine("C:¥Program Files¥TECAN¥EVOware¥database¥Scripts¥Diluter_Aspirate_Air.esc",0);

PickUp_DITIs2(255,15,0,"0C08ｯ1000000000000",1,"Add AMPure beads to sample and mixing",0,1,"DiTi 200 ul MIX1",0);

MoveLiha(255,15,0,1,"0C08ｯ1000000000000",4,3,25,400,1,"Add AMPure beads to sample and mixing",0,1,0);

Subroutine("C:¥Program Files¥TECAN¥EVOware¥database¥Scripts¥Diluter_Dispense_Air.esc",0);

BeginLoop("2","AMPure beads mix");

Aspirate(255,"Mixing for AMpure in Trough close to Z-max","80","80","80","80","80","80","80","80",0,0,0,0,27,0,1,"0108ｯ1",0,0);

Dispense(255,"Mixing AMPure dropplets high position","80","80","80","80","80","80","80","80",0,0,0,0,27,0,1,"0108ｯ1",0,0);

Mix(255,"Mixing for AMpure in Trough close to Z-max","80","80","80","80","80","80","80","80",0,0,0,0,27,0,1,"0108ｯ1",2,0,0);

EndLoop();

Aspirate(255,"AMPure and Beads","68.4","68.4","68.4","68.4","68.4","68.4","68.4","68.4",0,0,0,0,27,0,1,"0108ｯ1",0,0);

Detect_Liquid(255,"Contact dispense with tracking HIGH Liq Positn",27,0,1,"0108ｯ1",0,0);

Dispense(255,"AMPure and Beads","68.4","68.4","68.4","68.4","68.4","68.4","68.4","68.4",0,0,0,0,21,0,1,"0C08ｯ1000000000000",1,"Add AMPure beads to sample and mixing",0,1,0);

Detect_Liquid(255,"Contact dispense with tracking HIGH Liq Positn",21,0,1,"0C08ｯ1000000000000",1,"Add AMPure beads to sample and mixing",0,1,0);

Comment("Add Script of mixing 24th Feb Kojima");

Aspirate(255,"Aspirate Low Volume Liquid Level","5","5","5","5","5","5","5","5",0,0,0,0,21,0,1,"0C08ｯ1000000000000",1,"Add AMPure beads to sample and mixing",0,1,0);

Mix(255,"Mixing 1st Ampure inside Plate","80","80","80","80","80","80","80","80",0,0,0,0,21,0,1,"0C08ｯ1000000000000",5,1,"Add AMPure beads to sample and mixing",0,1,0);

IfThen("TEST_MODE",0,"0");

Variable(B,"3",0,"",0,0.000000,0.000000,0,2,0,0);

Else();

Variable(B,"5",0,"",0,0.000000,0.000000,0,2,0,0);

EndIf();

BeginLoop("B","AMPure and Sample mixing");

Aspirate(255,"Mixing 1st Ampure inside Plate Large Volume MIX","80","80","80","80","80","80","80","80",0,0,0,0,21,0,1,"0C08ｯ1000000000000",1,"Add AMPure beads to sample and mixing",0,1,0);

Dispense(255,"Mixing 1st Ampure inside Plate Large Volume MIX","80","80","80","80","80","80","80","80",0,0,0,0,21,0,1,"0C08ｯ1000000000000",1,"Add AMPure beads to sample and mixing",0,1,0);

EndLoop();

Dispense(255,"Low Volume wet contact dispense with tracking HIGHER Liq Positn","5","5","5","5","5","5","5","5",0,0,0,0,21,0,1,"0C08ｯ1000000000000",1,"Add AMPure beads to sample and mixing",0,1,0);

Detect_Liquid(255,"Water free dispense",21,0,1,"0C08ｯ1000000000000",1,"Add AMPure beads to sample and mixing",0,1,0);

Aspirate(255,"Clearing BEAD contents in TIP","20","20","20","20","20","20","20","20",0,0,0,0,21,0,1,"0C08ｯ1000000000000",1,"Add AMPure beads to sample and mixing",0,1,0);

Dispense(255,"Clearing BEAD contents in TIP Zmax -17","20","20","20","20","20","20","20","20",0,0,0,0,21,0,1,"0C08ｯ1000000000000",1,"Add AMPure beads to sample and mixing",0,1,0);

Set_DITIs_Back(255,15,0,"0C08ｯ1000000000000",1,"Add AMPure beads to sample and mixing",0,1,0);

EndLoop();

Comment("Remove script of 'Mix the AMPure beads and Sample 3 loops ' 24th Feb Kojima");

StartTimer("1");

WaitTimer("1","30 * 60");

Comment(" Move RT Assay Area 1 --> Magnet ( wait 5 mins )");

Vector("RT Assay 1 <> SAFE","21","1",0,1,0,1,1,0);

Vector("SAFE <> Magnet","21","2",0,1,2,0,1,0);

Vector("SAFE <> PUSH down Magnet","21","2",0,1,0,2,1,0);

Vector("Roma FAR RIGHT","35","5",0,1,2,2,1,0);

Comment("Wait10 mins for beads to fix onto Ring");

StartTimer("1");

WaitTimer("1","10 * 60");

Group("Remove supernatant and add 100 percent EtOH");

Vector("ET 2 LID <> SAFE","31","3",0,1,0,1,1,0);

BeginLoop("NC","Remove supernatant and add 100 percent EtOH");

Wash(255,1,1,1,0,"2",500,"1",500,10,70,30,1,0,1000,0);

Subroutine("C:¥Program Files¥TECAN¥EVOware¥database¥Scripts¥Diluter_Aspirate_Air.esc",0);

PickUp_DITIs2(255,15,0,"0C08ｯ1000000000000",1,"Remove supernatant and add 100 percent EtOH",0,1,"DiTi 200 ul MIX1",0);

MoveLiha(255,15,0,1,"0C08ｯ1000000000000",4,3,25,400,1,"Remove supernatant and add 100 percent EtOH",0,1,0);

Subroutine("C:¥Program Files¥TECAN¥EVOware¥database¥Scripts¥Diluter_Dispense_Air.esc",0);

Aspirate(255,"Remove Supernatant","46.4","46.4","46.4","46.4","46.4","46.4","46.4","46.4",0,0,0,0,21,1,1,"0C08ｯ1000000000000",1,"Remove supernatant and add 100 percent EtOH",0,1,0);

Dispense(255,"Clearing contents of TIP","46.4","46.4","46.4","46.4","46.4","46.4","46.4","46.4",0,0,0,0,1,1,1,"0108ｯ1",0,0);

Set_DITIs_Back(255,15,0,"0C08ｯ1000000000000",1,"Remove supernatant and add 100 percent EtOH",0,1,0);

Wash(255,1,1,1,0,"2",500,"1",500,10,70,30,1,0,1000,0);

PickUp_DITIs2(255,2,1,"0C08ｯ1000000000000",0,"DiTi 200 ul EtOH USE",0);

Mix(255,"Solvent Mix","145","145","145","145","145","145","145","145",0,0,0,0,31,2,1,"0108ｯ1",2,0,0);

Aspirate(255,"Solvent for EtOH trough","140","140","140","140","140","140","140","140",0,0,0,0,31,2,1,"0108ｯ1",0,0);

Dispense(255,"Solvent","140","140","140","140","140","140","140","140",0,0,0,0,21,1,1,"0C08ｯ1000000000000",1,"Remove supernatant and add 100 percent EtOH",0,1,0);

Set_DITIs_Back(255,2,1,"0C08ｯ1000000000000",0,0);

EndLoop();

Vector("ET 2 LID <> SAFE","31","3",0,1,2,0,1,0);

GroupEnd();

Vector("ET 1 LID <> SAFE","28","3",0,1,0,1,1,0);

Wash(255,1,1,1,0,"2",500,"1",500,10,70,30,1,0,1000,0);

BeginLoop("NC","Wash the AMPure beads and Add water for sample elution");

Comment("Remove supernatant of 100 percent EtOH");

Subroutine("C:¥Program Files¥TECAN¥EVOware¥database¥Scripts¥Diluter_Aspirate_Air.esc",0);

PickUp_DITIs2(255,15,0,"0C08ｯ1000000000000",1,"Wash the AMPure beads and Add water for sample elution",0,1,"DiTi 200 ul MIX1",0);

MoveLiha(255,15,0,1,"0C08ｯ1000000000000",4,3,25,400,1,"Wash the AMPure beads and Add water for sample elution",0,1,0);

Subroutine("C:¥Program Files¥TECAN¥EVOware¥database¥Scripts¥Diluter_Dispense_Air.esc",0);

Aspirate(255,"Aspirate EtOH Low Volume Liquid Level","70","70","70","70","70","70","70","70",0,0,0,0,21,1,1,"0C08ｯ1000000000000",1,"Wash the AMPure beads and Add water for sample elution",0,1,0);

Dispense(255,"Clearing contents of TIP","70","70","70","70","70","70","70","70",0,0,0,0,1,1,1,"0108ｯ1",0,0);

Aspirate(255,"Remove Excess liquid from Magnet Plate","110","110","110","110","110","110","110","110",0,0,0,0,21,1,1,"0C08ｯ1000000000000",1,"Wash the AMPure beads and Add water for sample elution",0,1,0);

Dispense(255,"Clearing contents of TIP","110","110","110","110","110","110","110","110",0,0,0,0,1,1,1,"0108ｯ1",0,0);

Set_DITIs_Back(255,15,0,"0C08ｯ1000000000000",1,"Wash the AMPure beads and Add water for sample elution",0,1,0);

Comment("Add 70 percent EtOH 200ul 1st");

Wash(255,1,1,1,0,"2",500,"1",500,10,70,30,1,0,1000,0);

PickUp_DITIs2(255,2,1,"0C08ｯ1000000000000",0,"DiTi 200 ul EtOH USE",0);

Mix(255,"Solvent Mix","100","100","100","100","100","100","100","100",0,0,0,0,28,2,1,"0108ｯ1",2,0,0);

Aspirate(255,"Solvent for EtOH trough","100","100","100","100","100","100","100","100",0,0,0,0,28,2,1,"0108ｯ1",0,0);

Dispense(255,"Solvent","100","100","100","100","100","100","100","100",0,0,0,0,21,1,1,"0C08ｯ1000000000000",1,"Wash the AMPure beads and Add water for sample elution",0,1,0);

Aspirate(255,"Solvent for EtOH trough","100","100","100","100","100","100","100","100",0,0,0,0,28,2,1,"0108ｯ1",0,0);

Dispense(255,"Solvent","100","100","100","100","100","100","100","100",0,0,0,0,21,1,1,"0C08ｯ1000000000000",1,"Wash the AMPure beads and Add water for sample elution",0,1,0);

Detect_Liquid(255,"Solvent",28,2,1,"0108ｯ1",0,0);

Set_DITIs_Back(255,2,1,"0C08ｯ1000000000000",0,0);

Comment("Remove supernatant of 70 percent EtOH 1st");

Wash(255,1,1,1,0,"2",500,"1",500,10,70,30,1,0,1000,0);

Subroutine("C:¥Program Files¥TECAN¥EVOware¥database¥Scripts¥Diluter_Aspirate_Air.esc",0);

PickUp_DITIs2(255,15,0,"0C08ｯ1000000000000",1,"Wash the AMPure beads and Add water for sample elution",0,1,"DiTi 200 ul MIX1",0);

MoveLiha(255,15,0,1,"0C08ｯ1000000000000",4,3,25,400,1,"Wash the AMPure beads and Add water for sample elution",0,1,0);

Subroutine("C:¥Program Files¥TECAN¥EVOware¥database¥Scripts¥Diluter_Dispense_Air.esc",0);

Aspirate(255,"Aspirate EtOH Low Volume Liquid Level","120","120","120","120","120","120","120","120",0,0,0,0,21,1,1,"0C08ｯ1000000000000",1,"Wash the AMPure beads and Add water for sample elution",0,1,0);

Dispense(255,"Clearing BEAD contents in TIP","120","120","120","120","120","120","120","120",0,0,0,0,1,1,1,"0108ｯ1",0,0);

Aspirate(255,"Solvent without asp airgap at end","130","130","130","130","130","130","130","130",0,0,0,0,21,1,1,"0C08ｯ1000000000000",1,"Wash the AMPure beads and Add water for sample elution",0,1,0);

Dispense(255,"Clearing BEAD contents in TIP","130","130","130","130","130","130","130","130",0,0,0,0,1,1,1,"0108ｯ1",0,0);

Set_DITIs_Back(255,15,0,"0C08ｯ1000000000000",1,"Wash the AMPure beads and Add water for sample elution",0,1,0);

Comment("Add 70 percent EtOH 200ul 2nd");

Wash(255,1,1,1,0,"2",500,"1",500,10,70,30,1,0,1000,0);

PickUp_DITIs2(255,2,1,"0C08ｯ1000000000000",0,"DiTi 200 ul EtOH USE",0);

Aspirate(255,"Solvent for EtOH trough","100","100","100","100","100","100","100","100",0,0,0,0,28,2,1,"0108ｯ1",0,0);

Dispense(255,"Solvent","100","100","100","100","100","100","100","100",0,0,0,0,21,1,1,"0C08ｯ1000000000000",1,"Wash the AMPure beads and Add water for sample elution",0,1,0);

Aspirate(255,"Solvent for EtOH trough","100","100","100","100","100","100","100","100",0,0,0,0,28,2,1,"0108ｯ1",0,0);

Dispense(255,"Solvent","100","100","100","100","100","100","100","100",0,0,0,0,21,1,1,"0C08ｯ1000000000000",1,"Wash the AMPure beads and Add water for sample elution",0,1,0);

Detect_Liquid(255,"Solvent",28,2,1,"0108ｯ1",0,0);

Set_DITIs_Back(255,2,1,"0C08ｯ1000000000000",0,0);

Comment("Remove supernatant of 70 percent EtOH 2nd");

Wash(255,1,1,1,0,"2",500,"1",500,10,70,30,1,0,1000,0);

Subroutine("C:¥Program Files¥TECAN¥EVOware¥database¥Scripts¥Diluter_Aspirate_Air.esc",0);

PickUp_DITIs2(255,15,0,"0C08ｯ1000000000000",1,"Wash the AMPure beads and Add water for sample elution",0,1,"DiTi 200 ul MIX1",0);

MoveLiha(255,15,0,1,"0C08ｯ1000000000000",4,3,25,400,1,"Wash the AMPure beads and Add water for sample elution",0,1,0);

Subroutine("C:¥Program Files¥TECAN¥EVOware¥database¥Scripts¥Diluter_Dispense_Air.esc",0);

Aspirate(255,"Aspirate EtOH Low Volume Liquid Level","120","120","120","120","120","120","120","120",0,0,0,0,21,1,1,"0C08ｯ1000000000000",1,"Wash the AMPure beads and Add water for sample elution",0,1,0);

Dispense(255,"Clearing BEAD contents in TIP","120","120","120","120","120","120","120","120",0,0,0,0,1,1,1,"0108ｯ1",0,0);

Aspirate(255,"Solvent without asp airgap at end","70","70","70","70","70","70","70","70",0,0,0,0,21,1,1,"0C08ｯ1000000000000",1,"Wash the AMPure beads and Add water for sample elution",0,1,0);

Dispense(255,"Clearing BEAD contents in TIP","70","70","70","70","70","70","70","70",0,0,0,0,1,1,1,"0108ｯ1",0,0);

Aspirate(255,"Solvent","70","70","70","70","70","70","70","70",0,0,0,0,21,1,1,"0C08ｯ1000000000000",1,"Wash the AMPure beads and Add water for sample elution",0,1,0);

Dispense(255,"Clearing BEAD contents in TIP","70","70","70","70","70","70","70","70",0,0,0,0,1,1,1,"0108ｯ1",0,0);

Aspirate(255,"Clearing contents of TIP","100","100","100","100","100","100","100","100",0,0,0,0,1,1,1,"0108ｯ1",0,0);

Dispense(255,"Clearing contents of TIP variation","100","100","100","100","100","100","100","100",0,0,0,0,1,1,1,"0108ｯ1",0,0);

Set_DITIs_Back(255,15,0,"0C08ｯ1000000000000",1,"Wash the AMPure beads and Add water for sample elution",0,1,0);

Comment("Add water 42ul for sample elution");

Wash(255,1,1,1,0,"2",500,"1",500,10,70,30,1,0,1000,0);

PickUp_DITIs2(255,2,1,"0C0800ｬ70000000000",0,"DiTi 200 ul EtOH USE",0);

Aspirate(255,"SD Water free dispense","42","42","42","42","42","42","42","42",0,0,0,0,35,0,1,"0108ｯ1",0,0);

Dispense(255,"SD Water free dispense","42","42","42","42","42","42","42","42",0,0,0,0,21,1,1,"0C08ｯ1000000000000",1,"Wash the AMPure beads and Add water for sample elution",0,1,0);

Set_DITIs_Back(255,2,1,"0C0800ｬ70000000000",0,0);

EndLoop();

Vector("ET 1 LID <> SAFE","28","3",0,1,2,0,1,0);

Wash(255,1,1,1,0,"2",500,"1",500,10,70,30,1,0,1000,0);

Comment("Move from Magnet --> RT Assay Area 1");

Vector("SAFE <> Magnet","21","2",0,1,0,1,1,0);

Vector("MAGNET SHIFTING","21","2",0,0,2,2,1,0);

StartTimer("1");

WaitTimer("1","60");

Vector("MAGNET SHIFTING","21","2",1,0,2,2,1,0);

Vector("RT Assay 1 <> SAFE","21","1",0,1,2,0,1,0);

Vector("RT Assay 1 <> PUSHdown","21","1",0,1,0,0,1,0);

ROMA(2,80,75,0,0,0,150,1,0);

Notification(1,"EVO Team","Confirmation:Plate stand: EVO 1st Information","Check Plate! About 30 min before",0);

BeginLoop("NC","Mix the sample for elution");

Wash(255,1,1,1,0,"2",500,"1",500,10,70,30,1,0,1000,0);

GetDITI2(255,"DiTi 200 ul",1,0,10,70);

Aspirate(255,"Mixing Beads with Sample small volume aspirate","5","5","5","5","5","5","5","5",0,0,0,0,21,0,1,"0C08ｯ1000000000000",1,"Mix the sample for elution",0,1,0);

IfThen("TEST_MODE",0,"0");

Variable(D,"5",0,"",0,0.000000,0.000000,0,2,0,0);

Else();

Variable(D,"30",0,"",0,0.000000,0.000000,0,2,0,0);

EndIf();

BeginLoop("D","Sample mixing");

Aspirate(255,"Mixing Beads with Sample small fast","25","25","25","25","25","25","25","25",0,0,0,0,21,0,1,"0C08ｯ1000000000000",1,"Mix the sample for elution",0,1,0);

Dispense(255,"Mixing Beads with Sample small fast","25","25","25","25","25","25","25","25",0,0,0,0,21,0,1,"0C08ｯ1000000000000",1,"Mix the sample for elution",0,1,0);

EndLoop();

Dispense(255,"Mixing Beads with Sample small volume aspirate","5","5","5","5","5","5","5","5",0,0,0,0,21,0,1,"0C08ｯ1000000000000",1,"Mix the sample for elution",0,1,0);

Detect_Liquid(255,"Mixing Beads with Sample small fast",21,0,1,"0C08ｯ1000000000000",1,"Mix the sample for elution",0,1,0);

Aspirate(255,"Clearing BEAD contents in TIP","20","20","20","20","20","20","20","20",0,0,0,0,21,0,1,"0C08ｯ1000000000000",1,"Mix the sample for elution",0,1,0);

Dispense(255,"Clearing BEAD contents in TIP Zmax -17","20","20","20","20","20","20","20","20",0,0,0,0,21,0,1,"0C08ｯ1000000000000",1,"Mix the sample for elution",0,1,0);

DropDITI(255,2,2,10,70,0);

EndLoop();

Wash(255,1,1,1,0,"2",500,"1",500,10,70,30,1,0,1000,0);

Comment("Move RT Assay Area 1 --> Thermal cycler (wait 5 mins)");

FACTS("ThermalCycler","ThermalCycler_OpenLid","1","0","");

Vector("RT Assay 1 <> SAFE","21","1",0,1,0,1,1,0);

Vector("SAFE <> Cycler","45","1",0,1,2,0,1,0);

FACTS("ThermalCycler","ThermalCycler_CloseLid","1,0","0","");

Comment("10.25 Stand(Position) was changed");

Comment("Supply New 96 plate");

Notification(1,"EVO Team","Confirmation:Plate stand: EVO 1st Information","Check Plate!",0);

Vector("ExtractedLiquid <> SAFE","35","3",0,1,0,1,1,0);

Vector("RT Assay 1 <> SAFE","21","1",0,1,2,0,1,0);

Vector("RT Assay 1 <> PUSHdown","21","1",0,1,0,0,1,0);

ROMA(2,80,75,0,0,0,150,1,0);

Comment("10.25 Stand(Position) was changed");

IfThen("TEST_MODE",0,"0");

StartTimer("1");

WaitTimer("1","10");

Else();

StartTimer("1");

FACTS("ThermalCycler","ThermalCycler_RunBlock","1,SD42,BLOCK,ON,300","0","");

WaitTimer("1","315");

EndIf();

FACTS("ThermalCycler","ThermalCycler_OpenLid","1","0","");

Comment("Thermal cycler --> Magnet (wait 10 mins)");

Vector("SAFE <> Cycler","45","1",0,1,0,1,1,0);

Vector("SAFE <> Magnet","21","2",0,1,2,0,1,0);

Vector("SAFE <> PUSH down Magnet","21","2",0,1,0,2,1,0);

Vector("Roma FAR RIGHT","35","5",0,1,2,2,0,0);

StartTimer("1");

WaitTimer("1","10 * 60");

BeginLoop("NC","Transfer the sample to New plate");

Wash(255,1,1,1,0,"2",500,"1",500,10,70,30,1,0,1000,0);

GetDITI2(255,"DiTi 200 ul",0,0,10,70);

Aspirate(255,"Left-over","47","47","47","47","47","47","47","47",0,0,0,0,21,1,1,"0C08ｯ1000000000000",1,"Transfer the sample to New plate",0,1,0);

Dispense(255,"AMpure Collection","47","47","47","47","47","47","47","47",0,0,0,0,21,0,1,"0C08ｯ1000000000000",1,"Transfer the sample to New plate",0,1,0);

DropDITI(255,2,2,10,70,0);

EndLoop();

Comment("10.25 Stand(Position) was changed");

Comment("Incubate for 10min on 37 degree for remove contaminated EtOH");

Vector("RT Assay 1 <> SAFE","21","1",0,1,0,1,1,0);

Vector("ExtractedLiquid <> SAFE","35","3",0,1,2,0,1,0);

Vector("ExtractedLiquid <> PUSHdown","35","3",0,1,0,0,1,0);

ROMA(2,80,75,0,0,0,150,1,0);

Comment("10.25 Stand(Position) was changed");

StartTimer("1");

WaitTimer("1","10 * 60");

Vector("ExtractedLiquid <> SAFE","35","3",0,1,0,1,1,0);

Vector("FourDegreesPlate <-> SAFE","8","4",0,1,2,0,1,0);

Vector("FourDegreesPlate <->PUSHdown","8","4",0,1,0,0,1,0);

ROMA(2,80,75,0,0,0,150,1,0);

If("1",0,"1","SKIP");

Comment("Let Sample Plate cool at 4 degrees for 2 mins");

Comment("REPLACE DITI BOX 1");

Comment("Dispose Obsolete plate from Magnet");

Vector("SAFE <> Magnet","21","2",0,1,0,1,1,0);

Vector("Dispose OLD Magnet Plate","21","2",0,1,2,0,1,0);

Vector("Transfer Diti 200 @ Cooling Carrier","8","1",0,1,0,1,1,0);

Vector("Transfer Diti Slot A","10","1",0,1,2,0,1,0);

Vector("Transfer Diti 200 @ Cooling Carrier","8","2",0,1,0,1,1,0);

Vector("Transfer Diti Slot A","10","1",0,1,2,0,1,0);

Vector("Transfer Diti 200 @ Cooling Carrier","15","2",0,1,0,1,1,0);

Vector("Transfer Diti Slot A","10","1",0,1,2,0,1,0);

Vector("Transfer Diti Slot B","10","1",0,1,0,1,1,0);

Vector("Transfer Diti 200 @ Cooling Carrier","8","1",0,1,2,0,1,0);

Vector("Transfer Diti Slot B","10","1",0,1,0,1,1,0);

Vector("Transfer Diti 200 @ Cooling Carrier","8","2",0,1,2,0,1,0);

Vector("Transfer Diti Slot B","10","1",0,1,0,1,1,0);

Vector("Transfer Diti 200 @ Cooling Carrier","15","2",0,1,2,0,1,0);

Comment("SKIP");

ROMA(2,80,75,0,0,0,150,1,0);

GroupEnd();

Notification(0,"EVO Team","Assay finish","Assay finish",0);

**---END---**

**2. OXIDATION_and_PURIFICATION**

**---START---**

67E23C01

20110620_114207 Admin

Administrator

--{ RES }--

V;200

--{ CFG }--

999;219;32;

14;-1;30;8;-1;-1;-1;-1;-1;234;-1;-1;-1;-1;-1;-1;96;-1;-1;-1;-1;-1;34;-1;-1;-1;-1;-1;246;244;245;243;242;238;-1;-1;239;-1;-1;-1;-1;-1;-1;-1;-1;-1;-1;-1;-1;-1;-1;-1;-1;-1;-1;-1;-1;-1;-1;-1;-1;-1;-1;-1;-1;-1;-1;-1;-1;-1;-1;-1;-1;-1;-1;-1;-1;-1;-1;-1;-1;-1;-1;-1;-1;-1;-1;-1;-1;-1;-1;-1;-1;-1;-1;-1;-1;-1;-1;-1;

998;0;

998;3;Wash Station Cleaner shallow;Wash Station Waste;Wash Station Cleaner deep;

998;;;;

998;3;;DiTi 200 ul EtOH USE;DiTi Waste;

998;;Diti 200- EtOH USE;Waste;

998;0;

998;0;

998;0;

998;0;

998;0;

998;4;DiTi 200 ul;DiTi 200 ul;DiTi 50ul 2;96 Well PCR Plate;

998;Diti 200 -1;Diti 200 -2;DiTi 50ul;Cold PCR plate;

998;0;

998;0;

998;0;

998;0;

998;0;

998;0;

998;3;;DiTi 200 ul MIX2;;

998;;Diti AMP and CAPT 3;;

998;0;

998;0;

998;0;

998;0;

998;0;

998;3;96 Well RT PCR;96 Well MAGNET PCR;;

998;RT PCR plate;MAGNET PCR;;

998;0;

998;0;

998;0;

998;0;

998;0;

998;3;;;Tube 8pos RT Amp3;

998;;;A2;

998;3;;;Trough 100ml;

998;;;E70;

998;3;;Trough 25ml OxS;;

998;;OxS;;

998;3;;Trough 100ml ISO;;

998;;Iso;;

998;3;;;Trough 100ml ET 2;

998;;;E100;

998;6;;;;0.5 ml Eppendorf Tube RH+RHO;;;

998;;;;OXIdation;;;

998;0;

998;0;

998;7;Trough 25ml SD;;;;96 Well Microplate HOT;;;

998;SD;;;;Extd Liquid 2 and 5;;;

998;0;

998;0;

998;0;

998;0;

998;0;

998;0;

998;0;

998;0;

998;0;

998;0;

998;0;

998;0;

998;0;

998;0;

998;0;

998;0;

998;0;

998;0;

998;0;

998;0;

998;0;

998;0;

998;0;

998;0;

998;0;

998;0;

998;0;

998;0;

998;0;

998;0;

998;0;

998;0;

998;0;

998;0;

998;0;

998;0;

998;0;

998;0;

998;0;

998;0;

998;0;

998;0;

998;0;

998;0;

998;0;

998;0;

998;0;

998;0;

998;0;

998;0;

998;0;

998;0;

998;0;

998;0;

998;0;

998;0;

998;0;

998;0;

998;0;

998;0;

998;0;

998;0;

998;0;

998;2;

998;86;41;

998;241;10;

998;5;

998;4;0;System;

998;0;3;Magellan;

998;0;0;Thermal Cycler;

998;0;1;Hotel 4Pos DeepWell;

998;0;2;Hotel 2Pos DitiRack 1;

998;3;

998;236;96 Well PCR Plate;

998;86;96 Well PCR Plate;

998;241;DiTi 200 ul;

998;1;

998;1;

998;45;

998;41;

998;10;

996;0;0;

--{ RPG }--

Notification(1,"EVO Team","Error: EVO 1st Information","Error",1);

Comment("Mixing step of Enzyme was changed.Oct 19th Tera");

Comment("50ul Diti has been applied, Oct 7th 2010 Tera");

Comment("***Start Position : Cooling Rack***");

Comment("Loop name changed 9th Feb Kojima");

Comment("Renew the Script of AMPure 1st Mar Kojima ");

Comment("Correct the volume of AMPure mix 100uL --> 150uL 2nd Mar Kojima ");

Variable(TEST_MODE,"1",0,"TEST=0, REAL SUMPLE=1",0,0.000000,1.000000,0,2,0,0);

Variable(NC,"12",0,"何列分処理しますか？",0,1.000000,12.000000,0,2,0,0);

Set_DITI_Counter2("DiTi 200 ul","8","1","1",0);

Set_DITI_Counter2("DiTi 50ul 2","8","3","1",0);

Command("C5SSP800,90,0",1,1,,,2,2,0);

ROMA(2,80,75,0,0,0,150,1,0);

Group("(2) Oxidation");

Comment("Add Oxidation mix 4ul");

Vector("Ox Lid <> SAFE","32","4",0,1,0,1,0,0);

Comment("modified 10.19.2010");

BeginLoop("NC","Add Oxidation mix loop");

Wash(255,1,1,1,0,"2",500,"1",500,10,70,30,1,0,1000,0);

GetDITI2(255,"DiTi 50ul 2",1,0,10,70);

Aspirate(255,"Clearing BEAD contents in TIP","5","5","5","5","5","5","5","5",0,0,0,0,32,3,1,"0108ｯ1",0,0);

Mix(255,"Enzyme Liquid Level","6","6","6","6","6","6","6","6",0,0,0,0,32,3,1,"0108ｯ1",2,0,0);

Aspirate(255,"Oxidation Aspiration from Small tube2","4","4","4","4","4","4","4","4",0,0,0,0,32,3,1,"0108ｯ1",0,0);

Dispense(255,"Oxidation Aspiration from Small tube2","4","4","4","4","4","4","4","4",0,0,0,0,8,3,1,"0C08ｯ1000000000000",1,"Add Oxidation mix loop",0,1,0);

Aspirate(255,"Aspiration Small Vol","3","3","3","3","3","3","3","3",0,0,0,0,8,3,1,"0C08ｯ1000000000000",1,"Add Oxidation mix loop",0,1,0);

BeginLoop("4","Oxidation mix loop");

Aspirate(255,"Mixing OxiMix inside Plate Slower2","30","30","30","30","30","30","30","30",0,0,0,0,8,3,1,"0C08ｯ1000000000000",1,"Add Oxidation mix loop",0,1,0);

Dispense(255,"Mixing OxiMix inside Plate Slower2","30","30","30","30","30","30","30","30",0,0,0,0,8,3,1,"0C08ｯ1000000000000",1,"Add Oxidation mix loop",0,1,0);

EndLoop();

Aspirate(255,"Mixing OxiMix inside Plate Slower2","30","30","30","30","30","30","30","30",0,0,0,0,8,3,1,"0C08ｯ1000000000000",1,"Add Oxidation mix loop",0,1,0);

Dispense(255,"Mixing OxiMix inside Plate Slower3","30","30","30","30","30","30","30","30",0,0,0,0,8,3,1,"0C08ｯ1000000000000",1,"Add Oxidation mix loop",0,1,0);

Dispense(255,"Aspiration Small Vol","3","3","3","3","3","3","3","3",0,0,0,0,8,3,1,"0C08ｯ1000000000000",1,"Add Oxidation mix loop",0,1,0);

StartTimer("1");

WaitTimer("1","2");

Dispense(255,"Blow out slow","5","5","5","5","5","5","5","5",0,0,0,0,8,3,1,"0C08ｯ1000000000000",1,"Add Oxidation mix loop",0,1,0);

Aspirate(255,"Clearing BEAD contents in TIP","25","25","25","25","25","25","25","25",0,0,0,0,8,3,1,"0C08ｯ1000000000000",1,"Add Oxidation mix loop",0,1,0);

Dispense(255,"Clearing BEAD contents in TIP","25","25","25","25","25","25","25","25",0,0,0,0,8,3,1,"0C08ｯ1000000000000",1,"Add Oxidation mix loop",0,1,0);

Detect_Liquid(255,"Detect liqid",8,3,1,"0C08ｯ1000000000000",1,"Add Oxidation mix loop",0,1,0);

DropDITI(255,2,2,10,70,0);

EndLoop();

Comment("End modified 10.19.2010");

Vector("Ox Lid <> SAFE","32","4",0,1,2,0,1,0);

FACTS("ThermalCycler","ThermalCycler_OpenLid","1","0","");

Comment("Move Extracted Liquid 1 --> T.Cycler : 4 degrees In DARKness ( wait 45 mins )");

Vector("FourDegreesPlate <-> SAFE","8","4",0,1,0,1,1,0);

Vector("SAFE <> Cycler","45","1",0,1,2,0,1,0);

Vector("SAFE <> Cycler","45","1",0,0,0,1,1,0);

Vector("SAFE <> Cycler","45","1",1,0,0,2,1,0);

FACTS("ThermalCycler","ThermalCycler_CloseLid","1,0","0","");

IfThen("TEST_MODE",0,"0");

StartTimer("1");

WaitTimer("1","10");

Else();

StartTimer("1");

FACTS("ThermalCycler","ThermalCycler_RunBlock","1,OXID,BLOCK,OFF,2700","0","");

WaitTimer("1","2740");

EndIf();

FACTS("ThermalCycler","ThermalCycler_OpenLid","1","0","");

Comment("Move T.Cycler --> Four Degrees Plate");

Vector("SAFE <> Cycler","45","1",0,1,0,1,1,0);

Vector("RT Assay 1 <> SAFE","21","1",0,1,2,0,1,0);

Vector("RT Assay 1 <> PUSHdown","21","1",0,1,0,0,1,0);

Vector("RT Assay 1 <> SAFE","21","1",0,0,0,1,1,0);

Vector("RT Assay 1 <> SAFE","21","1",1,0,0,2,1,0);

ROMA(2,80,75,0,0,0,150,1,0);

Comment("Oxidation stop 16ul");

Comment("Modified 10.19.2010");

BeginLoop("NC","Add Oxidation stop mix loop");

Wash(255,1,1,1,0,"5",500,"1",500,10,70,30,1,0,1000,0);

GetDITI2(255,"DiTi 200 ul",1,0,10,70);

Aspirate(255,"Clearing BEAD contents in TIP 2","20","20","20","20","20","20","20","20",0,0,0,0,29,1,1,"0108ｯ1",0,0);

Mix(255,"Enzyme Liquid Level","16","16","16","16","16","16","16","16",0,0,0,0,29,1,1,"0108ｯ1",2,0,0);

Aspirate(255,"Oxidation stop mix Aspiration from Small tube2","16","16","16","16","16","16","16","16",0,0,0,0,29,1,1,"0108ｯ1",0,0);

Dispense(255,"Oxidation stop mix Aspiration from Small tube2","16","16","16","16","16","16","16","16",0,0,0,0,21,0,1,"0C08ｯ1000000000000",1,"Add Oxidation stop mix loop",0,1,0);

Aspirate(255,"Aspiration Small Vol","8","8","8","8","8","8","8","8",0,0,0,0,21,0,1,"0C08ｯ1000000000000",1,"Add Oxidation stop mix loop",0,1,0);

BeginLoop("4","Oxidation stop mix loop");

Aspirate(255,"Mixing OxiMix inside Plate Slower2","42","42","42","42","42","42","42","42",0,0,0,0,21,0,1,"0C08ｯ1000000000000",1,"Add Oxidation stop mix loop",0,1,0);

Dispense(255,"Mixing OxiMix inside Plate Slower2","42","42","42","42","42","42","42","42",0,0,0,0,21,0,1,"0C08ｯ1000000000000",1,"Add Oxidation stop mix loop",0,1,0);

EndLoop();

Aspirate(255,"Mixing OxiMix inside Plate Slower2","42","42","42","42","42","42","42","42",0,0,0,0,21,0,1,"0C08ｯ1000000000000",1,"Add Oxidation stop mix loop",0,1,0);

Dispense(255,"Mixing OxiMix inside Plate Slower3","42","42","42","42","42","42","42","42",0,0,0,0,21,0,1,"0C08ｯ1000000000000",1,"Add Oxidation stop mix loop",0,1,0);

Dispense(255,"Aspiration Small Vol","8","8","8","8","8","8","8","8",0,0,0,0,21,0,1,"0C08ｯ1000000000000",1,"Add Oxidation stop mix loop",0,1,0);

StartTimer("1");

WaitTimer("1","2");

Dispense(255,"Blow out slow_RNaseH&ONE","20","20","20","20","20","20","20","20",0,0,0,0,21,0,1,"0C08ｯ1000000000000",1,"Add Oxidation stop mix loop",0,1,0);

Aspirate(255,"Clearing BEAD contents in TIP","25","25","25","25","25","25","25","25",0,0,0,0,21,0,1,"0C08ｯ1000000000000",1,"Add Oxidation stop mix loop",0,1,0);

Dispense(255,"Clearing BEAD contents in TIP_Z-max -9","25","25","25","25","25","25","25","25",0,0,0,0,21,0,1,"0C08ｯ1000000000000",1,"Add Oxidation stop mix loop",0,1,0);

Detect_Liquid(255,"Detect liqid",21,0,1,"0C08ｯ1000000000000",1,"Add Oxidation stop mix loop",0,1,0);

DropDITI(255,2,2,10,70,0);

EndLoop();

Comment("End Modified 10.19.2010");

GroupEnd();

Group("Add Isopropanol to sample ");

Vector("Iso LID <> SAFE","30","2",0,1,0,1,1,0);

Comment("Isopropanol 15ul ");

BeginLoop("NC","Add Isopropanol to sample loop");

DropDITI(255,2,2,10,70,0);

Wash(255,1,1,1,0,"2",500,"1",500,10,70,30,1,0,1000,0);

GetDITI2(255,"DiTi 200 ul",1,0,10,70);

Mix(255,"Solvent Mix","15","15","15","15","15","15","15","15",0,0,0,0,30,1,1,"0108ｯ1",2,0,0);

Aspirate(255,"ISO wet contact dispense","15","15","15","15","15","15","15","15",0,0,0,0,30,1,1,"0108ｯ1",0,0);

Dispense(255,"Solvent ISO","15","15","15","15","15","15","15","15",0,0,0,0,21,0,1,"0C08ｯ1000000000000",1,"Add Isopropanol to sample loop",0,1,0);

Aspirate(255,"Aspirate Low Volume further lower Liquid Level","3","3","3","3","3","3","3","3",0,0,0,0,21,0,1,"0C08ｯ1000000000000",1,"Add Isopropanol to sample loop",0,1,0);

Mix(255,"Mixing Enzyme inside Plate","50","50","50","50","50","50","50","50",0,0,0,0,21,0,1,"0C08ｯ1000000000000",5,1,"Add Isopropanol to sample loop",0,1,0);

Dispense(255,"Low Volume wet contact dispense at Liquid Level","3","3","3","3","3","3","3","3",0,0,0,0,21,0,1,"0C08ｯ1000000000000",1,"Add Isopropanol to sample loop",0,1,0);

Detect_Liquid(255,"Mixing Beads with Sample small fast",21,0,1,"0C08ｯ1000000000000",1,"Add Isopropanol to sample loop",0,1,0);

DropDITI(255,2,2,10,70,0);

EndLoop();

Vector("Iso LID <> SAFE","30","2",0,1,2,0,1,0);

Comment("Renew the Script of AMPure 1st Mar Kojima ");

Group("(2) AMpure purification");

Wash(255,1,1,1,0,"2",500,"1",500,10,70,30,1,0,1000,0);

PickUp_DITIs2(255,2,1,"0C0800000000ｯ10000",0,"DiTi 200 ul EtOH USE",0);

Aspirate(255,"Aspirate Low Volume Liquid Level","5","5","5","5","5","5","5","5",0,0,0,0,27,2,1,"0108ｯ1",0,0);

IfThen("TEST_MODE",0,"0");

Variable(B,"5",0,"",0,0.000000,0.000000,0,2,0,0);

Else();

Variable(B,"10",0,"",0,0.000000,0.000000,0,2,0,0);

EndIf();

BeginLoop("B","Mix AMPure beads");

Aspirate(255,"Mixing for AMpure in Trough close to Z-max","180","180","180","180","180","180","180","180",0,0,0,0,27,2,1,"0108ｯ1",0,0);

Dispense(255,"Mixing AMPure dropplets high position","180","180","180","180","180","180","180","180",0,0,0,0,27,2,1,"0108ｯ1",0,0);

Mix(255,"Mixing for AMpure in Trough close to Z-max","150","150","150","150","150","150","150","150",0,0,0,0,27,2,1,"0108ｯ1",3,0,0);

EndLoop();

Dispense(255,"Low Volume wet contact dispense with tracking HIGHER Liq Positn","5","5","5","5","5","5","5","5",0,0,0,0,27,2,1,"0108ｯ1",0,0);

Detect_Liquid(255,"Contact dispense with tracking HIGH Liq Positn",27,2,1,"0108ｯ1",0,0);

DropDITI(255,2,2,10,70,0);

BeginLoop("NC","Add AMPure beads to sample and Mixing");

Wash(255,1,1,1,0,"2",500,"1",500,10,70,30,1,0,1000,0);

Subroutine("C:¥Program Files¥TECAN¥EVOware¥database¥Scripts¥Diluter_Aspirate_Air.esc",0);

PickUp_DITIs2(255,15,1,"0C08ｯ1000000000000",1,"Add AMPure beads to sample and Mixing",0,1,"DiTi 200 ul MIX2",0);

MoveLiha(255,15,1,1,"0C08ｯ1000000000000",4,3,25,400,1,"Add AMPure beads to sample and Mixing",0,1,0);

Subroutine("C:¥Program Files¥TECAN¥EVOware¥database¥Scripts¥Diluter_Dispense_Air.esc",0);

BeginLoop("2","AMPure beads mix");

Aspirate(255,"Mixing for AMpure in Trough close to Z-max","80","80","80","80","80","80","80","80",0,0,0,0,27,2,1,"0108ｯ1",0,0);

Dispense(255,"Mixing AMPure dropplets high position","80","80","80","80","80","80","80","80",0,0,0,0,27,2,1,"0108ｯ1",0,0);

Mix(255,"Mixing for AMpure in Trough close to Z-max","80","80","80","80","80","80","80","80",0,0,0,0,27,2,1,"0108ｯ1",3,0,0);

EndLoop();

Aspirate(255,"AMPure and Beads","135","135","135","135","135","135","135","135",0,0,0,0,27,2,1,"0108ｯ1",0,0);

Detect_Liquid(255,"AMPure and Beads",27,2,1,"0108ｯ1",0,0);

Dispense(255,"AMPure and Beads","135","135","135","135","135","135","135","135",0,0,0,0,21,0,1,"0C08ｯ1000000000000",1,"Add AMPure beads to sample and Mixing",0,1,0);

Detect_Liquid(255,"AMPure and Beads",21,0,1,"0C08ｯ1000000000000",1,"Add AMPure beads to sample and Mixing",0,1,0);

Comment("Renew the Script of AMPure 24th Feb Kojima ");

Aspirate(255,"Aspirate Low Volume Liquid Level","5","5","5","5","5","5","5","5",0,0,0,0,21,0,1,"0C08ｯ1000000000000",1,"Add AMPure beads to sample and Mixing",0,1,0);

Mix(255,"Mixing Ampure inside Plate","150","150","150","150","150","150","150","150",0,0,0,0,21,0,1,"0C08ｯ1000000000000",5,1,"Add AMPure beads to sample and Mixing",0,1,0);

IfThen("TEST_MODE",0,"0");

Variable(B,"3",0,"",0,0.000000,0.000000,0,2,0,0);

Else();

Variable(B,"5",0,"",0,0.000000,0.000000,0,2,0,0);

EndIf();

BeginLoop("B","AMPure beads and sample mixing");

Aspirate(255,"Mixing 2nd Ampure inside Plate Large Volume MIX","150","150","150","150","150","150","150","150",0,0,0,0,21,0,1,"0C08ｯ1000000000000",1,"Add AMPure beads to sample and Mixing",0,1,0);

Dispense(255,"Mixing 2nd Ampure inside Plate Large Volume MIX","150","150","150","150","150","150","150","150",0,0,0,0,21,0,1,"0C08ｯ1000000000000",1,"Add AMPure beads to sample and Mixing",0,1,0);

EndLoop();

Dispense(255,"Low Volume wet contact dispense with tracking HIGHER Liq Positn","5","5","5","5","5","5","5","5",0,0,0,0,21,0,1,"0C08ｯ1000000000000",1,"Add AMPure beads to sample and Mixing",0,1,0);

Detect_Liquid(255,"Mixing Ampure inside Plate Large Volume MIX",21,0,1,"0C08ｯ1000000000000",1,"Add AMPure beads to sample and Mixing",0,1,0);

Aspirate(255,"Clearing BEAD contents in TIP","20","20","20","20","20","20","20","20",0,0,0,0,21,0,1,"0C08ｯ1000000000000",1,"Add AMPure beads to sample and Mixing",0,1,0);

Dispense(255,"Clearing BEAD contents in TIP Zmax -17","20","20","20","20","20","20","20","20",0,0,0,0,21,0,1,"0C08ｯ1000000000000",1,"Add AMPure beads to sample and Mixing",0,1,0);

Set_DITIs_Back(255,15,1,"0C08ｯ1000000000000",1,"Add AMPure beads to sample and Mixing",0,1,0);

EndLoop();

Comment("Remove script of 'Mix the AMPure beads and Sample 3 loops ' 24th Feb Kojima");

StartTimer("1");

WaitTimer("1","30 * 60");

Comment(" Move RT Assay Area 1 --> Magnet ( wait 10 mins )");

Vector("RT Assay 1 <> SAFE","21","1",0,1,0,1,1,0);

Vector("SAFE <> Magnet","21","2",0,1,2,0,1,0);

Vector("SAFE <> PUSH down Magnet","21","2",0,1,0,2,1,0);

Vector("Roma FAR RIGHT","35","5",0,1,2,2,1,0);

Comment("Wait 10 mins on magnet ");

StartTimer("1");

WaitTimer("1","10 * 60");

Comment("REMOVE Liquid from MAGNET plate ADD EtOH and REMOVE");

Group("Remove supernatant 150ul and Add 100 percent EtOH");

Vector("ET 2 LID <> SAFE","31","3",0,1,0,1,1,0);

BeginLoop("NC","Remove supernatant and Add 100 percent EtOH");

Wash(255,1,1,1,0,"2",500,"1",500,10,70,30,1,0,1000,0);

Subroutine("C:¥Program Files¥TECAN¥EVOware¥database¥Scripts¥Diluter_Aspirate_Air.esc",0);

PickUp_DITIs2(255,15,1,"0C08ｯ1000000000000",1,"Remove supernatant and Add 100 percent EtOH",0,1,"DiTi 200 ul MIX2",0);

MoveLiha(255,15,1,1,"0C08ｯ1000000000000",4,3,25,400,1,"Remove supernatant and Add 100 percent EtOH",0,1,0);

Subroutine("C:¥Program Files¥TECAN¥EVOware¥database¥Scripts¥Diluter_Dispense_Air.esc",0);

Aspirate(255,"Remove Supernatant","150","150","150","150","150","150","150","150",0,0,0,0,21,1,1,"0C08ｯ1000000000000",1,"Remove supernatant and Add 100 percent EtOH",0,1,0);

Dispense(255,"Clearing contents of TIP","150","150","150","150","150","150","150","150",0,0,0,0,1,1,1,"0108ｯ1",0,0);

Set_DITIs_Back(255,15,1,"0C08ｯ1000000000000",1,"Remove supernatant and Add 100 percent EtOH",0,1,0);

Wash(255,1,1,1,0,"2",500,"1",500,10,70,30,1,0,1000,0);

PickUp_DITIs2(255,2,1,"0C08ｯ1000000000000",0,"DiTi 200 ul EtOH USE",0);

Mix(255,"Solvent Mix","145","145","145","145","145","145","145","145",0,0,0,0,31,2,1,"0108ｯ1",2,0,0);

Aspirate(255,"Solvent for EtOH trough","140","140","140","140","140","140","140","140",0,0,0,0,31,2,1,"0108ｯ1",0,0);

Dispense(255,"Solvent","140","140","140","140","140","140","140","140",0,0,0,0,21,1,1,"0C08ｯ1000000000000",1,"Remove supernatant and Add 100 percent EtOH",0,1,0);

Set_DITIs_Back(255,2,1,"0C08ｯ1000000000000",0,0);

EndLoop();

Vector("ET 2 LID <> SAFE","31","3",0,1,2,0,1,0);

GroupEnd();

Vector("ET 1 LID <> SAFE","28","3",0,1,0,1,1,0);

Wash(255,1,1,1,0,"2",500,"1",500,10,70,30,1,0,1000,0);

BeginLoop("NC","Wash the AMPure beads and Add water for sample elution");

Comment("Remove supernatant of 100 percent EtOH");

Subroutine("C:¥Program Files¥TECAN¥EVOware¥database¥Scripts¥Diluter_Aspirate_Air.esc",0);

PickUp_DITIs2(255,15,1,"0C08ｯ1000000000000",1,"Wash the AMPure beads and Add water for sample elution",0,1,"DiTi 200 ul MIX2",0);

MoveLiha(255,15,1,1,"0C08ｯ1000000000000",4,3,25,400,1,"Wash the AMPure beads and Add water for sample elution",0,1,0);

Subroutine("C:¥Program Files¥TECAN¥EVOware¥database¥Scripts¥Diluter_Dispense_Air.esc",0);

Aspirate(255,"Aspirate EtOH Low Volume Liquid Level","70","70","70","70","70","70","70","70",0,0,0,0,21,1,1,"0C08ｯ1000000000000",1,"Wash the AMPure beads and Add water for sample elution",0,1,0);

Dispense(255,"Clearing contents of TIP","70","70","70","70","70","70","70","70",0,0,0,0,1,1,1,"0108ｯ1",0,0);

Aspirate(255,"Remove Excess liquid from Magnet Plate","110","110","110","110","110","110","110","110",0,0,0,0,21,1,1,"0C08ｯ1000000000000",1,"Wash the AMPure beads and Add water for sample elution",0,1,0);

Dispense(255,"Clearing contents of TIP","110","110","110","110","110","110","110","110",0,0,0,0,1,1,1,"0108ｯ1",0,0);

Set_DITIs_Back(255,15,1,"0C08ｯ1000000000000",1,"Wash the AMPure beads and Add water for sample elution",0,1,0);

Comment("Add 70 percent EtOH 200ul 1st");

Wash(255,1,1,1,0,"2",500,"1",500,10,70,30,1,0,1000,0);

PickUp_DITIs2(255,2,1,"0C08ｯ1000000000000",0,"DiTi 200 ul EtOH USE",0);

Mix(255,"Solvent Mix","100","100","100","100","100","100","100","100",0,0,0,0,28,2,1,"0108ｯ1",2,0,0);

Aspirate(255,"Solvent for EtOH trough","100","100","100","100","100","100","100","100",0,0,0,0,28,2,1,"0108ｯ1",0,0);

Dispense(255,"Solvent","100","100","100","100","100","100","100","100",0,0,0,0,21,1,1,"0C08ｯ1000000000000",1,"Wash the AMPure beads and Add water for sample elution",0,1,0);

Aspirate(255,"Solvent for EtOH trough","100","100","100","100","100","100","100","100",0,0,0,0,28,2,1,"0108ｯ1",0,0);

Dispense(255,"Solvent","100","100","100","100","100","100","100","100",0,0,0,0,21,1,1,"0C08ｯ1000000000000",1,"Wash the AMPure beads and Add water for sample elution",0,1,0);

Detect_Liquid(255,"Solvent",28,2,1,"0108ｯ1",0,0);

Set_DITIs_Back(255,2,1,"0C08ｯ1000000000000",0,0);

Comment("Remove supernatant of 70 percent EtOH 1st");

Wash(255,1,1,1,0,"2",500,"1",500,10,70,30,1,0,1000,0);

Subroutine("C:¥Program Files¥TECAN¥EVOware¥database¥Scripts¥Diluter_Aspirate_Air.esc",0);

PickUp_DITIs2(255,15,1,"0C08ｯ1000000000000",1,"Wash the AMPure beads and Add water for sample elution",0,1,"DiTi 200 ul MIX2",0);

MoveLiha(255,15,1,1,"0C08ｯ1000000000000",4,3,25,400,1,"Wash the AMPure beads and Add water for sample elution",0,1,0);

Subroutine("C:¥Program Files¥TECAN¥EVOware¥database¥Scripts¥Diluter_Dispense_Air.esc",0);

Aspirate(255,"Aspirate EtOH Low Volume Liquid Level","120","120","120","120","120","120","120","120",0,0,0,0,21,1,1,"0C08ｯ1000000000000",1,"Wash the AMPure beads and Add water for sample elution",0,1,0);

Dispense(255,"Clearing contents of TIP","120","120","120","120","120","120","120","120",0,0,0,0,1,1,1,"0108ｯ1",0,0);

Aspirate(255,"Solvent without asp airgap at end","130","130","130","130","130","130","130","130",0,0,0,0,21,1,1,"0C08ｯ1000000000000",1,"Wash the AMPure beads and Add water for sample elution",0,1,0);

Dispense(255,"Clearing contents of TIP","130","130","130","130","130","130","130","130",0,0,0,0,1,1,1,"0108ｯ1",0,0);

Set_DITIs_Back(255,15,1,"0C08ｯ1000000000000",1,"Wash the AMPure beads and Add water for sample elution",0,1,0);

Comment("Add 70 percent EtOH 200ul 2nd");

Wash(255,1,1,1,0,"2",500,"1",500,10,70,30,1,0,1000,0);

PickUp_DITIs2(255,2,1,"0C08ｯ1000000000000",0,"DiTi 200 ul EtOH USE",0);

Aspirate(255,"Solvent for EtOH trough","100","100","100","100","100","100","100","100",0,0,0,0,28,2,1,"0108ｯ1",0,0);

Dispense(255,"Solvent","100","100","100","100","100","100","100","100",0,0,0,0,21,1,1,"0C08ｯ1000000000000",1,"Wash the AMPure beads and Add water for sample elution",0,1,0);

Aspirate(255,"Solvent for EtOH trough","100","100","100","100","100","100","100","100",0,0,0,0,28,2,1,"0108ｯ1",0,0);

Dispense(255,"Solvent","100","100","100","100","100","100","100","100",0,0,0,0,21,1,1,"0C08ｯ1000000000000",1,"Wash the AMPure beads and Add water for sample elution",0,1,0);

Detect_Liquid(255,"Solvent",28,2,1,"0108ｯ1",0,0);

Set_DITIs_Back(255,2,1,"0C08ｯ1000000000000",0,0);

Comment("Remove supernatant of 70 percent EtOH 2nd");

Wash(255,1,1,1,0,"5",500,"4.0",500,10,70,30,1,0,1000,0);

Subroutine("C:¥Program Files¥TECAN¥EVOware¥database¥Scripts¥Diluter_Aspirate_Air.esc",0);

PickUp_DITIs2(255,15,1,"0C08ｯ1000000000000",1,"Wash the AMPure beads and Add water for sample elution",0,1,"DiTi 200 ul MIX2",0);

MoveLiha(255,15,1,1,"0C08ｯ1000000000000",4,3,25,400,1,"Wash the AMPure beads and Add water for sample elution",0,1,0);

Subroutine("C:¥Program Files¥TECAN¥EVOware¥database¥Scripts¥Diluter_Dispense_Air.esc",0);

Aspirate(255,"Aspirate EtOH Low Volume Liquid Level","120","120","120","120","120","120","120","120",0,0,0,0,21,1,1,"0C08ｯ1000000000000",1,"Wash the AMPure beads and Add water for sample elution",0,1,0);

Dispense(255,"Clearing contents of TIP","120","120","120","120","120","120","120","120",0,0,0,0,1,1,1,"0108ｯ1",0,0);

Aspirate(255,"Solvent without asp airgap at end","70","70","70","70","70","70","70","70",0,0,0,0,21,1,1,"0C08ｯ1000000000000",1,"Wash the AMPure beads and Add water for sample elution",0,1,0);

Dispense(255,"Clearing contents of TIP","70","70","70","70","70","70","70","70",0,0,0,0,1,1,1,"0108ｯ1",0,0);

Aspirate(255,"Solvent","70","70","70","70","70","70","70","70",0,0,0,0,21,1,1,"0C08ｯ1000000000000",1,"Wash the AMPure beads and Add water for sample elution",0,1,0);

Dispense(255,"Clearing contents of TIP","70","70","70","70","70","70","70","70",0,0,0,0,1,1,1,"0108ｯ1",0,0);

Aspirate(255,"Clearing contents of TIP","100","100","100","100","100","100","100","100",0,0,0,0,1,1,1,"0108ｯ1",0,0);

Dispense(255,"Clearing contents of TIP variation","100","100","100","100","100","100","100","100",0,0,0,0,1,1,1,"0108ｯ1",0,0);

Set_DITIs_Back(255,15,1,"0C08ｯ1000000000000",1,"Wash the AMPure beads and Add water for sample elution",0,1,0);

Comment("Add water 42ul for sample elution");

Wash(255,1,1,1,0,"10",500,"4.0",500,10,70,30,1,0,1000,0);

PickUp_DITIs2(255,2,1,"0C0800ｬ70000000000",0,"DiTi 200 ul EtOH USE",0);

Aspirate(255,"SD Water free dispense","42","42","42","42","42","42","42","42",0,0,0,0,35,0,1,"0108ｯ1",0,0);

Dispense(255,"SD Water free dispense","42","42","42","42","42","42","42","42",0,0,0,0,21,1,1,"0C08ｯ1000000000000",1,"Wash the AMPure beads and Add water for sample elution",0,1,0);

Set_DITIs_Back(255,2,1,"0C0800ｬ70000000000",0,0);

EndLoop();

Wash(255,1,1,1,0,"2",500,"1",500,10,70,30,1,0,1000,0);

Vector("ET 1 LID <> SAFE","28","3",0,1,2,0,1,0);

Comment("Move from Magnet --> to SHIFT position");

Vector("SAFE <> Magnet","21","2",0,1,0,1,1,0);

Vector("MAGNET SHIFTING","21","2",0,0,2,2,1,0);

StartTimer("1");

WaitTimer("1","60");

Vector("MAGNET SHIFTING","21","2",1,0,2,2,1,0);

Vector("RT Assay 1 <> SAFE","21","1",0,1,2,0,1,0);

Vector("RT Assay 1 <> PUSHdown","21","1",0,1,0,0,1,0);

Comment("REPLACE DITI BOX 1");

Vector("Transfer Diti 200 @ Cooling Carrier","8","1",0,1,0,1,1,0);

Vector("Transfer Diti Slot A","10","1",0,1,2,0,1,0);

Vector("Transfer Diti Slot B","10","1",0,1,0,1,1,0);

Vector("Transfer Diti 200 @ Cooling Carrier","8","1",0,1,2,0,1,0);

Vector("Transfer Diti 200 @ Cooling Carrier","8","2",0,1,0,1,1,0);

Vector("Transfer Diti Slot A","10","1",0,1,2,0,1,0);

Vector("Transfer Diti Slot B","10","1",0,1,0,1,1,0);

Vector("Transfer Diti 200 @ Cooling Carrier","8","2",0,1,2,0,1,0);

Set_DITI_Counter2("DiTi 200 ul","8","1","1",0);

ROMA(2,80,75,0,0,0,150,1,0);

Comment("END REPLACE DITI BOX ");

Comment("Mix the sample 42ul on RT");

Notification(1,"EVO Team","Confirmation:Plate stand: EVO 1st Information","Check Plate! About 30 min before",0);

BeginLoop("NC","Mix the sample for elution");

Wash(255,1,1,1,0,"2",500,"1",500,10,70,30,1,0,1000,0);

GetDITI2(255,"DiTi 200 ul",1,0,10,70);

Aspirate(255,"Mixing Beads with Sample small volume aspirate","5","5","5","5","5","5","5","5",0,0,0,0,21,0,1,"0C08ｯ1000000000000",1,"Mix the sample for elution",0,1,0);

IfThen("TEST_MODE",0,"0");

Variable(D,"5",0,"",0,0.000000,0.000000,0,2,0,0);

Else();

Variable(D,"30",0,"",0,0.000000,0.000000,0,2,0,0);

EndIf();

BeginLoop("D","Sample mixing");

Aspirate(255,"Mixing Beads with Sample small fast","25","25","25","25","25","25","25","25",0,0,0,0,21,0,1,"0C08ｯ1000000000000",1,"Mix the sample for elution",0,1,0);

Dispense(255,"Mixing Beads with Sample small fast","25","25","25","25","25","25","25","25",0,0,0,0,21,0,1,"0C08ｯ1000000000000",1,"Mix the sample for elution",0,1,0);

EndLoop();

Dispense(255,"Mixing Beads with Sample small volume aspirate","5","5","5","5","5","5","5","5",0,0,0,0,21,0,1,"0C08ｯ1000000000000",1,"Mix the sample for elution",0,1,0);

Detect_Liquid(255,"Mixing Beads with Sample small fast",21,0,1,"0C08ｯ1000000000000",1,"Mix the sample for elution",0,1,0);

Aspirate(255,"Clearing BEAD contents in TIP","20","20","20","20","20","20","20","20",0,0,0,0,21,0,1,"0C08ｯ1000000000000",1,"Mix the sample for elution",0,1,0);

Dispense(255,"Clearing BEAD contents in TIP_Z-max -9","20","20","20","20","20","20","20","20",0,0,0,0,21,0,1,"0C08ｯ1000000000000",1,"Mix the sample for elution",0,1,0);

DropDITI(255,2,2,10,70,0);

EndLoop();

Wash(255,1,1,1,0,"2",500,"1",500,10,70,30,1,0,1000,0);

FACTS("ThermalCycler","ThermalCycler_OpenLid","1","0","");

Vector("RT Assay 1 <> SAFE","21","1",0,1,0,1,1,0);

Vector("SAFE <> Cycler","45","1",0,1,2,0,1,0);

FACTS("ThermalCycler","ThermalCycler_CloseLid","1,0","0","");

Comment("10.25 Stand(Position) was changed");

Notification(1,"EVO Team","Confirmation:Plate stand: EVO 1st Information","Check Plate!",0);

Comment("Supply New 96 plate");

Vector("ExtractedLiquid <> SAFE","35","5",0,1,0,1,1,0);

Vector("RT Assay 1 <> SAFE","21","1",0,1,2,0,1,0);

Vector("RT Assay 1 <> PUSHdown","21","1",0,1,0,0,1,0);

ROMA(2,80,75,0,0,0,150,1,0);

Comment("10.25 Stand(Position) was changed");

IfThen("TEST_MODE",0,"0");

StartTimer("1");

WaitTimer("1","10");

Else();

StartTimer("1");

FACTS("ThermalCycler","ThermalCycler_RunBlock","1,SD42,BLOCK,ON,300","0","");

WaitTimer("1","315");

EndIf();

FACTS("ThermalCycler","ThermalCycler_OpenLid","1","0","");

Vector("SAFE <> Cycler","45","1",0,1,0,1,1,0);

Vector("SAFE <> Magnet","21","2",0,1,2,0,1,0);

Vector("SAFE <> PUSH down Magnet","21","2",0,1,0,2,1,0);

Comment("Move RT Assay Area 1 --> Magnet (wait 10 mins)");

Vector("Roma FAR RIGHT","35","5",0,1,2,2,0,0);

StartTimer("1");

Comment("END REPLACE DITI BOX 2");

WaitTimer("1","10* 60");

Comment("Remove -4");

Comment("Change Aspirate speed (2ul/s) Left-over 24th Mar Kojima");

BeginLoop("NC","Transfer the sample to new plate");

Wash(255,1,1,1,0,"2",500,"1",500,10,70,30,1,0,1000,0);

GetDITI2(255,"DiTi 200 ul",0,0,10,70);

Aspirate(255,"Left-over","47","47","47","47","47","47","47","47",0,0,0,0,21,1,1,"0C08ｯ1000000000000",1,"Transfer the sample to new plate",0,1,0);

Dispense(255,"AMpure Collection","47","47","47","47","47","47","47","47",0,0,0,0,21,0,1,"0C08ｯ1000000000000",1,"Transfer the sample to new plate",0,1,0);

DropDITI(255,2,2,10,70,0);

EndLoop();

Comment("10.25 Stand(Position) was changed");

Vector("RT Assay 1 <> SAFE","21","1",0,1,0,1,1,0);

Vector("ExtractedLiquid <> SAFE","35","5",0,1,2,0,1,0);

Vector("ExtractedLiquid <> PUSHdown","35","5",0,1,0,0,1,0);

ROMA(2,80,75,0,0,0,150,1,0);

Comment("10.25 Stand(Position) was changed");

Comment("Incubate for 10min on 37 degree for remove contaminated EtOH");

StartTimer("1");

WaitTimer("1","10 * 60");

Vector("ExtractedLiquid <> SAFE","35","5",0,1,0,1,1,0);

Vector("FourDegreesPlate <-> SAFE","8","4",0,1,2,0,1,0);

ROMA(2,80,75,0,0,0,150,1,0);

If("1",0,"1","SKIP");

Comment("Dispose Obsolete plate from Magnet");

Vector("SAFE <> Magnet","21","2",0,1,0,1,1,0);

Vector("Dispose OLD Magnet Plate","21","2",0,1,2,0,1,0);

Comment("REPLACE DITI BOX 3");

Vector("Transfer Diti 200 @ Cooling Carrier","8","1",0,1,0,1,1,0);

Vector("Transfer Diti Slot B","10","1",0,1,2,0,1,0);

Vector("Transfer Diti Slot C","10","1",0,1,0,1,1,0);

Vector("Transfer Diti 200 @ Cooling Carrier","8","1",0,1,2,0,1,0);

Vector("Transfer Diti 200 @ Cooling Carrier","15","1",0,1,0,1,1,0);

Vector("Transfer Diti Slot C","10","1",0,1,2,0,1,0);

Vector("Transfer Diti 200 @ Cooling Carrier","15","3",0,1,0,1,1,0);

Vector("Transfer Diti Slot C","10","1",0,1,2,0,1,0);

Vector("Transfer Diti Slot D","10","1",0,1,0,1,1,0);

Vector("Transfer Diti 200 @ Cooling Carrier","15","1",0,1,2,0,1,0);

Vector("Transfer Diti Slot D","10","1",0,1,0,1,1,0);

Vector("Transfer Diti 200 @ Cooling Carrier","15","3",0,1,2,0,1,0);

Comment("SKIP");

ROMA(2,80,75,0,0,0,150,1,0);

GroupEnd();

GroupEnd();

Comment("A");

Notification(0,"EVO Team","Assay finish","Assay finish",0);

**---END---**

**3. BIOTINYLATION_and_PURIFICATION**

**---START---**

40CD1E2B

20110601_152421 Admin

Administrator

--{ RES }--

V;200

--{ CFG }--

999;219;32;

14;-1;30;8;-1;-1;-1;-1;-1;234;-1;-1;-1;-1;-1;-1;96;-1;-1;-1;-1;-1;34;-1;-1;-1;-1;-1;246;244;245;243;242;238;-1;-1;239;-1;-1;-1;-1;-1;-1;-1;-1;-1;-1;-1;-1;-1;-1;-1;-1;-1;-1;-1;-1;-1;-1;-1;-1;-1;-1;-1;-1;-1;-1;-1;-1;-1;-1;-1;-1;-1;-1;-1;-1;-1;-1;-1;-1;-1;-1;-1;-1;-1;-1;-1;-1;-1;-1;-1;-1;-1;-1;-1;-1;-1;-1;-1;

998;0;

998;3;Wash Station Cleaner shallow;Wash Station Waste;Wash Station Cleaner deep;

998;;;;

998;3;;DiTi 200 ul EtOH USE;DiTi Waste;

998;;Diti 200- EtOH USE;Waste;

998;0;

998;0;

998;0;

998;0;

998;0;

998;4;DiTi 200 ul;DiTi 200 ul;DiTi 50ul 2;96 Well PCR Plate;

998;Diti 200 -A;Diti 200 -B;DiTi 50ul;Cold PCR plate;

998;0;

998;0;

998;0;

998;0;

998;0;

998;0;

998;3;;;DiTi 200 ul MIX3;

998;;;Diti AMP and CAPT 4;

998;0;

998;0;

998;0;

998;0;

998;0;

998;3;96 Well RT PCR;96 Well MAGNET PCR;;

998;RT PCR plate;MAGNET PCR;;

998;0;

998;0;

998;0;

998;0;

998;0;

998;3;;Tube 8pos RT Amp2;;

998;;A3;;

998;3;;;Trough 100ml;

998;;;E70;

998;3;;;;

998;;;;

998;3;0.5 ml Eppendorf Tube BIO;Trough 100ml ISO;;

998;Bio;Iso;;

998;3;;;Trough 100ml ET 2;

998;;;E100;

998;6;;;;;;;

998;;;;;;;

998;0;

998;0;

998;7;Trough 25ml SD;;;;96 Well Microplate HOT;;;

998;SD;;;;Extd Liquid 3;;;

998;0;

998;0;

998;0;

998;0;

998;0;

998;0;

998;0;

998;0;

998;0;

998;0;

998;0;

998;0;

998;0;

998;0;

998;0;

998;0;

998;0;

998;0;

998;0;

998;0;

998;0;

998;0;

998;0;

998;0;

998;0;

998;0;

998;0;

998;0;

998;0;

998;0;

998;0;

998;0;

998;0;

998;0;

998;0;

998;0;

998;0;

998;0;

998;0;

998;0;

998;0;

998;0;

998;0;

998;0;

998;0;

998;0;

998;0;

998;0;

998;0;

998;0;

998;0;

998;0;

998;0;

998;0;

998;0;

998;0;

998;0;

998;0;

998;0;

998;0;

998;0;

998;0;

998;0;

998;2;

998;86;41;

998;241;10;

998;5;

998;4;0;System;

998;0;3;Magellan;

998;0;0;Thermal Cycler;

998;0;1;Hotel 4Pos DeepWell;

998;0;2;Hotel 2Pos DitiRack 1;

998;3;

998;236;96 Well PCR Plate;

998;86;96 Well PCR Plate;

998;241;DiTi 200 ul;

998;1;

998;1;

998;45;

998;41;

998;10;

996;0;0;

--{ RPG }--

Notification(1,"EVO Team","Error: EVO 1st Information","Error",1);

Comment("Mixing step of Enzyme was changed.Oct 19th Tera");

Comment("50ul Diti has been applied, Oct 7th 2010 Tera");

Comment("***Start Position : RT Rack***");

Comment("Change name of loop 18th Feb Kojima");

Comment("Adjust the program of AMPure 18th Feb Kojima");

Comment("Renew the Script of AMPure mixing 1st Mar Kojima");

Comment("Change name of loop 2nd Mar Kojima");

Variable(TEST_MODE,"1",0,"TEST=0, REAL SUMPLE=1",0,0.000000,1.000000,0,2,0,0);

Variable(NC,"12",0,"何列分処理しますか？",0,1.000000,12.000000,0,2,0,0);

Set_DITI_Counter2("DiTi 200 ul","8","1","1",0);

Set_DITI_Counter2("DiTi 50ul 2","8","3","1",0);

Command("C5SSP800,90,0",1,1,,,2,2,0);

ROMA(2,80,75,0,0,0,150,1,0);

Comment("START < Section A >");

Group("(3) Biotinylation");

Comment("Biotinylation mix 8uL");

Vector("Bio Lid <> SAFE","30","1",0,1,0,1,1,0);

Comment("Modified 10.19.2010");

BeginLoop("NC","Add Biotinylation reagent to sample ");

Wash(255,1,1,1,0,"2",500,"1",500,10,70,30,1,0,1000,0);

GetDITI2(255,"DiTi 50ul 2",0,0,10,70);

Aspirate(255,"Clearing BEAD contents in TIP","5","5","5","5","5","5","5","5",0,0,0,0,30,0,1,"0108ｯ1",0,0);

Mix(255,"Enzyme Liquid Level","12","12","12","12","12","12","12","12",0,0,0,0,30,0,1,"0108ｯ1",2,0,0);

Aspirate(255,"BIOtin wet contact dispense at Liquid Level2","8","8","8","8","8","8","8","8",0,0,0,0,30,0,1,"0108ｯ1",0,0);

Dispense(255,"BIOtin wet contact dispense at Liquid Level2","8","8","8","8","8","8","8","8",0,0,0,0,21,0,1,"0C08ｯ1000000000000",1,"Add Biotinylation reagent to sample ",0,1,0);

Aspirate(255,"Aspirate Low Volume further lower Liquid Level2","3","3","3","3","3","3","3","3",0,0,0,0,21,0,1,"0C08ｯ1000000000000",1,"Add Biotinylation reagent to sample ",0,1,0);

BeginLoop("5","Biotinylation sample mixing");

Aspirate(255,"Mixing BIO inside Plate Slower2","34","34","34","34","34","34","34","34",0,0,0,0,21,0,1,"0C08ｯ1000000000000",1,"Add Biotinylation reagent to sample ",0,1,0);

Dispense(255,"Mixing BIO inside Plate Dispense Top2","34","34","34","34","34","34","34","34",0,0,0,0,21,0,1,"0C08ｯ1000000000000",1,"Add Biotinylation reagent to sample ",0,1,0);

EndLoop();

Aspirate(255,"Mixing BIO inside Plate Slower2","34","34","34","34","34","34","34","34",0,0,0,0,21,0,1,"0C08ｯ1000000000000",1,"Add Biotinylation reagent to sample ",0,1,0);

Dispense(255,"Mixing BIO inside Plate Dispense Top3","34","34","34","34","34","34","34","34",0,0,0,0,21,0,1,"0C08ｯ1000000000000",1,"Add Biotinylation reagent to sample ",0,1,0);

Dispense(255,"Aspirate Low Volume further lower Liquid Level2","3","3","3","3","3","3","3","3",0,0,0,0,21,0,1,"0C08ｯ1000000000000",1,"Add Biotinylation reagent to sample ",0,1,0);

StartTimer("1");

WaitTimer("1","2");

Dispense(255,"Blow out slow","5","5","5","5","5","5","5","5",0,0,0,0,21,0,1,"0C08ｯ1000000000000",1,"Add Biotinylation reagent to sample ",0,1,0);

Aspirate(255,"Clearing BEAD contents in TIP","25","25","25","25","25","25","25","25",0,0,0,0,21,0,1,"0C08ｯ1000000000000",1,"Add Biotinylation reagent to sample ",0,1,0);

Dispense(255,"Clearing contents of TIP variation","25","25","25","25","25","25","25","25",0,0,0,0,21,0,1,"0C08ｯ1000000000000",1,"Add Biotinylation reagent to sample ",0,1,0);

Detect_Liquid(255,"Mixing Beads with Sample small fast",21,0,1,"0C08ｯ1000000000000",1,"Add Biotinylation reagent to sample ",0,1,0);

DropDITI(255,2,2,10,70,0);

Comment("End Modified 10.19.2010");

EndLoop();

Vector("Bio Lid <> SAFE","30","1",0,1,2,0,1,0);

FACTS("ThermalCycler","ThermalCycler_OpenLid","1","0","");

Comment("Move RT Plate --> T.Cycler 37 degrees ( wait 2 hours in Darkness )");

Vector("RT Assay 1 <> SAFE","21","1",0,1,0,1,1,0);

Vector("SAFE <> Cycler","45","1",0,1,2,0,1,0);

Vector("SAFE <> Cycler","45","1",0,0,0,1,1,0);

Vector("SAFE <> Cycler","45","1",1,0,0,2,1,0);

FACTS("ThermalCycler","ThermalCycler_CloseLid","1,0","0","");

IfThen("TEST_MODE",0,"0");

StartTimer("1");

WaitTimer("1","10");

Else();

StartTimer("1");

FACTS("ThermalCycler","ThermalCycler_RunBlock","1,BIO,BLOCK,ON,7200","0","");

WaitTimer("1","7230");

EndIf();

FACTS("ThermalCycler","ThermalCycler_OpenLid","1","0","");

Comment("Move T.Cycler --> RT Assay Area 1");

Vector("SAFE <> Cycler","45","1",0,1,0,1,1,0);

Vector("RT Assay 1 <> SAFE","21","1",0,1,2,0,1,0);

Vector("RT Assay 1 <> PUSHdown","21","1",0,1,0,0,1,0);

GroupEnd();

Group("Add Isopropanol to sample");

Comment("Isopropanol 12uL");

Comment("Change mix speed (120ul/s --> 20ul/s) 25th Mar Kojima");

Vector("Iso LID <> SAFE","30","2",0,1,0,1,1,0);

BeginLoop("NC","Add Isopropanol to sample");

Wash(255,1,1,1,0,"2",500,"1",500,10,70,30,1,0,1000,0);

GetDITI2(255,"DiTi 200 ul",1,0,10,70);

Mix(255,"Solvent Mix","20","20","20","20","20","20","20","20",0,0,0,0,30,1,1,"0108ｯ1",2,0,0);

Aspirate(255,"ISO wet contact dispense","12","12","12","12","12","12","12","12",0,0,0,0,30,1,1,"0108ｯ1",0,0);

Dispense(255,"Solvent ISO","12","12","12","12","12","12","12","12",0,0,0,0,21,0,1,"0C08ｯ1000000000000",1,"Add Isopropanol to sample",0,1,0);

Aspirate(255,"Aspirate Low Volume further lower Liquid Level","3","3","3","3","3","3","3","3",0,0,0,0,21,0,1,"0C08ｯ1000000000000",1,"Add Isopropanol to sample",0,1,0);

Mix(255,"Mixing Enzyme inside Plate","35","35","35","35","35","35","35","35",0,0,0,0,21,0,1,"0C08ｯ1000000000000",5,1,"Add Isopropanol to sample",0,1,0);

Dispense(255,"Low Volume wet contact dispense at Liquid Level","3","3","3","3","3","3","3","3",0,0,0,0,21,0,1,"0C08ｯ1000000000000",1,"Add Isopropanol to sample",0,1,0);

Detect_Liquid(255,"Mixing Beads with Sample small fast",21,0,1,"0C08ｯ1000000000000",1,"Add Isopropanol to sample",0,1,0);

DropDITI(255,2,2,10,70,0);

EndLoop();

Vector("Iso LID <> SAFE","30","2",0,1,2,0,1,0);

Comment("REPLACE DITI BOX 4");

Comment("Renew the Script of AMPure mixing 1st Mar Kojima");

Group("(3) AMpure purification");

Wash(255,1,1,1,0,"2",500,"1",500,10,70,30,1,0,1000,0);

PickUp_DITIs2(255,2,1,"0C08000000000ｮ3000",0,"DiTi 200 ul EtOH USE",0);

Aspirate(255,"Aspirate Low Volume Liquid Level","5","5","5","5","5","5","5","5",0,0,0,0,27,1,1,"0108ｯ1",0,0);

BeginLoop("10","AMPure beads mixing");

Aspirate(255,"Mixing for AMpure in Trough close to Z-max","180","180","180","180","180","180","180","180",0,0,0,0,27,1,1,"0108ｯ1",0,0);

Dispense(255,"Mixing AMPure dropplets high position","180","180","180","180","180","180","180","180",0,0,0,0,27,1,1,"0108ｯ1",0,0);

Mix(255,"Mixing for AMpure in Trough close to Z-max","150","150","150","150","150","150","150","150",0,0,0,0,27,1,1,"0108ｯ1",3,0,0);

EndLoop();

Dispense(255,"Low Volume wet contact dispense with tracking HIGHER Liq Positn","5","5","5","5","5","5","5","5",0,0,0,0,27,1,1,"0108ｯ1",0,0);

Detect_Liquid(255,"Contact dispense with tracking HIGH Liq Positn",27,1,1,"0108ｯ1",0,0);

DropDITI(255,2,2,10,70,0);

BeginLoop("NC","Add AMPure beads to sample and mixng");

Wash(255,1,1,1,0,"2",500,"1",500,10,70,30,1,0,1000,0);

Subroutine("C:¥Program Files¥TECAN¥EVOware¥database¥Scripts¥Diluter_Aspirate_Air.esc",0);

PickUp_DITIs2(255,15,2,"0C08ｯ1000000000000",1,"Add AMPure beads to sample and mixng",0,1,"DiTi 200 ul MIX3",0);

MoveLiha(255,15,2,1,"0C08ｯ1000000000000",4,3,25,400,1,"Add AMPure beads to sample and mixng",0,1,0);

Subroutine("C:¥Program Files¥TECAN¥EVOware¥database¥Scripts¥Diluter_Dispense_Air.esc",0);

BeginLoop("3","AMPure beads mixing");

Aspirate(255,"Mixing for AMpure in Trough close to Z-max","80","80","80","80","80","80","80","80",0,0,0,0,27,1,1,"0108ｯ1",0,0);

Dispense(255,"Mixing AMPure dropplets high position","80","80","80","80","80","80","80","80",0,0,0,0,27,1,1,"0108ｯ1",0,0);

Mix(255,"Mixing for AMpure in Trough close to Z-max","80","80","80","80","80","80","80","80",0,0,0,0,27,1,1,"0108ｯ1",2,0,0);

EndLoop();

Aspirate(255,"AMPure and Beads","108","108","108","108","108","108","108","108",0,0,0,0,27,1,1,"0108ｯ1",0,0);

Detect_Liquid(255,"AMPure and Beads",27,1,1,"0108ｯ1",0,0);

Dispense(255,"AMPure and Beads","108","108","108","108","108","108","108","108",0,0,0,0,21,0,1,"0C08ｯ1000000000000",1,"Add AMPure beads to sample and mixng",0,1,0);

Detect_Liquid(255,"AMPure and Beads",21,0,1,"0C08ｯ1000000000000",1,"Add AMPure beads to sample and mixng",0,1,0);

Comment("Renew the Script of AMPure mixing 1st Mar Kojima");

Aspirate(255,"Aspirate 2ul Liquid Level","5","5","5","5","5","5","5","5",0,0,0,0,21,0,1,"0C08ｯ1000000000000",1,"Add AMPure beads to sample and mixng",0,1,0);

Mix(255,"Mixing Ampure inside Plate","130","130","130","130","130","130","130","130",0,0,0,0,21,0,1,"0C08ｯ1000000000000",5,1,"Add AMPure beads to sample and mixng",0,1,0);

IfThen("TEST_MODE",0,"0");

Variable(B,"3",0,"",0,0.000000,0.000000,0,2,0,0);

Else();

Variable(B,"5",0,"",0,0.000000,0.000000,0,2,0,0);

EndIf();

BeginLoop("B","AMPure beads and sample mixing");

Aspirate(255,"Mixing 3rd Ampure inside Plate Large Volume MIX","130","130","130","130","130","130","130","130",0,0,0,0,21,0,1,"0C08ｯ1000000000000",1,"Add AMPure beads to sample and mixng",0,1,0);

Dispense(255,"Mixing 3rd Ampure inside Plate Large Volume MIX","130","130","130","130","130","130","130","130",0,0,0,0,21,0,1,"0C08ｯ1000000000000",1,"Add AMPure beads to sample and mixng",0,1,0);

EndLoop();

Dispense(255,"Low Volume wet contact dispense with tracking HIGHER Liq Positn","5","5","5","5","5","5","5","5",0,0,0,0,21,0,1,"0C08ｯ1000000000000",1,"Add AMPure beads to sample and mixng",0,1,0);

Detect_Liquid(255,"Mixing Ampure inside Plate Large Volume MIX",21,0,1,"0C08ｯ1000000000000",1,"Add AMPure beads to sample and mixng",0,1,0);

Aspirate(255,"Clearing BEAD contents in TIP","20","20","20","20","20","20","20","20",0,0,0,0,21,0,1,"0C08ｯ1000000000000",1,"Add AMPure beads to sample and mixng",0,1,0);

Dispense(255,"Clearing BEAD contents in TIP Zmax -17","20","20","20","20","20","20","20","20",0,0,0,0,21,0,1,"0C08ｯ1000000000000",1,"Add AMPure beads to sample and mixng",0,1,0);

Set_DITIs_Back(255,15,2,"0C08ｯ1000000000000",1,"Add AMPure beads to sample and mixng",0,1,0);

Wash(255,1,1,1,0,"2",500,"1",500,10,70,30,1,0,1000,0);

EndLoop();

Comment("Remove script of 'Mix the AMPure beads and Sample 3 loops ' 1st Mar Kojima");

StartTimer("1");

WaitTimer("1","30 * 60");

Comment(" Move RT Assay Area 1 --> Magnet ( wait 5 mins )");

Vector("Roma FAR RIGHT","35","5",0,1,2,2,0,0);

Vector("RT Assay 1 <> SAFE","21","1",0,1,0,1,1,0);

Vector("SAFE <> Magnet","21","2",0,1,2,0,1,0);

Vector("SAFE <> PUSH down Magnet","21","2",0,1,0,2,1,0);

Vector("Roma FAR RIGHT","35","5",0,1,2,2,1,0);

Comment("Wait on Magnet for 10 mins");

StartTimer("1");

WaitTimer("1","10 * 60");

Comment("Remove supernatant and Add 100 percent EtOH");

Group("Remove supernatant and Add 100 percent EtOH");

Vector("ET 2 LID <> SAFE","31","3",0,1,0,1,1,0);

BeginLoop("NC","Remove supernatant and Add 100 percent EtOH");

Wash(255,1,1,1,0,"2",500,"1",500,10,70,30,1,0,1000,0);

Subroutine("C:¥Program Files¥TECAN¥EVOware¥database¥Scripts¥Diluter_Aspirate_Air.esc",0);

PickUp_DITIs2(255,15,2,"0C08ｯ1000000000000",1,"Remove supernatant and Add 100 percent EtOH",0,1,"DiTi 200 ul MIX3",0);

MoveLiha(255,15,2,1,"0C08ｯ1000000000000",4,3,25,400,1,"Remove supernatant and Add 100 percent EtOH",0,1,0);

Subroutine("C:¥Program Files¥TECAN¥EVOware¥database¥Scripts¥Diluter_Dispense_Air.esc",0);

Aspirate(255,"Remove Supernatant","108","108","108","108","108","108","108","108",0,0,0,0,21,1,1,"0C08ｯ1000000000000",1,"Remove supernatant and Add 100 percent EtOH",0,1,0);

Dispense(255,"Clearing contents of TIP","108","108","108","108","108","108","108","108",0,0,0,0,1,1,1,"0108ｯ1",0,0);

Set_DITIs_Back(255,15,2,"0C08ｯ1000000000000",1,"Remove supernatant and Add 100 percent EtOH",0,1,0);

Wash(255,1,1,1,0,"2",500,"1",500,10,70,30,1,0,1000,0);

PickUp_DITIs2(255,2,1,"0C08ｯ1000000000000",0,"DiTi 200 ul EtOH USE",0);

Mix(255,"Solvent Mix","145","145","145","145","145","145","145","145",0,0,0,0,31,2,1,"0108ｯ1",2,0,0);

Aspirate(255,"Solvent for EtOH trough","140","140","140","140","140","140","140","140",0,0,0,0,31,2,1,"0108ｯ1",0,0);

Dispense(255,"Solvent","140","140","140","140","140","140","140","140",0,0,0,0,21,1,1,"0C08ｯ1000000000000",1,"Remove supernatant and Add 100 percent EtOH",0,1,0);

Set_DITIs_Back(255,2,1,"0C08ｯ1000000000000",0,0);

EndLoop();

Vector("ET 2 LID <> SAFE","31","3",0,1,2,0,1,0);

GroupEnd();

Vector("ET 1 LID <> SAFE","28","3",0,1,0,1,1,0);

Wash(255,1,1,1,0,"2",500,"1",500,10,70,30,1,0,1000,0);

BeginLoop("NC","Wash the AMPure beads and Add water for sample elution");

Comment(" Remove");

Subroutine("C:¥Program Files¥TECAN¥EVOware¥database¥Scripts¥Diluter_Aspirate_Air.esc",0);

PickUp_DITIs2(255,15,2,"0C08ｯ1000000000000",1,"Wash the AMPure beads and Add water for sample elution",0,1,"DiTi 200 ul MIX3",0);

MoveLiha(255,15,2,1,"0C08ｯ1000000000000",4,3,25,400,1,"Wash the AMPure beads and Add water for sample elution",0,1,0);

Subroutine("C:¥Program Files¥TECAN¥EVOware¥database¥Scripts¥Diluter_Dispense_Air.esc",0);

Aspirate(255,"Aspirate EtOH Low Volume Liquid Level","70","70","70","70","70","70","70","70",0,0,0,0,21,1,1,"0C08ｯ1000000000000",1,"Wash the AMPure beads and Add water for sample elution",0,1,0);

Dispense(255,"Clearing contents of TIP","70","70","70","70","70","70","70","70",0,0,0,0,1,1,1,"0108ｯ1",0,0);

Aspirate(255,"Remove Excess liquid from Magnet Plate","110","110","110","110","110","110","110","110",0,0,0,0,21,1,1,"0C08ｯ1000000000000",1,"Wash the AMPure beads and Add water for sample elution",0,1,0);

Dispense(255,"Clearing contents of TIP","110","110","110","110","110","110","110","110",0,0,0,0,1,1,1,"0108ｯ1",0,0);

Set_DITIs_Back(255,15,2,"0C08ｯ1000000000000",1,"Wash the AMPure beads and Add water for sample elution",0,1,0);

Wash(255,1,1,1,0,"2",500,"1",500,10,70,30,1,0,1000,0);

Comment("EtOH 200ul -1");

Comment("Change despense retract position (2mm --> -0.4mm) 25th Mar Kojima ");

PickUp_DITIs2(255,2,1,"0C08ｯ1000000000000",0,"DiTi 200 ul EtOH USE",0);

Mix(255,"Solvent Mix","100","100","100","100","100","100","100","100",0,0,0,0,28,2,1,"0108ｯ1",2,0,0);

Aspirate(255,"Solvent for EtOH trough","100","100","100","100","100","100","100","100",0,0,0,0,28,2,1,"0108ｯ1",0,0);

Dispense(255,"Solvent","100","100","100","100","100","100","100","100",0,0,0,0,21,1,1,"0C08ｯ1000000000000",1,"Wash the AMPure beads and Add water for sample elution",0,1,0);

Aspirate(255,"Solvent for EtOH trough","100","100","100","100","100","100","100","100",0,0,0,0,28,2,1,"0108ｯ1",0,0);

Dispense(255,"Solvent","100","100","100","100","100","100","100","100",0,0,0,0,21,1,1,"0C08ｯ1000000000000",1,"Wash the AMPure beads and Add water for sample elution",0,1,0);

Detect_Liquid(255,"Mixing Ampure inside Plate Large Volume MIX",28,2,1,"0108ｯ1",0,0);

Set_DITIs_Back(255,2,1,"0C08ｯ1000000000000",0,0);

Wash(255,1,1,1,0,"2",500,"1",500,10,70,30,1,0,1000,0);

Subroutine("C:¥Program Files¥TECAN¥EVOware¥database¥Scripts¥Diluter_Aspirate_Air.esc",0);

PickUp_DITIs2(255,15,2,"0C08ｯ1000000000000",1,"Wash the AMPure beads and Add water for sample elution",0,1,"DiTi 200 ul MIX3",0);

MoveLiha(255,15,2,1,"0C08ｯ1000000000000",4,3,25,400,1,"Wash the AMPure beads and Add water for sample elution",0,1,0);

Subroutine("C:¥Program Files¥TECAN¥EVOware¥database¥Scripts¥Diluter_Dispense_Air.esc",0);

Aspirate(255,"Aspirate EtOH Low Volume Liquid Level","120","120","120","120","120","120","120","120",0,0,0,0,21,1,1,"0C08ｯ1000000000000",1,"Wash the AMPure beads and Add water for sample elution",0,1,0);

Dispense(255,"Clearing contents of TIP","120","120","120","120","120","120","120","120",0,0,0,0,1,1,1,"0108ｯ1",0,0);

Aspirate(255,"Solvent without asp airgap at end","130","130","130","130","130","130","130","130",0,0,0,0,21,1,1,"0C08ｯ1000000000000",1,"Wash the AMPure beads and Add water for sample elution",0,1,0);

Dispense(255,"Clearing contents of TIP","130","130","130","130","130","130","130","130",0,0,0,0,1,1,1,"0108ｯ1",0,0);

Set_DITIs_Back(255,15,2,"0C08ｯ1000000000000",1,"Wash the AMPure beads and Add water for sample elution",0,1,0);

Wash(255,1,1,1,0,"2",500,"1",500,10,70,30,1,0,1000,0);

Comment("Pip EtOH 2");

PickUp_DITIs2(255,2,1,"0C08ｯ1000000000000",0,"DiTi 200 ul EtOH USE",0);

Aspirate(255,"Solvent for EtOH trough","100","100","100","100","100","100","100","100",0,0,0,0,28,2,1,"0108ｯ1",0,0);

Dispense(255,"Solvent","100","100","100","100","100","100","100","100",0,0,0,0,21,1,1,"0C08ｯ1000000000000",1,"Wash the AMPure beads and Add water for sample elution",0,1,0);

Aspirate(255,"Solvent for EtOH trough","100","100","100","100","100","100","100","100",0,0,0,0,28,2,1,"0108ｯ1",0,0);

Dispense(255,"Solvent","100","100","100","100","100","100","100","100",0,0,0,0,21,1,1,"0C08ｯ1000000000000",1,"Wash the AMPure beads and Add water for sample elution",0,1,0);

Detect_Liquid(255,"Solvent",28,2,1,"0108ｯ1",0,0);

Set_DITIs_Back(255,2,1,"0C08ｯ1000000000000",0,0);

Wash(255,1,1,1,0,"2",500,"1",500,10,70,30,1,0,1000,0);

Comment("Pip Top layer 2");

Comment("Change Aspirate 70uL 2nd tip retract position (2 --> 0.5) 25th Mar Kojima");

Subroutine("C:¥Program Files¥TECAN¥EVOware¥database¥Scripts¥Diluter_Aspirate_Air.esc",0);

PickUp_DITIs2(255,15,2,"0C08ｯ1000000000000",1,"Wash the AMPure beads and Add water for sample elution",0,1,"DiTi 200 ul MIX3",0);

MoveLiha(255,15,2,1,"0C08ｯ1000000000000",4,3,25,400,1,"Wash the AMPure beads and Add water for sample elution",0,1,0);

Subroutine("C:¥Program Files¥TECAN¥EVOware¥database¥Scripts¥Diluter_Dispense_Air.esc",0);

Aspirate(255,"Aspirate EtOH Low Volume Liquid Level","120","120","120","120","120","120","120","120",0,0,0,0,21,1,1,"0C08ｯ1000000000000",1,"Wash the AMPure beads and Add water for sample elution",0,1,0);

Dispense(255,"Clearing contents of TIP","120","120","120","120","120","120","120","120",0,0,0,0,1,1,1,"0108ｯ1",0,0);

Aspirate(255,"Solvent without asp airgap at end","70","70","70","70","70","70","70","70",0,0,0,0,21,1,1,"0C08ｯ1000000000000",1,"Wash the AMPure beads and Add water for sample elution",0,1,0);

Dispense(255,"Clearing contents of TIP","70","70","70","70","70","70","70","70",0,0,0,0,1,1,1,"0108ｯ1",0,0);

Aspirate(255,"Solvent","70","70","70","70","70","70","70","70",0,0,0,0,21,1,1,"0C08ｯ1000000000000",1,"Wash the AMPure beads and Add water for sample elution",0,1,0);

Dispense(255,"Clearing contents of TIP","70","70","70","70","70","70","70","70",0,0,0,0,1,1,1,"0108ｯ1",0,0);

Aspirate(255,"Clearing contents of TIP","100","100","100","100","100","100","100","100",0,0,0,0,1,1,1,"0108ｯ1",0,0);

Dispense(255,"Clearing contents of TIP variation","100","100","100","100","100","100","100","100",0,0,0,0,1,1,1,"0108ｯ1",0,0);

Set_DITIs_Back(255,15,2,"0C08ｯ1000000000000",1,"Wash the AMPure beads and Add water for sample elution",0,1,0);

Wash(255,1,1,1,0,"2",500,"1",500,10,70,30,1,0,1000,0);

Comment("Water 42ul");

PickUp_DITIs2(255,2,1,"0C0800ｬ70000000000",0,"DiTi 200 ul EtOH USE",0);

Aspirate(255,"SD Water free dispense","42","42","42","42","42","42","42","42",0,0,0,0,35,0,1,"0108ｯ1",0,0);

Dispense(255,"SD Water free dispense","42","42","42","42","42","42","42","42",0,0,0,0,21,1,1,"0C08ｯ1000000000000",1,"Wash the AMPure beads and Add water for sample elution",0,1,0);

Set_DITIs_Back(255,2,1,"0C0800ｬ70000000000",0,0);

EndLoop();

Vector("ET 1 LID <> SAFE","28","3",0,1,2,0,1,0);

Comment("Move from Magnet --> to SHIFT position");

Vector("SAFE <> Magnet","21","2",0,1,0,1,1,0);

Vector("MAGNET SHIFTING","21","2",0,0,2,2,1,0);

StartTimer("1");

WaitTimer("1","60");

Vector("MAGNET SHIFTING","21","2",1,0,2,2,1,0);

Vector("RT Assay 1 <> SAFE","21","1",0,1,2,0,1,0);

Vector("RT Assay 1 <> PUSHdown","21","1",0,1,0,0,1,0);

ROMA(2,80,75,0,0,0,150,1,0);

Comment("Mix 42 SD on RT");

Notification(1,"EVO Team","Confirmation:Plate stand: EVO 1st Information","Check Plate! About 30 min before",0);

BeginLoop("NC","Mix the sample for elution");

Wash(255,1,1,1,0,"2",500,"1",500,10,70,30,1,0,1000,0);

GetDITI2(255,"DiTi 200 ul",1,0,10,70);

Aspirate(255,"Low Volume wet contact dispense with tracking HIGHER Liq Positn","3","3","3","3","3","3","3","3",0,0,0,0,21,0,1,"0C08ｯ1000000000000",1,"Mix the sample for elution",0,1,0);

IfThen("TEST_MODE",0,"0");

Variable(D,"5",0,"",0,0.000000,0.000000,0,2,0,0);

Else();

Variable(D,"30",0,"",0,0.000000,0.000000,0,2,0,0);

EndIf();

BeginLoop("D","Sample mixing");

Aspirate(255,"Mixing Beads with Sample small fast","25","25","25","25","25","25","25","25",0,0,0,0,21,0,1,"0C08ｯ1000000000000",1,"Mix the sample for elution",0,1,0);

Dispense(255,"Mixing Beads with Sample small fast","25","25","25","25","25","25","25","25",0,0,0,0,21,0,1,"0C08ｯ1000000000000",1,"Mix the sample for elution",0,1,0);

EndLoop();

Dispense(255,"Mixing Beads with Sample small volume aspirate","3","3","3","3","3","3","3","3",0,0,0,0,21,0,1,"0C08ｯ1000000000000",1,"Mix the sample for elution",0,1,0);

Detect_Liquid(255,"Mixing Beads with Sample small fast",21,0,1,"0C08ｯ1000000000000",1,"Mix the sample for elution",0,1,0);

Aspirate(255,"Clearing BEAD contents in TIP","20","20","20","20","20","20","20","20",0,0,0,0,21,0,1,"0C08ｯ1000000000000",1,"Mix the sample for elution",0,1,0);

Dispense(255,"Clearing BEAD contents in TIP_Z-max -9","20","20","20","20","20","20","20","20",0,0,0,0,21,0,1,"0C08ｯ1000000000000",1,"Mix the sample for elution",0,1,0);

DropDITI(255,2,2,10,70,0);

EndLoop();

Wash(255,1,1,1,0,"2",500,"1",500,10,70,30,1,0,1000,0);

FACTS("ThermalCycler","ThermalCycler_OpenLid","1","0","");

Vector("RT Assay 1 <> SAFE","21","1",0,1,0,1,1,0);

Vector("SAFE <> Cycler","45","1",0,1,2,0,1,0);

FACTS("ThermalCycler","ThermalCycler_CloseLid","1,0","0","");

Comment("10.25 Stand(Position) was changed");

Notification(1,"EVO Team","Confirmation:Plate stand: EVO 1st Information","Check Plate!",0);

Vector("ExtractedLiquid <> SAFE","35","5",0,1,0,1,1,0);

Vector("RT Assay 1 <> SAFE","21","1",0,1,2,0,1,0);

Vector("RT Assay 1 <> PUSHdown","21","1",0,1,0,0,1,0);

Vector("RT Assay 1 <> SAFE","21","1",0,0,0,1,1,0);

Vector("RT Assay 1 <> SAFE","21","1",1,0,0,2,1,0);

ROMA(2,80,75,0,0,0,150,1,0);

Comment("10.25 Stand(Position) was changed");

IfThen("TEST_MODE",0,"0");

StartTimer("1");

WaitTimer("1","10");

Else();

StartTimer("1");

FACTS("ThermalCycler","ThermalCycler_RunBlock","1,SD42,BLOCK,ON,300","0","");

WaitTimer("1","315");

EndIf();

FACTS("ThermalCycler","ThermalCycler_OpenLid","1","0","");

Vector("SAFE <> Cycler","45","1",0,1,0,1,1,0);

Vector("SAFE <> Magnet","21","2",0,1,2,0,1,0);

Vector("SAFE <> PUSH down Magnet","21","2",0,1,0,2,1,0);

Comment("Move RT Assay Area 1 --> Magnet ( wait 5 mins )");

StartTimer("1");

Comment("REPLACE DITI BOX 2 ");

Vector("Transfer Diti 200 @ Cooling Carrier","8","1",0,1,0,1,1,0);

Vector("Transfer Diti Slot A","10","1",0,1,2,0,1,0);

Vector("Transfer Diti Slot B","10","1",0,1,0,1,1,0);

Vector("Transfer Diti 200 @ Cooling Carrier","8","1",0,1,2,0,1,0);

Set_DITI_Counter2("DiTi 200 ul","8","1","1",0);

ROMA(2,80,75,0,0,0,150,1,0);

Comment("END REPLACE DITI BOX 2");

WaitTimer("1","10 * 60");

BeginLoop("NC","Transfer the sample to New plate");

Wash(255,1,1,1,0,"2",500,"1",500,10,70,30,1,0,1000,0);

GetDITI2(255,"DiTi 200 ul",0,0,10,70);

Aspirate(255,"Left-over","47","47","47","47","47","47","47","47",0,0,0,0,21,1,1,"0C08ｯ1000000000000",1,"Transfer the sample to New plate",0,1,0);

Dispense(255,"AMpure Collection","47","47","47","47","47","47","47","47",0,0,0,0,21,0,1,"0C08ｯ1000000000000",1,"Transfer the sample to New plate",0,1,0);

DropDITI(255,2,2,10,70,0);

EndLoop();

Comment("10.25 Stand(Position) was changed");

Vector("RT Assay 1 <> SAFE","21","1",0,1,0,1,1,0);

Vector("ExtractedLiquid <> SAFE","35","5",0,1,2,0,1,0);

Vector("ExtractedLiquid <> PUSHdown","35","5",0,1,0,0,1,0);

ROMA(2,80,75,0,0,0,150,1,0);

Comment("10.25 Stand(Position) was changed");

Comment("37 Incubate 10min.");

StartTimer("1");

WaitTimer("1","10 * 60");

Vector("ExtractedLiquid <> SAFE","35","5",0,1,0,1,1,0);

Vector("FourDegreesPlate <-> SAFE","8","4",0,1,2,0,1,0);

Vector("FourDegreesPlate <->PUSHdown","8","4",0,1,0,0,1,0);

If("1",0,"1","SKIP");

Vector("Transfer Diti 200 @ Cooling Carrier","8","1",0,1,0,1,1,0);

Vector("Transfer Diti Slot D","10","1",0,1,2,0,1,0);

Vector("Transfer Diti 200 @ Cooling Carrier","8","2",0,1,0,1,1,0);

Vector("Transfer Diti Slot D","10","1",0,1,2,0,1,0);

Vector("Transfer Diti 200 @ Cooling Carrier","15","2",0,1,0,1,1,0);

Vector("Transfer Diti Slot D","10","1",0,1,2,0,1,0);

ROMA(2,80,75,0,0,0,150,1,0);

Comment("Dispose Obsolete plate from Magnet");

Vector("SAFE <> Magnet","21","2",0,1,0,1,1,0);

Vector("Dispose OLD Magnet Plate","21","2",0,1,2,0,1,0);

Comment("SKIP");

ROMA(2,80,75,0,0,0,150,1,0);

GroupEnd();

GroupEnd();

Notification(0,"EVO Team","Assay finish","Assay finish",0);

**---END---**

**4.1. RNaseONE_CAPTRAPPING_REREASE**

**---START---**

71146036

20110328_111441 Admin

Administrator

--{ RES }--

V;200

--{ CFG }--

999;219;32;

14;-1;30;8;-1;-1;-1;-1;-1;234;-1;-1;-1;-1;-1;-1;96;-1;-1;-1;-1;-1;34;-1;-1;-1;-1;-1;246;244;245;243;242;238;-1;-1;239;-1;-1;-1;-1;-1;-1;-1;-1;-1;-1;-1;-1;-1;-1;-1;-1;-1;-1;-1;-1;-1;-1;-1;-1;-1;-1;-1;-1;-1;-1;-1;-1;-1;-1;-1;-1;-1;-1;-1;-1;-1;-1;-1;-1;-1;-1;-1;-1;-1;-1;-1;-1;-1;-1;-1;-1;-1;-1;-1;-1;-1;-1;-1;

998;0;

998;3;Wash Station Cleaner shallow;Wash Station Waste;Wash Station Cleaner deep;

998;;;;

998;3;;DiTi 200 ul EtOH USE;DiTi Waste;

998;;Diti 200- EtOH USE;Waste;

998;0;

998;0;

998;0;

998;0;

998;0;

998;4;DiTi 200 ul;DiTi 200 ul;DiTi 50ul 2;96 Well PCR Plate;

998;Diti 200 -A;Diti 200 -B;DiTi 50ul;Cold PCR plate;

998;0;

998;0;

998;0;

998;0;

998;0;

998;0;

998;3;DiTi 200 ul MIX1;DiTi 200 ul MIX2;DiTi 200 ul MIX3;

998;Diti AMP and CAPT 2;Diti AMP and CAPT 3;Diti AMP and CAPT 4;

998;0;

998;0;

998;0;

998;0;

998;0;

998;3;96 Well RT PCR;96 Well MAGNET PCR;;

998;RT PCR plate;MAGNET PCR;;

998;0;

998;0;

998;0;

998;0;

998;0;

998;3;;;;

998;;;;

998;3;;Trough 100ml W2;Trough 100ml ET 100percent;

998;;W2;BeadsWaste;

998;3;Trough 25ml ROB;;Trough 25ml;

998;Rob;;W4;

998;3;;;Tube 8pos MPG;

998;;;Mpg;

998;3;;;Trough 100ml ET 2;

998;;;E100;

998;6;;;0.5 ml Eppendorf Tube ROM;;;;

998;;;Rom;;;;

998;0;

998;0;

998;7;Trough 25ml SD;;96 Well Microplate HOT;;96 Well Microplate HOT;Trough 25ml W3 second Trough;;

998;SD;;Extd Liquid 1 and 4;;Extd Liquid 3 and 6;W3second;;

998;0;

998;0;

998;0;

998;0;

998;0;

998;0;

998;0;

998;0;

998;0;

998;0;

998;0;

998;0;

998;0;

998;0;

998;0;

998;0;

998;0;

998;0;

998;0;

998;0;

998;0;

998;0;

998;0;

998;0;

998;0;

998;0;

998;0;

998;0;

998;0;

998;0;

998;0;

998;0;

998;0;

998;0;

998;0;

998;0;

998;0;

998;0;

998;0;

998;0;

998;0;

998;0;

998;0;

998;0;

998;0;

998;0;

998;0;

998;0;

998;0;

998;0;

998;0;

998;0;

998;0;

998;0;

998;0;

998;0;

998;0;

998;0;

998;0;

998;0;

998;0;

998;0;

998;0;

998;2;

998;86;41;

998;241;10;

998;5;

998;4;0;System;

998;0;3;Magellan;

998;0;0;Thermal Cycler;

998;0;1;Hotel 4Pos DeepWell;

998;0;2;Hotel 2Pos DitiRack 1;

998;3;

998;236;96 Well PCR Plate;

998;86;96 Well PCR Plate;

998;241;DiTi 200 ul;

998;1;

998;1;

998;45;

998;41;

998;10;

996;0;0;

--{ RPG }--

Notification(1,"EVO Team","Error: EVO 1st Information","Error",1);

Comment("Mixing step of Enzyme was changed.Oct 19th Tera");

Comment("Wash Buffer 1,3 was removed Oct19 Tera");

Comment("50ul Diti has been applied, Oct 7th 2010 Tera");

Comment("***Start Position : Cooling Rack***");

Comment("Change the name of loop 18th Feb Kojima");

Variable(TEST_MODE,"1",0,"TEST=0, REAL SUMPLE=1",0,0.000000,1.000000,0,2,0,0);

Variable(NC,"12",1,"How many lane ?",1,1.000000,12.000000,0,2,1,0);

Set_DITI_Counter2("DiTi 200 ul","8","1","1",0);

Set_DITI_Counter2("DiTi 50ul 2","8","3","1",0);

Command("C5SSP800,90,0",1,1,,,2,2,0);

Vector("Roma FAR RIGHT","35","5",0,1,2,2,1,0);

Group("(4) RNase One");

Comment("Modified 10.19.2010");

BeginLoop("NC","Add RNase One mixture to sample ");

Comment("Mixing step of Enzyme was changed.Oct 19th Tera");

Wash(255,1,1,1,0,"5",500,"1",500,10,70,30,1,0,1000,0);

GetDITI2(255,"DiTi 50ul 2",1,0,10,70);

Aspirate(255,"Clearing BEAD contents in TIP","5","5","5","5","5","5","5","5",0,0,0,0,32,2,1,"0108ｯ1",0,0);

Mix(255,"Enzyme Liquid Level","7","7","7","7","7","7","7","7",0,0,0,0,32,2,1,"0108ｯ1",2,0,0);

Aspirate(255,"ROM 5ul wet contact dispense2","5","5","5","5","5","5","5","5",0,0,0,0,32,2,1,"0108ｯ1",0,0);

Dispense(255,"ROM 5ul wet contact dispense2","5","5","5","5","5","5","5","5",0,0,0,0,8,3,1,"0C08ｯ1000000000000",1,"Add RNase One mixture to sample ",0,1,0);

Aspirate(255,"Aspiration Small Vol","3","3","3","3","3","3","3","3",0,0,0,0,8,3,1,"0C08ｯ1000000000000",1,"Add RNase One mixture to sample ",0,1,0);

BeginLoop("5","Sample mixing");

Aspirate(255,"Mixing RNaseONE inside Plate Slower","32","32","32","32","32","32","32","32",0,0,0,0,8,3,1,"0C08ｯ1000000000000",1,"Add RNase One mixture to sample ",0,1,0);

Dispense(255,"Mixing RNaseONE inside Plate Slower","32","32","32","32","32","32","32","32",0,0,0,0,8,3,1,"0C08ｯ1000000000000",1,"Add RNase One mixture to sample ",0,1,0);

EndLoop();

Aspirate(255,"Mixing RNaseONE inside Plate Slower","32","32","32","32","32","32","32","32",0,0,0,0,8,3,1,"0C08ｯ1000000000000",1,"Add RNase One mixture to sample ",0,1,0);

Dispense(255,"Mixing RNaseONE inside Plate Slower2","32","32","32","32","32","32","32","32",0,0,0,0,8,3,1,"0C08ｯ1000000000000",1,"Add RNase One mixture to sample ",0,1,0);

Dispense(255,"Aspiration Small Vol","3","3","3","3","3","3","3","3",0,0,0,0,8,3,1,"0C08ｯ1000000000000",1,"Add RNase One mixture to sample ",0,1,0);

StartTimer("1");

WaitTimer("1","2");

Dispense(255,"Blow out slow","5","5","5","5","5","5","5","5",0,0,0,0,8,3,1,"0C08ｯ1000000000000",1,"Add RNase One mixture to sample ",0,1,0);

Aspirate(255,"Clearing BEAD contents in TIP","25","25","25","25","25","25","25","25",0,0,0,0,8,3,1,"0C08ｯ1000000000000",1,"Add RNase One mixture to sample ",0,1,0);

Dispense(255,"Clearing BEAD contents in TIP","25","25","25","25","25","25","25","25",0,0,0,0,8,3,1,"0C08ｯ1000000000000",1,"Add RNase One mixture to sample ",0,1,0);

Detect_Liquid(255,"Detect liqid",8,3,1,"0C08ｯ1000000000000",1,"Add RNase One mixture to sample ",0,1,0);

DropDITI(255,2,2,10,70,0);

EndLoop();

Comment("End Modified 10.19.2010");

Comment("Thermal Cycler");

FACTS("ThermalCycler","ThermalCycler_OpenLid","1","0","");

Vector("FourDegreesPlate <-> SAFE","8","4",0,1,0,1,0,0);

Vector("SAFE <> Cycler","45","1",0,1,2,0,0,0);

Vector("SAFE <> Cycler","45","1",0,0,0,1,1,0);

Vector("SAFE <> Cycler","45","1",1,0,0,2,1,0);

FACTS("ThermalCycler","ThermalCycler_CloseLid","1,0","0","");

StartTimer("1");

FACTS("ThermalCycler","ThermalCycler_RunBlock","1,37-30MIN,BLOCK,ON,1800","0","");

WaitTimer("1","1810");

FACTS("ThermalCycler","ThermalCycler_OpenLid","1","0","");

Vector("SAFE <> Cycler","45","1",0,1,0,1,0,0);

Vector("FourDegreesPlate <-> SAFE","8","4",0,1,2,0,0,0);

Vector("FourDegreesPlate <->PUSHdown","8","4",0,1,0,0,1,0);

ROMA(2,80,75,0,0,0,150,1,0);

Comment("Thermal Cycler End");

GroupEnd();

Notification(0,"EVO Team","Assay finish","Assay finish",0);

**---END---**

**4.2. RNaseONE_CAPTRAPPING_REREASE**

**---START---**

9DC78045

20110627_162602 Admin

Administrator

--{ RES }--

V;200

--{ CFG }--

999;219;32;

14;-1;30;8;-1;-1;-1;-1;-1;234;-1;-1;-1;-1;-1;-1;96;-1;-1;-1;-1;-1;34;-1;-1;-1;-1;-1;246;244;245;243;242;238;-1;-1;239;-1;-1;-1;-1;-1;-1;-1;-1;-1;-1;-1;-1;-1;-1;-1;-1;-1;-1;-1;-1;-1;-1;-1;-1;-1;-1;-1;-1;-1;-1;-1;-1;-1;-1;-1;-1;-1;-1;-1;-1;-1;-1;-1;-1;-1;-1;-1;-1;-1;-1;-1;-1;-1;-1;-1;-1;-1;-1;-1;-1;-1;-1;-1;

998;0;

998;3;Wash Station Cleaner shallow;Wash Station Waste;Wash Station Cleaner deep;

998;;;;

998;3;;DiTi 200 ul EtOH USE;DiTi Waste;

998;;Diti 200- EtOH USE;Waste;

998;0;

998;0;

998;0;

998;0;

998;0;

998;4;DiTi 200 ul;DiTi 200 ul;DiTi 50ul 2;96 Well PCR Plate;

998;Diti 200 -A;Diti 200 -B;DiTi 50ul;Cold PCR plate;

998;0;

998;0;

998;0;

998;0;

998;0;

998;0;

998;3;DiTi 200 ul MIX1;DiTi 200 ul MIX2;DiTi 200 ul MIX3;

998;Diti AMP and CAPT 2;Diti AMP and CAPT 3;Diti AMP and CAPT 4;

998;0;

998;0;

998;0;

998;0;

998;0;

998;3;96 Well RT PCR;96 Well MAGNET PCR;;

998;RT PCR plate;MAGNET PCR;;

998;0;

998;0;

998;0;

998;0;

998;0;

998;3;;;;

998;;;;

998;3;;Trough 100ml W2;Trough 100ml ET 100percent;

998;;W2;BeadsWaste;

998;3;Trough 25ml ROB;;Trough 25ml;

998;Rob;;W4;

998;3;;;Tube 8pos MPG;

998;;;Mpg;

998;3;;;Trough 100ml ET 2;

998;;;E100;

998;6;;;0.5 ml Eppendorf Tube ROM;;;;

998;;;Rom;;;;

998;0;

998;0;

998;7;Trough 25ml SD;;96 Well Microplate HOT;;96 Well Microplate HOT;Trough 25ml W3 second Trough;;

998;SD;;Extd Liquid 1 and 4;;Extd Liquid 3 and 6;W3second;;

998;0;

998;0;

998;0;

998;0;

998;0;

998;0;

998;0;

998;0;

998;0;

998;0;

998;0;

998;0;

998;0;

998;0;

998;0;

998;0;

998;0;

998;0;

998;0;

998;0;

998;0;

998;0;

998;0;

998;0;

998;0;

998;0;

998;0;

998;0;

998;0;

998;0;

998;0;

998;0;

998;0;

998;0;

998;0;

998;0;

998;0;

998;0;

998;0;

998;0;

998;0;

998;0;

998;0;

998;0;

998;0;

998;0;

998;0;

998;0;

998;0;

998;0;

998;0;

998;0;

998;0;

998;0;

998;0;

998;0;

998;0;

998;0;

998;0;

998;0;

998;0;

998;0;

998;0;

998;2;

998;86;41;

998;241;10;

998;5;

998;4;0;System;

998;0;3;Magellan;

998;0;0;Thermal Cycler;

998;0;1;Hotel 4Pos DeepWell;

998;0;2;Hotel 2Pos DitiRack 1;

998;3;

998;236;96 Well PCR Plate;

998;86;96 Well PCR Plate;

998;241;DiTi 200 ul;

998;1;

998;1;

998;45;

998;41;

998;10;

996;0;0;

--{ RPG }--

Notification(1,"EVO Team","Error: EVO 1st Information","Error",1);

Comment("Mixing step of Enzyme was changed.Oct 19th Tera");

Comment("Wash Buffer 1,3 was removed Oct19 Tera");

Comment("50ul Diti has been applied, Oct 7th 2010 Tera");

Comment("***Start Position : Cooling Rack***");

Comment("change name of loop 22th Feb Kojima");

Comment("change script 22th Feb Kojima");

Comment("change name of loop 2nd Mar Kojima");

Comment("change Release buffer volume 20th Apr. Kojima");

Variable(TEST_MODE,"1",1,"TEST=0, REAL SUMPLE=1",1,0.000000,1.000000,0,2,1,0);

Variable(NC,"12",1,"How many lane？",1,1.000000,12.000000,0,2,1,0);

Set_DITI_Counter2("DiTi 200 ul","8","1","1",0);

Vector("Roma FAR RIGHT","35","5",0,1,2,2,1,0);

Group("(5) Cap-trapping");

Comment("(9) Cap-trapping");

Comment("MPG beads 105uL");

Group("Mixing the MPG beads in Ependorf Tube");

Wash(255,1,1,1,0,"2",500,"1",500,10,70,30,1,0,1000,0);

PickUp_DITIs2(255,2,1,"0C0800000000ｯ10000",0,"DiTi 200 ul EtOH USE",0);

Aspirate(255,"Aspirate Low Volume Liquid Level","5","5","5","5","5","5","5","5",0,0,0,0,30,2,1,"0108ｯ1",0,0);

Mix(255,"Mixing for MPG beads in trough close to Z-max","170","170","170","170","170","170","170","170",0,0,0,0,30,2,1,"0108ｯ1",10,0,0);

BeginLoop("5","Mix AMPure beads");

Aspirate(255,"Mixing for MPG beads in trough close to Z-max","180","180","180","180","180","180","180","180",0,0,0,0,30,2,1,"0108ｯ1",0,0);

Dispense(255,"Mixing MPG dropplets high position","180","180","180","180","180","180","180","180",0,0,0,0,30,2,1,"0108ｯ1",0,0);

EndLoop();

Dispense(255,"Low Volume wet contact dispense with tracking HIGHER Liq Positn","5","5","5","5","5","5","5","5",0,0,0,0,30,2,1,"0108ｯ1",0,0);

Detect_Liquid(255,"Contact dispense with tracking HIGH Liq Positn",30,2,1,"0108ｯ1",0,0);

DropDITI(255,2,2,10,70,0);

GroupEnd();

BeginLoop("NC","Add MPG Beads to sample ");

Wash(255,1,1,1,0,"2",500,"1",500,10,70,30,1,0,1000,0);

Subroutine("C:¥Program Files¥TECAN¥EVOware¥database¥Scripts¥Diluter_Aspirate_Air.esc",0);

PickUp_DITIs2(255,15,0,"0C08ｯ1000000000000",1,"Add MPG Beads to sample ",0,1,"DiTi 200 ul MIX1",0);

MoveLiha(255,15,0,1,"0C08ｯ1000000000000",4,3,25,400,1,"Add MPG Beads to sample ",0,1,0);

Subroutine("C:¥Program Files¥TECAN¥EVOware¥database¥Scripts¥Diluter_Dispense_Air.esc",0);

Mix(255,"Mixing for MPG beads in trough middle position","170","170","170","170","170","170","170","170",0,0,0,0,30,2,1,"0108ｯ1",5,0,0);

BeginLoop("3","Mix AMPure beads");

Aspirate(255,"Mixing for MPG beads in trough close to Z-max","180","180","180","180","180","180","180","180",0,0,0,0,30,2,1,"0108ｯ1",0,0);

Dispense(255,"Mixing MPG dropplets high position","180","180","180","180","180","180","180","180",0,0,0,0,30,2,1,"0108ｯ1",0,0);

EndLoop();

Aspirate(255,"MPG Beads","105","105","105","105","105","105","105","105",0,0,0,0,30,2,1,"0108ｯ1",0,0);

Detect_Liquid(255,"AMPure and Beads",30,2,1,"0108ｯ1",0,0);

Dispense(255,"MPG Beads","105","105","105","105","105","105","105","105",0,0,0,0,8,3,1,"0C08ｯ1000000000000",1,"Add MPG Beads to sample ",0,1,0);

Aspirate(255,"Aspirate Low Volume Liquid Level","10","10","10","10","10","10","10","10",0,0,0,0,8,3,1,"0C08ｯ1000000000000",1,"Add MPG Beads to sample ",0,1,0);

IfThen("TEST_MODE",0,"0");

Mix(255,"Mixing Ampure inside Plate","100","100","100","100","100","100","100","100",0,0,0,0,8,3,1,"0C08ｯ1000000000000",5,1,"Add MPG Beads to sample ",0,1,0);

Else();

Mix(255,"Mixing Ampure inside Plate","100","100","100","100","100","100","100","100",0,0,0,0,8,3,1,"0C08ｯ1000000000000",10,1,"Add MPG Beads to sample ",0,1,0);

EndIf();

Dispense(255,"Low Volume wet contact dispense with tracking HIGHER Liq Positn","10","10","10","10","10","10","10","10",0,0,0,0,8,3,1,"0C08ｯ1000000000000",1,"Add MPG Beads to sample ",0,1,0);

Detect_Liquid(255,"Low Volume wet contact dispense with tracking HIGHER Liq Positn",8,3,1,"0C08ｯ1000000000000",1,"Add MPG Beads to sample ",0,1,0);

Aspirate(255,"Clearing BEAD contents in TIP","20","20","20","20","20","20","20","20",0,0,0,0,8,3,1,"0C08ｯ1000000000000",1,"Add MPG Beads to sample ",0,1,0);

Dispense(255,"Clearing BEAD contents in TIP Zmax -17","20","20","20","20","20","20","20","20",0,0,0,0,8,3,1,"0C08ｯ1000000000000",1,"Add MPG Beads to sample ",0,1,0);

Detect_Liquid(255,"Low Volume wet contact dispense with tracking HIGHER Liq Positn",8,3,1,"0C08ｯ1000000000000",1,"Add MPG Beads to sample ",0,1,0);

Set_DITIs_Back(255,15,0,"0C08ｯ1000000000000",1,"Add MPG Beads to sample ",0,1,0);

EndLoop();

Group("TIMER ( 30 mins mix every 3 mins )");

Comment("1 min 12s per colunm x 12 equals 14 mins 24 s (2 columns + 3mins wait cycle = 32 mins total)");

Vector("FourDegreesPlate <-> SAFE","8","4",0,1,0,1,0,0);

Vector("ExtractedLiquid <> SAFE","35","5",0,1,2,0,0,0);

Vector("ExtractedLiquid <> PUSHdown","35","5",0,1,0,0,0,0);

Vector("ExtractedLiquid <> SAFE","35","5",0,0,0,1,0,0);

Vector("ExtractedLiquid <> SAFE","35","5",1,0,0,2,0,0);

ROMA(2,80,75,0,0,0,150,1,0);

BeginLoop("3","Mix the sample and MPG beads for Capture");

BeginLoop("NC","Mix the sample and MPG beads");

Wash(255,1,1,1,0,"2",500,"1",500,10,70,30,1,0,1000,0);

Subroutine("C:¥Program Files¥TECAN¥EVOware¥database¥Scripts¥Diluter_Aspirate_Air.esc",0);

PickUp_DITIs2(255,15,0,"0C08ｯ1000000000000",1,"Mix the sample and MPG beads",0,1,"DiTi 200 ul MIX1",0);

MoveLiha(255,15,0,1,"0C08ｯ1000000000000",4,3,25,400,1,"Mix the sample and MPG beads",0,1,0);

Subroutine("C:¥Program Files¥TECAN¥EVOware¥database¥Scripts¥Diluter_Dispense_Air.esc",0);

Aspirate(255,"Low Volume 5ul aspiration and dispense","10","10","10","10","10","10","10","10",0,0,0,0,35,4,1,"0C08ｯ1000000000000",1,"Mix the sample and MPG beads",0,1,0);

Mix(255,"Mixing MPG inside Plate Large Volume MIX","100","100","100","100","100","100","100","100",0,0,0,0,35,4,1,"0C08ｯ1000000000000",5,1,"Mix the sample and MPG beads",0,1,0);

BeginLoop("5","Sample and MPG beads mixing");

Aspirate(255,"Mixing MPG inside Plate Large Volume MIX","100","100","100","100","100","100","100","100",0,0,0,0,35,4,1,"0C08ｯ1000000000000",1,"Mix the sample and MPG beads",0,1,0);

Dispense(255,"Mixing MPG inside Plate Large Volume MIX","100","100","100","100","100","100","100","100",0,0,0,0,35,4,1,"0C08ｯ1000000000000",1,"Mix the sample and MPG beads",0,1,0);

EndLoop();

Dispense(255,"Low Volume wet contact dispense with tracking HIGHER Liq Positn","10","10","10","10","10","10","10","10",0,0,0,0,35,4,1,"0C08ｯ1000000000000",1,"Mix the sample and MPG beads",0,1,0);

Detect_Liquid(255,"Mixing Ampure inside Plate Large Volume MIX",35,4,1,"0C08ｯ1000000000000",1,"Mix the sample and MPG beads",0,1,0);

Aspirate(255,"Clearing BEAD contents in TIP","100","100","100","100","100","100","100","100",0,0,0,0,35,4,1,"0C08ｯ1000000000000",1,"Mix the sample and MPG beads",0,1,0);

Dispense(255,"Clearing BEAD contents in TIP Zmax -17","100","100","100","100","100","100","100","100",0,0,0,0,35,4,1,"0C08ｯ1000000000000",1,"Mix the sample and MPG beads",0,1,0);

Detect_Liquid(255,"Mixing Ampure inside Plate Large Volume MIX",35,4,1,"0C08ｯ1000000000000",1,"Mix the sample and MPG beads",0,1,0);

Set_DITIs_Back(255,15,0,"0C08ｯ1000000000000",1,"Mix the sample and MPG beads",0,1,0);

EndLoop();

Comment("Remove the Timer (wait for 1min) 22th Feb Kojima ");

EndLoop();

GroupEnd();

Comment("Move Extracted Liquid3 --> Magnet");

Vector("ExtractedLiquid <> SAFE","35","5",0,1,0,1,1,0);

Vector("SAFE <> Magnet","21","2",0,1,2,0,1,0);

Vector("SAFE <> PUSH down Magnet","21","2",0,1,0,2,0,0);

Vector("Roma FAR RIGHT","35","5",0,1,2,0,1,0);

StartTimer("1");

WaitTimer("1","5 * 60");

Comment("Cut Out 10.19.2010");

Comment("Wash Buffer 2 150uL");

Comment("Change Labware of beads waste (32-4) -> (28-1) 22th Mar Kojima");

Group("Wash Buffer 2");

BeginLoop("NC","Remove supernatant and Add wash Buffer 2 to sample ");

Wash(255,1,1,1,0,"2",500,"1",500,10,70,30,1,0,1000,0);

GetDITI2(255,"DiTi 200 ul",0,0,10,70);

Aspirate(255,"Remove Excess liquid from Magnet Plate for SDS Wash Buffer","110","110","110","110","110","110","110","110",0,0,0,0,21,1,1,"0C08ｯ1000000000000",1,"Remove supernatant and Add wash Buffer 2 to sample ",0,1,0);

Dispense(255,"Clearing contents of TIP","110","110","110","110","110","110","110","110",0,0,0,0,28,2,1,"0108ｯ1",0,0);

DropDITI(255,2,2,10,70,0);

Comment("Wash Buffer 2 150ul Beads retained submerged in Liquid");

Comment("Chnage buffer dispense position (3 --> -1mm) 6th Apr Kojima");

Wash(255,1,1,1,0,"2",500,"1",500,10,70,30,1,0,1000,0);

PickUp_DITIs2(255,2,1,"0C0800ｬ70000000000",0,"DiTi 200 ul EtOH USE",0);

Aspirate(255,"Aspirate WASH Volume Liquid Level","150","150","150","150","150","150","150","150",0,0,0,0,28,1,1,"0108ｯ1",0,0);

Dispense(255,"Water free dispense blow out","150","150","150","150","150","150","150","150",0,0,0,0,21,1,1,"0C08ｯ1000000000000",1,"Remove supernatant and Add wash Buffer 2 to sample ",0,1,0);

Set_DITIs_Back(255,2,1,"0C0800ｬ70000000000",0,0);

EndLoop();

GroupEnd();

Comment("Wash Buffer 2 150ul");

Comment("Move from Magnet --> to SHIFT position");

Vector("SAFE <> Magnet","21","2",0,1,0,1,1,0);

Vector("MAGNET SHIFT UP Pos1","21","2",0,0,2,2,1,0);

StartTimer("1");

WaitTimer("1","30");

Vector("MAGNET SHIFT UP Pos2","21","2",0,0,2,2,1,0);

StartTimer("1");

WaitTimer("1","30");

Vector("MAGNET SHIFT UP Pos3","21","2",0,0,2,2,1,0);

StartTimer("1");

WaitTimer("1","30");

Vector("MAGNET SHIFT UP Pos4","21","2",0,0,2,2,1,0);

StartTimer("1");

WaitTimer("1","30");

Vector("MAGNET SHIFT UP Pos5","21","2",1,0,2,2,1,0);

Vector("RT Assay 1 <> SAFE","21","1",0,1,2,0,1,0);

Vector("RT Assay 1 <> PUSHdown","21","1",0,1,0,0,0,0);

Vector("Roma FAR RIGHT","35","5",0,1,0,0,1,0);

Comment("Mixing Wash Buffer 2");

Comment("Correct the labware Aspirate/Dispense 5uL (35-1) -> (21-1) 22th Mar Kojima");

Group("Mixing wash Buffer 2");

BeginLoop("NC","Mix the sample and wash Buffer 2");

Wash(255,1,1,1,0,"2",500,"1",500,10,70,30,1,0,1000,0);

Subroutine("C:¥Program Files¥TECAN¥EVOware¥database¥Scripts¥Diluter_Aspirate_Air.esc",0);

PickUp_DITIs2(255,15,0,"0C08ｯ1000000000000",1,"Mix the sample and wash Buffer 2",0,1,"DiTi 200 ul MIX1",0);

MoveLiha(255,15,0,1,"0C08ｯ1000000000000",4,3,25,400,1,"Mix the sample and wash Buffer 2",0,1,0);

Subroutine("C:¥Program Files¥TECAN¥EVOware¥database¥Scripts¥Diluter_Dispense_Air.esc",0);

Aspirate(255,"WaterKind W3","5","5","5","5","5","5","5","5",0,0,0,0,21,0,1,"0C08ｯ1000000000000",1,"Mix the sample and wash Buffer 2",0,1,0);

BeginLoop("5","Sample and wash buffer 2 mixing ");

Aspirate(255,"MPG beads LEFT side mix","100","100","100","100","100","100","100","100",0,0,0,0,21,0,1,"0C08ｯ1000000000000",1,"Mix the sample and wash Buffer 2",0,1,0);

Dispense(255,"MPG beads LEFT side mix","100","100","100","100","100","100","100","100",0,0,0,0,21,0,1,"0C08ｯ1000000000000",1,"Mix the sample and wash Buffer 2",0,1,0);

Aspirate(255,"MPG beads RIGHT side mix","100","100","100","100","100","100","100","100",0,0,0,0,21,0,1,"0C08ｯ1000000000000",1,"Mix the sample and wash Buffer 2",0,1,0);

Dispense(255,"MPG beads RIGHT side mix","100","100","100","100","100","100","100","100",0,0,0,0,21,0,1,"0C08ｯ1000000000000",1,"Mix the sample and wash Buffer 2",0,1,0);

EndLoop();

Dispense(255,"Low Volume wet contact dispense with tracking HIGHER Liq Positn","5","5","5","5","5","5","5","5",0,0,0,0,21,0,1,"0C08ｯ1000000000000",0,0);

Detect_Liquid(255,"Low Volume wet contact dispense with tracking HIGHER Liq Positn",21,0,1,"0C08ｯ1000000000000",1,"Mix the sample and wash Buffer 2",0,1,0);

Aspirate(255,"Clearing BEAD contents in TIP","100","100","100","100","100","100","100","100",0,0,0,0,21,0,1,"0C08ｯ1000000000000",1,"Mix the sample and wash Buffer 2",0,1,0);

Dispense(255,"Clearing BEAD contents in TIP Zmax -17","100","100","100","100","100","100","100","100",0,0,0,0,21,0,1,"0C08ｯ1000000000000",1,"Mix the sample and wash Buffer 2",0,1,0);

Detect_Liquid(255,"Low Volume wet contact dispense with tracking HIGHER Liq Positn",21,0,1,"0C08ｯ1000000000000",1,"Mix the sample and wash Buffer 2",0,1,0);

Set_DITIs_Back(255,15,0,"0C08ｯ1000000000000",1,"Mix the sample and wash Buffer 2",0,1,0);

EndLoop();

MoveLiha(255,1,1,1,"0108ｯ1",0,0,0,10,0,0);

Vector("RT Assay 1 <> SAFE","21","1",0,1,0,1,1,0);

Vector("SAFE <> Magnet","21","2",0,1,2,0,1,0);

Vector("SAFE <> PUSH down Magnet","21","2",0,1,0,2,1,0);

Vector("Roma FAR RIGHT","35","5",0,1,0,0,1,0);

StartTimer("1");

WaitTimer("1","5 * 60");

Comment("Remove ");

Comment("Cut out 10.19.2010 ");

GroupEnd();

Comment("Cut out 10.19.2010 ");

Comment("Add wash Buffer 3 ");

BeginLoop("NC","Remove supernatant (WB 2) and Add wash buffer 3");

Wash(255,1,1,1,0,"2",500,"1",500,10,70,30,1,0,1000,0);

Subroutine("C:¥Program Files¥TECAN¥EVOware¥database¥Scripts¥Diluter_Aspirate_Air.esc",0);

PickUp_DITIs2(255,15,1,"0C08ｯ1000000000000",1,"Remove supernatant (WB 2) and Add wash buffer 3",0,1,"DiTi 200 ul MIX2",0);

MoveLiha(255,15,1,1,"0C08ｯ1000000000000",4,3,25,400,1,"Remove supernatant (WB 2) and Add wash buffer 3",0,1,0);

Subroutine("C:¥Program Files¥TECAN¥EVOware¥database¥Scripts¥Diluter_Dispense_Air.esc",0);

Aspirate(255,"Remove Excess liquid from Magnet Plate for SDS Wash Buffer","90","90","90","90","90","90","90","90",0,0,0,0,21,1,1,"0C08ｯ1000000000000",1,"Remove supernatant (WB 2) and Add wash buffer 3",0,1,0);

Dispense(255,"Clearing contents of TIP","90","90","90","90","90","90","90","90",0,0,0,0,28,2,1,"0108ｯ1",0,0);

Aspirate(255,"Remove Excess liquid from Magnet Plate for SDS Wash Buffer","90","90","90","90","90","90","90","90",0,0,0,0,21,1,1,"0C08ｯ1000000000000",1,"Remove supernatant (WB 2) and Add wash buffer 3",0,1,0);

Dispense(255,"Clearing contents of TIP","90","90","90","90","90","90","90","90",0,0,0,0,28,2,1,"0108ｯ1",0,0);

Set_DITIs_Back(255,15,1,"0C08ｯ1000000000000",1,"Remove supernatant (WB 2) and Add wash buffer 3",0,1,0);

Wash(255,1,1,1,0,"2",500,"1",500,10,70,30,1,0,1000,0);

Comment("Wash 3 Second run through");

Comment("Chnage buffer dispense position (3 --> -1mm) 6th Apr Kojima");

PickUp_DITIs2(255,2,1,"0C080000 O00000000",0,"DiTi 200 ul EtOH USE",0);

Aspirate(255,"Aspirate WASH Volume Liquid Level","150","150","150","150","150","150","150","150",0,0,0,0,35,5,1,"0108ｯ1",0,0);

Dispense(255,"Water free dispense blow out","150","150","150","150","150","150","150","150",0,0,0,0,21,1,1,"0C08ｯ1000000000000",1,"Remove supernatant (WB 2) and Add wash buffer 3",0,1,0);

Set_DITIs_Back(255,2,1,"0C080000 O00000000",0,0);

EndLoop();

Vector("SAFE <> Magnet","21","2",0,1,0,1,0,0);

Vector("ExtractedLiquid <> SAFE","35","5",0,1,2,0,0,0);

Vector("ExtractedLiquid <> PUSHdown","35","5",0,1,0,0,0,0);

StartTimer("1");

WaitTimer("1","3 * 60");

Vector("ExtractedLiquid <> SAFE","35","5",0,1,0,1,0,0);

Vector("MAGNET SHIFT UP Pos5","21","2",0,0,2,2,1,0);

Vector("MAGNET SHIFT UP Pos4","21","2",0,1,2,2,0,0);

StartTimer("1");

WaitTimer("1","1 * 60");

Vector("MAGNET SHIFT UP Pos5","21","2",1,0,2,2,1,0);

Vector("ExtractedLiquid <> SAFE","35","5",0,1,2,0,0,0);

Vector("ExtractedLiquid <> PUSHdown","35","5",0,1,0,0,0,0);

Vector("Roma FAR RIGHT","35","5",0,1,0,0,0,0);

Comment("Delete Wash tip 28th Mar Kojima");

Comment("Mixing Wash Buffer 3");

Comment("Change mix volume (30uL --> 50uL) 24th Mar Kojima ");

Comment("Change mix position (wb3; -9mm --> -6mm) 24th Mar Kojima ");

Comment("Change mix time (3 mix --> 4 mix) 24th Mar Kojima ");

BeginLoop("NC","Mixing the sample and wash buffer 3");

Wash(255,1,1,1,0,"2",500,"1",500,10,70,30,1,0,1000,0);

Subroutine("C:¥Program Files¥TECAN¥EVOware¥database¥Scripts¥Diluter_Aspirate_Air.esc",0);

PickUp_DITIs2(255,15,1,"0C08ｯ1000000000000",1,"Mixing the sample and wash buffer 3",0,1,"DiTi 200 ul MIX2",0);

MoveLiha(255,15,1,1,"0C08ｯ1000000000000",4,3,25,400,1,"Mixing the sample and wash buffer 3",0,1,0);

Subroutine("C:¥Program Files¥TECAN¥EVOware¥database¥Scripts¥Diluter_Dispense_Air.esc",0);

Aspirate(255,"WaterKind W3","5","5","5","5","5","5","5","5",0,0,0,0,35,4,1,"0C08ｯ1000000000000",1,"Mixing the sample and wash buffer 3",0,1,0);

IfThen("TEST_MODE",0,"0");

Mix(255,"Mixing Shift Left WashBuffer3 inside plate","30","30","30","30","30","30","30","30",0,0,0,0,35,4,1,"0C08ｯ1000000000000",5,1,"Mixing the sample and wash buffer 3",0,1,0);

Else();

Mix(255,"Mixing Shift Left WashBuffer3 inside plate","50","50","50","50","50","50","50","50",0,0,0,0,35,4,1,"0C08ｯ1000000000000",4,1,"Mixing the sample and wash buffer 3",0,1,0);

EndIf();

MoveLiha(255,35,4,1,"0C08ｯ1000000000000",0,4,0,10,1,"Mixing the sample and wash buffer 3",0,1,0);

IfThen("TEST_MODE",0,"0");

Mix(255,"Mixing Shift Right WashBuffer3 inside plate","30","30","30","30","30","30","30","30",0,0,0,0,35,4,1,"0C08ｯ1000000000000",5,1,"Mixing the sample and wash buffer 3",0,1,0);

Else();

Mix(255,"Mixing Shift Right WashBuffer3 inside plate","50","50","50","50","50","50","50","50",0,0,0,0,35,4,1,"0C08ｯ1000000000000",4,1,"Mixing the sample and wash buffer 3",0,1,0);

EndIf();

MoveLiha(255,35,4,1,"0C08ｯ1000000000000",0,4,0,10,1,"Mixing the sample and wash buffer 3",0,1,0);

IfThen("TEST_MODE",0,"0");

Mix(255,"Mixing Shift Rear WashBuffer3 inside plate","30","30","30","30","30","30","30","30",0,0,0,0,35,4,1,"0C08ｯ1000000000000",5,1,"Mixing the sample and wash buffer 3",0,1,0);

Else();

Mix(255,"Mixing Shift Rear WashBuffer3 inside plate","50","50","50","50","50","50","50","50",0,0,0,0,35,4,1,"0C08ｯ1000000000000",4,1,"Mixing the sample and wash buffer 3",0,1,0);

EndIf();

MoveLiha(255,35,4,1,"0C08ｯ1000000000000",0,4,0,10,1,"Mixing the sample and wash buffer 3",0,1,0);

IfThen("TEST_MODE",0,"0");

Mix(255,"Mixing Shift Front WashBuffer3 inside plate","30","30","30","30","30","30","30","30",0,0,0,0,35,4,1,"0C08ｯ1000000000000",5,1,"Mixing the sample and wash buffer 3",0,1,0);

Else();

Mix(255,"Mixing Shift Front WashBuffer3 inside plate","50","50","50","50","50","50","50","50",0,0,0,0,35,4,1,"0C08ｯ1000000000000",4,1,"Mixing the sample and wash buffer 3",0,1,0);

EndIf();

MoveLiha(255,35,4,1,"0C08ｯ1000000000000",0,4,0,10,1,"Mixing the sample and wash buffer 3",0,1,0);

Dispense(255,"Low Volume wet contact dispense with tracking HIGHER Liq Positn","5","5","5","5","5","5","5","5",0,0,0,0,35,4,1,"0C08ｯ1000000000000",1,"Mixing the sample and wash buffer 3",0,1,0);

Detect_Liquid(255,"Mixing Beads with Sample small fast",35,4,1,"0C08ｯ1000000000000",1,"Mixing the sample and wash buffer 3",0,1,0);

Aspirate(255,"Clearing BEAD contents in TIP","100","100","100","100","100","100","100","100",0,0,0,0,35,4,1,"0C08ｯ1000000000000",1,"Mixing the sample and wash buffer 3",0,1,0);

Dispense(255,"Clearing BEAD contents in TIP Zmax -17","100","100","100","100","100","100","100","100",0,0,0,0,35,4,1,"0C08ｯ1000000000000",1,"Mixing the sample and wash buffer 3",0,1,0);

Set_DITIs_Back(255,15,1,"0C08ｯ1000000000000",1,"Mixing the sample and wash buffer 3",0,1,0);

EndLoop();

Wash(255,1,1,1,0,"2",500,"1",500,10,70,30,1,0,1000,0);

MoveLiha(255,1,1,1,"0108ｯ1",0,0,0,10,0,0);

Vector("ExtractedLiquid <> SAFE","35","5",0,1,0,1,0,0);

Vector("SAFE <> Magnet","21","2",0,1,2,0,1,0);

Vector("SAFE <> PUSH down Magnet","21","2",0,1,0,2,1,0);

Vector("Roma FAR RIGHT","35","5",0,1,0,0,0,0);

Comment("Change Timer NO. (5 --> 1) 28th Mar Kojima");

StartTimer("1");

WaitTimer("1","5 * 60");

Comment("Remove -11");

Comment("Add Wash Buffer 4 ");

BeginLoop("NC","Remove supernatant (WB3) and Add wash buffer 4_1");

Wash(255,1,1,1,0,"2",500,"1",500,10,70,30,1,0,1000,0);

Subroutine("C:¥Program Files¥TECAN¥EVOware¥database¥Scripts¥Diluter_Aspirate_Air.esc",0);

PickUp_DITIs2(255,15,1,"0C08ｯ1000000000000",1,"Remove supernatant (WB3) and Add wash buffer 4_1",0,1,"DiTi 200 ul MIX2",0);

MoveLiha(255,15,1,1,"0C08ｯ1000000000000",4,3,25,400,1,"Remove supernatant (WB3) and Add wash buffer 4_1",0,1,0);

Subroutine("C:¥Program Files¥TECAN¥EVOware¥database¥Scripts¥Diluter_Dispense_Air.esc",0);

Aspirate(255,"Remove Excess liquid from Magnet Plate Wash Buffer Asp SLOW","150","150","150","150","150","150","150","150",0,0,0,0,21,1,1,"0C08ｯ1000000000000",1,"Remove supernatant (WB3) and Add wash buffer 4_1",0,1,0);

Dispense(255,"Clearing contents of TIP","150","150","150","150","150","150","150","150",0,0,0,0,28,2,1,"0108ｯ1",0,0);

Set_DITIs_Back(255,15,1,"0C08ｯ1000000000000",1,"Remove supernatant (WB3) and Add wash buffer 4_1",0,1,0);

Comment("Wash Buffer4 150ul");

Comment("Chnage buffer dispense position (3 --> -1mm) 6th Apr Kojima");

Wash(255,1,1,1,0,"2",500,"1",500,10,70,30,1,0,1000,0);

PickUp_DITIs2(255,2,1,"0C080000000000ｬ700",0,"DiTi 200 ul EtOH USE",0);

Aspirate(255,"Aspirate WASH Volume Liquid Level","150","150","150","150","150","150","150","150",0,0,0,0,29,2,1,"0108ｯ1",0,0);

Dispense(255,"Water free dispense blow out","150","150","150","150","150","150","150","150",0,0,0,0,21,1,1,"0C08ｯ1000000000000",1,"Remove supernatant (WB3) and Add wash buffer 4_1",0,1,0);

Set_DITIs_Back(255,2,1,"0C080000000000ｬ700",0,0);

EndLoop();

Vector("SAFE <> Magnet","21","2",0,1,0,1,0,0);

Vector("MAGNET SHIFT UP Pos5","21","2",0,0,2,2,1,0);

Vector("MAGNET SHIFT UP Pos4","21","2",0,1,2,2,0,0);

StartTimer("1");

WaitTimer("1","30");

Vector("MAGNET SHIFT UP Pos5","21","2",1,0,2,2,1,0);

Vector("RT Assay 1 <> SAFE","21","1",0,1,2,0,0,0);

Vector("RT Assay 1 <> PUSHdown","21","1",0,1,0,0,0,0);

Vector("Roma FAR RIGHT","35","5",0,1,0,0,0,0);

Wash(255,1,1,1,0,"2",500,"1",500,10,70,30,1,0,1000,0);

Comment("Mix Wash Buffer 4_1 ");

BeginLoop("NC","Mix the sample and wash Buffer 4_1");

Subroutine("C:¥Program Files¥TECAN¥EVOware¥database¥Scripts¥Diluter_Aspirate_Air.esc",0);

PickUp_DITIs2(255,15,2,"0C08ｯ1000000000000",1,"Mix the sample and wash Buffer 4_1",0,1,"DiTi 200 ul MIX3",0);

MoveLiha(255,15,2,1,"0C08ｯ1000000000000",4,3,25,400,1,"Mix the sample and wash Buffer 4_1",0,1,0);

Subroutine("C:¥Program Files¥TECAN¥EVOware¥database¥Scripts¥Diluter_Dispense_Air.esc",0);

Aspirate(255,"WaterKind W3","5","5","5","5","5","5","5","5",0,0,0,0,21,0,1,"0C08ｯ1000000000000",1,"Mix the sample and wash Buffer 4_1",0,1,0);

IfThen("TEST_MODE",0,"0");

Mix(255,"Mixing WashBuffer4 inside plate","100","100","100","100","100","100","100","100",0,0,0,0,21,0,1,"0C08ｯ1000000000000",5,1,"Mix the sample and wash Buffer 4_1",0,1,0);

Else();

Mix(255,"Mixing WashBuffer4 inside plate","100","100","100","100","100","100","100","100",0,0,0,0,21,0,1,"0C08ｯ1000000000000",10,1,"Mix the sample and wash Buffer 4_1",0,1,0);

EndIf();

Dispense(255,"Low Volume wet contact dispense with tracking HIGHER Liq Positn","5","5","5","5","5","5","5","5",0,0,0,0,21,0,1,"0C08ｯ1000000000000",1,"Mix the sample and wash Buffer 4_1",0,1,0);

Detect_Liquid(255,"Mixing Beads with Sample small fast",21,0,1,"0C08ｯ1000000000000",1,"Mix the sample and wash Buffer 4_1",0,1,0);

Aspirate(255,"Clearing BEAD contents in TIP","100","100","100","100","100","100","100","100",0,0,0,0,21,0,1,"0C08ｯ1000000000000",1,"Mix the sample and wash Buffer 4_1",0,1,0);

Dispense(255,"Clearing BEAD contents in TIP Zmax -17","100","100","100","100","100","100","100","100",0,0,0,0,21,0,1,"0C08ｯ1000000000000",1,"Mix the sample and wash Buffer 4_1",0,1,0);

Set_DITIs_Back(255,15,2,"0C08ｯ1000000000000",1,"Mix the sample and wash Buffer 4_1",0,1,0);

EndLoop();

Vector("RT Assay 1 <> SAFE","21","1",0,1,0,1,0,0);

Vector("SAFE <> Magnet","21","2",0,1,2,0,1,0);

Vector("SAFE <> PUSH down Magnet","21","2",0,1,0,2,1,0);

Vector("Roma FAR RIGHT","35","5",0,1,0,0,0,0);

StartTimer("1");

WaitTimer("1","5 * 60");

Comment("Remove -11");

Comment("Add wash Buffer 4_2");

BeginLoop("NC","Remove supernatant (WB4_1) and Add wash buffer 4_2");

Wash(255,1,1,1,0,"2",500,"1",500,10,70,30,1,0,1000,0);

Subroutine("C:¥Program Files¥TECAN¥EVOware¥database¥Scripts¥Diluter_Aspirate_Air.esc",0);

PickUp_DITIs2(255,15,2,"0C08ｯ1000000000000",1,"Remove supernatant (WB4_1) and Add wash buffer 4_2",0,1,"DiTi 200 ul MIX3",0);

MoveLiha(255,15,2,1,"0C08ｯ1000000000000",4,3,25,400,1,"Remove supernatant (WB4_1) and Add wash buffer 4_2",0,1,0);

Subroutine("C:¥Program Files¥TECAN¥EVOware¥database¥Scripts¥Diluter_Dispense_Air.esc",0);

Aspirate(255,"Remove Excess liquid from Magnet Plate Wash Buffer Asp SLOW","160","160","160","160","160","160","160","160",0,0,0,0,21,1,1,"0C08ｯ1000000000000",1,"Remove supernatant (WB4_1) and Add wash buffer 4_2",0,1,0);

Dispense(255,"Clearing contents of TIP","160","160","160","160","160","160","160","160",0,0,0,0,28,2,1,"0108ｯ1",0,0);

Set_DITIs_Back(255,15,2,"0C08ｯ1000000000000",1,"Remove supernatant (WB4_1) and Add wash buffer 4_2",0,1,0);

Comment("Chnage buffer dispense position (3 --> -1mm) 6th Apr Kojima");

Wash(255,1,1,1,0,"2",500,"1",500,10,70,30,1,0,1000,0);

PickUp_DITIs2(255,2,1,"0C080000000000ｬ700",0,"DiTi 200 ul EtOH USE",0);

Aspirate(255,"Aspirate WASH Volume Liquid Level","150","150","150","150","150","150","150","150",0,0,0,0,29,2,1,"0108ｯ1",0,0);

Dispense(255,"Water free dispense blow out","150","150","150","150","150","150","150","150",0,0,0,0,21,1,1,"0C08ｯ1000000000000",1,"Remove supernatant (WB4_1) and Add wash buffer 4_2",0,1,0);

Set_DITIs_Back(255,2,1,"0C080000000000ｬ700",0,0);

EndLoop();

Vector("SAFE <> Magnet","21","2",0,1,0,1,0,0);

Vector("MAGNET SHIFT UP Pos5","21","2",0,0,2,2,1,0);

Vector("MAGNET SHIFT UP Pos4","21","2",0,1,2,2,0,0);

StartTimer("1");

WaitTimer("1","30");

Vector("MAGNET SHIFT UP Pos5","21","2",1,0,2,2,1,0);

Vector("RT Assay 1 <> SAFE","21","1",0,1,2,0,0,0);

Vector("RT Assay 1 <> PUSHdown","21","1",0,1,0,0,0,0);

Vector("Roma FAR RIGHT","35","5",0,1,0,0,0,0);

Comment("Mix wash Buffer 4_2");

BeginLoop("NC","Mix the sample and wash buffer 4_2");

Wash(255,1,1,1,0,"2",500,"1",500,10,70,30,1,0,1000,0);

Subroutine("C:¥Program Files¥TECAN¥EVOware¥database¥Scripts¥Diluter_Aspirate_Air.esc",0);

PickUp_DITIs2(255,15,2,"0C08ｯ1000000000000",1,"Mix the sample and wash buffer 4_2",0,1,"DiTi 200 ul MIX3",0);

MoveLiha(255,15,2,1,"0C08ｯ1000000000000",4,3,25,400,1,"Mix the sample and wash buffer 4_2",0,1,0);

Subroutine("C:¥Program Files¥TECAN¥EVOware¥database¥Scripts¥Diluter_Dispense_Air.esc",0);

Aspirate(255,"WaterKind W3","5","5","5","5","5","5","5","5",0,0,0,0,21,0,1,"0C08ｯ1000000000000",1,"Mix the sample and wash buffer 4_2",0,1,0);

IfThen("TEST_MODE",0,"0");

Mix(255,"Mixing WashBuffer4 inside plate","100","100","100","100","100","100","100","100",0,0,0,0,21,0,1,"0C08ｯ1000000000000",5,1,"Mix the sample and wash buffer 4_2",0,1,0);

Else();

Mix(255,"Mixing WashBuffer4 inside plate","100","100","100","100","100","100","100","100",0,0,0,0,21,0,1,"0C08ｯ1000000000000",10,1,"Mix the sample and wash buffer 4_2",0,1,0);

EndIf();

Dispense(255,"Low Volume wet contact dispense with tracking HIGHER Liq Positn","5","5","5","5","5","5","5","5",0,0,0,0,21,0,1,"0C08ｯ1000000000000",1,"Mix the sample and wash buffer 4_2",0,1,0);

Detect_Liquid(255,"Mixing Beads with Sample small fast",21,0,1,"0C08ｯ1000000000000",1,"Mix the sample and wash buffer 4_2",0,1,0);

Aspirate(255,"Clearing BEAD contents in TIP","100","100","100","100","100","100","100","100",0,0,0,0,21,0,1,"0C08ｯ1000000000000",1,"Mix the sample and wash buffer 4_2",0,1,0);

Dispense(255,"Clearing BEAD contents in TIP Zmax -17","100","100","100","100","100","100","100","100",0,0,0,0,21,0,1,"0C08ｯ1000000000000",1,"Mix the sample and wash buffer 4_2",0,1,0);

Set_DITIs_Back(255,15,2,"0C08ｯ1000000000000",1,"Mix the sample and wash buffer 4_2",0,1,0);

EndLoop();

Wash(255,1,1,1,0,"2",500,"1",500,10,70,30,1,0,1000,0);

MoveLiha(255,1,1,1,"0108ｯ1",0,0,0,10,0,0);

Vector("RT Assay 1 <> SAFE","21","1",0,1,0,1,0,0);

Vector("SAFE <> Magnet","21","2",0,1,2,0,1,0);

Vector("SAFE <> PUSH down Magnet","21","2",0,1,0,2,1,0);

Vector("Roma FAR RIGHT","35","5",0,1,0,0,0,0);

StartTimer("1");

WaitTimer("1","5 * 60");

Comment("Remove -11");

BeginLoop("NC","Remove supernatant (WB4_2) and Add release buffer ");

Wash(255,1,1,1,0,"2",500,"1",500,10,70,30,1,0,1000,0);

Subroutine("C:¥Program Files¥TECAN¥EVOware¥database¥Scripts¥Diluter_Aspirate_Air.esc",0);

PickUp_DITIs2(255,15,2,"0C08ｯ1000000000000",1,"Remove supernatant (WB4_2) and Add release buffer ",0,1,"DiTi 200 ul MIX3",0);

MoveLiha(255,15,2,1,"0C08ｯ1000000000000",4,3,25,400,1,"Remove supernatant (WB4_2) and Add release buffer ",0,1,0);

Subroutine("C:¥Program Files¥TECAN¥EVOware¥database¥Scripts¥Diluter_Dispense_Air.esc",0);

Aspirate(255,"Remove Excess liquid from Magnet Plate Wash Buffer Asp SLOW","170","170","170","170","170","170","170","170",0,0,0,0,21,1,1,"0C08ｯ1000000000000",1,"Remove supernatant (WB4_2) and Add release buffer ",0,1,0);

Dispense(255,"Clearing contents of TIP","170","170","170","170","170","170","170","170",0,0,0,0,28,2,1,"0108ｯ1",0,0);

Set_DITIs_Back(255,15,2,"0C08ｯ1000000000000",1,"Remove supernatant (WB4_2) and Add release buffer ",0,1,0);

Comment("Release Mixing RNase ONE Buffer35ul");

Comment("Change Release buffer dispense position (-1 --> 0 mm) 6th Apr Kojima");

PickUp_DITIs2(255,2,1,"0C08000000pｯ000000",0,"DiTi 200 ul EtOH USE",0);

Aspirate(255,"ROBuffer","40","40","40","40","40","40","40","40",0,0,0,0,29,0,1,"0108ｯ1",0,0);

Dispense(255,"SD Water free dispense","40","40","40","40","40","40","40","40",0,0,0,0,21,1,1,"0C08ｯ1000000000000",1,"Remove supernatant (WB4_2) and Add release buffer ",0,1,0);

Set_DITIs_Back(255,2,1,"0C08000000pｯ000000",0,0);

EndLoop();

GroupEnd();

Group("(6) Release");

Comment("Move from Magnet --> Magnet Shift --> RT position");

Vector("SAFE <> Magnet","21","2",0,1,0,1,1,0);

Vector("MAGNET SHIFTING","21","2",0,0,2,2,1,0);

StartTimer("1");

WaitTimer("1","30");

Vector("MAGNET SHIFTING","21","2",1,0,2,2,1,0);

Vector("RT Assay 1 <> SAFE","21","1",0,1,2,0,1,0);

Vector("RT Assay 1 <> PUSHdown","21","1",0,1,0,0,1,0);

Vector("Roma FAR RIGHT","35","5",0,1,2,2,0,0);

BeginLoop("NC","Mix the sample and release Buffer_1");

Wash(255,1,1,1,0,"2",500,"1",500,10,70,30,1,0,1000,0);

GetDITI2(255,"DiTi 200 ul",0,0,10,70);

Aspirate(255,"Aspirate Low Volume Liquid Level","5","5","5","5","5","5","5","5",0,0,0,0,21,0,1,"0C08ｯ1000000000000",1,"Mix the sample and release Buffer_1",0,1,0);

Mix(255,"Mixing Ampure inside Plate","24","24","24","24","24","24","24","24",0,0,0,0,21,0,1,"0C08ｯ1000000000000",10,1,"Mix the sample and release Buffer_1",0,1,0);

Dispense(255,"Low Volume wet contact dispense with tracking HIGHER Liq Positn","5","5","5","5","5","5","5","5",0,0,0,0,21,0,1,"0C08ｯ1000000000000",1,"Mix the sample and release Buffer_1",0,1,0);

Detect_Liquid(255,"Mixing Ampure inside Plate Large Volume MIX",21,0,1,"0C08ｯ1000000000000",1,"Mix the sample and release Buffer_1",0,1,0);

DropDITI(255,2,2,10,70,0);

EndLoop();

FACTS("ThermalCycler","ThermalCycler_OpenLid","1","0","");

Comment("Move RT Assay 1 --> Cycler 95 degrees 5mins");

Vector("RT Assay 1 <> SAFE","21","1",0,1,0,1,1,0);

Vector("SAFE <> Cycler","45","1",0,1,2,0,1,0);

FACTS("ThermalCycler","ThermalCycler_CloseLid","1,0","0","");

IfThen("TEST_MODE",0,"0");

StartTimer("1");

WaitTimer("1","10");

Else();

StartTimer("1");

FACTS("ThermalCycler","ThermalCycler_RunBlock","1,REL,BLOCK,ON,300","0","");

WaitTimer("1","405");

EndIf();

FACTS("ThermalCycler","ThermalCycler_OpenLid","1","0","");

Comment("Move from Cycler --> Four Degrees Plate (wait 4 mins)");

Vector("SAFE <> Cycler","45","1",0,1,0,1,1,0);

Vector("FourDegreesPlate <-> SAFE","8","4",0,1,2,0,1,0);

Vector("FourDegreesPlate <->PUSHdown","8","4",0,1,0,0,0,0);

StartTimer("1");

WaitTimer("1","60");

Comment("Remove Script for mixing after heat shock 22th Feb Kojima");

Vector("FourDegreesPlate <-> SAFE","8","4",0,1,0,1,1,0);

Vector("SAFE <> Magnet","21","2",0,1,2,0,1,0);

Vector("SAFE <> PUSH down Magnet","21","2",0,1,0,0,0,0);

StartTimer("1");

Comment("10.25 Stand(Position) was changed");

Notification(1,"EVO Team","Confirmation:Plate stand: EVO 1st Information","Check Plate!",0);

Vector("ExtractedLiquid <> SAFE","35","3",0,1,0,1,1,0);

Vector("FourDegreesPlate <-> SAFE","8","4",0,1,2,0,1,0);

Vector("FourDegreesPlate <->PUSHdown","8","4",0,1,0,0,1,0);

Vector("FourDegreesPlate <-> SAFE","8","4",0,0,0,1,1,0);

Vector("FourDegreesPlate <-> SAFE","8","4",1,0,0,2,1,0);

ROMA(2,80,75,0,0,0,150,1,0);

Comment("10.25 Stand(Position) was changed");

Comment("REPLACE DITI BOX 1");

Vector("Transfer Diti 200 @ Cooling Carrier","8","1",0,1,0,1,1,0);

Vector("Transfer Diti Slot A","10","1",0,1,2,0,1,0);

Vector("Transfer Diti 200 @ Cooling Carrier","8","2",0,1,0,1,1,0);

Vector("Transfer Diti Slot A","10","1",0,1,2,0,1,0);

Vector("Transfer Diti Slot B","10","1",0,1,0,1,1,0);

Vector("Transfer Diti 200 @ Cooling Carrier","8","1",0,1,2,0,1,0);

Vector("Transfer Diti Slot B","10","1",0,1,0,1,1,0);

Vector("Transfer Diti 200 @ Cooling Carrier","8","2",0,1,2,0,1,0);

Vector("Roma FAR RIGHT","35","5",0,1,2,2,0,0);

Set_DITI_Counter2("DiTi 200 ul","8","1","1",0);

Comment("END REPLACE DITI BOX");

WaitTimer("1","5 * 60");

Comment("REPLACE DITI BOX 2");

Comment("Add new plate @ Extracted Liquid 1");

BeginLoop("NC","Transfer the supernatant to new plate and Add release buffer_2");

Wash(255,1,1,1,0,"2",500,"1",500,10,70,30,1,0,1000,0);

GetDITI2(255,"DiTi 200 ul",0,0,10,70);

Aspirate(255,"Left-over","30","30","30","30","30","30","30","30",0,0,0,0,21,1,1,"0C08ｯ1000000000000",1,"Transfer the supernatant to new plate and Add release buffer_2",0,1,0);

Dispense(255,"AMpure Collection","30","30","30","30","30","30","30","30",0,0,0,0,8,3,1,"0C08ｯ1000000000000",1,"Transfer the supernatant to new plate and Add release buffer_2",0,1,0);

Detect_Liquid(255,"Water free dispense",8,3,1,"0C08ｯ1000000000000",1,"Transfer the supernatant to new plate and Add release buffer_2",0,1,0);

DropDITI(255,2,2,10,70,0);

Comment("Release buffer 25ul");

Comment("Change Release buffer dispense position (-1 --> 0 mm) 6th Apr Kojima");

Wash(255,1,1,1,0,"2",500,"1",500,10,70,30,1,0,1000,0);

PickUp_DITIs2(255,2,1,"0C08000000pｯ000000",0,"DiTi 200 ul EtOH USE",0);

Aspirate(255,"ROBuffer","25","25","25","25","25","25","25","25",0,0,0,0,29,0,1,"0108ｯ1",0,0);

Dispense(255,"SD Water free dispense","25","25","25","25","25","25","25","25",0,0,0,0,21,1,1,"0C08ｯ1000000000000",1,"Transfer the supernatant to new plate and Add release buffer_2",0,1,0);

Set_DITIs_Back(255,2,1,"0C08000000pｯ000000",0,0);

EndLoop();

Comment("Move from Magnet --> Shift position --> RT position");

Vector("SAFE <> Magnet","21","2",0,1,0,1,1,0);

Vector("MAGNET SHIFTING","21","2",0,0,2,2,1,0);

StartTimer("1");

WaitTimer("1","30");

Vector("MAGNET SHIFTING","21","2",1,0,2,2,1,0);

Vector("RT Assay 1 <> SAFE","21","1",0,1,2,0,1,0);

Vector("RT Assay 1 <> PUSHdown","21","1",0,1,0,0,1,0);

Vector("Roma FAR RIGHT","35","5",0,1,2,2,0,0);

Comment("Mix release buffer 25ul ");

BeginLoop("NC","Mix the sample and release buffer_2");

Wash(255,1,1,1,0,"2",500,"1",500,10,70,30,1,0,1000,0);

GetDITI2(255,"DiTi 200 ul",0,0,10,70);

Aspirate(255,"Aspirate Low Volume Liquid Level","5","5","5","5","5","5","5","5",0,0,0,0,21,0,1,"0C08ｯ1000000000000",1,"Mix the sample and release buffer_2",0,1,0);

Mix(255,"Mixing Ampure inside Plate Slower","21","21","21","21","21","21","21","21",0,0,0,0,21,0,1,"0C08ｯ1000000000000",10,1,"Mix the sample and release buffer_2",0,1,0);

Dispense(255,"Low Volume wet contact dispense with tracking HIGHER Liq Positn","5","5","5","5","5","5","5","5",0,0,0,0,21,0,1,"0C08ｯ1000000000000",1,"Mix the sample and release buffer_2",0,1,0);

Detect_Liquid(255,"Mixing Ampure inside Plate Large Volume MIX",21,0,1,"0C08ｯ1000000000000",1,"Mix the sample and release buffer_2",0,1,0);

DropDITI(255,2,2,10,70,0);

EndLoop();

Comment("RT Assay Area 1--> Magnet ( wait 5 mins )");

Vector("RT Assay 1 <> SAFE","21","1",0,1,0,1,1,0);

Vector("SAFE <> Magnet","21","2",0,1,2,0,1,0);

Vector("SAFE <> PUSH down Magnet","21","2",0,1,0,2,1,0);

Vector("Roma FAR RIGHT","35","5",0,1,2,2,0,0);

StartTimer("1");

Comment("REPLACE DITI BOX 2 ");

Vector("Transfer Diti 200 @ Cooling Carrier","8","1",0,1,0,1,1,0);

Vector("Transfer Diti Slot A","10","1",0,1,2,0,1,0);

Vector("Transfer Diti Slot B","10","1",0,1,0,1,0,0);

Vector("Transfer Diti 200 @ Cooling Carrier","8","1",0,1,2,0,1,0);

Vector("Roma FAR RIGHT","35","5",0,1,2,2,0,0);

Set_DITI_Counter2("DiTi 200 ul","8","1","1",0);

Comment("END REPLACE DITI BOX");

WaitTimer("1","5 * 60");

BeginLoop("NC","Transfer the supernatant to new plate_2");

Wash(255,1,1,1,0,"2",500,"1",500,10,70,30,1,0,1000,0);

GetDITI2(255,"DiTi 200 ul",0,0,10,70);

Aspirate(255,"Left-over","45","45","45","45","45","45","45","45",0,0,0,0,21,1,1,"0C08ｯ1000000000000",1,"Transfer the supernatant to new plate_2",0,1,0);

Dispense(255,"AMpure Collection","45","45","45","45","45","45","45","45",0,0,0,0,8,3,1,"0C08ｯ1000000000000",1,"Transfer the supernatant to new plate_2",0,1,0);

Detect_Liquid(255,"Water free dispense",8,3,1,"0C08ｯ1000000000000",1,"Transfer the supernatant to new plate_2",0,1,0);

DropDITI(255,2,2,10,70,0);

EndLoop();

If("1",0,"1","SKIP");

Vector("Transfer Diti 200 @ Cooling Carrier","8","1",0,1,0,1,1,0);

Vector("Transfer Diti Slot B","10","1",0,1,2,0,1,0);

Vector("Transfer Diti 200 @ Cooling Carrier","8","2",0,1,0,1,1,0);

Vector("Transfer Diti Slot B","10","1",0,1,2,0,1,0);

Vector("Transfer Diti 200 @ Cooling Carrier","21","3",0,1,0,1,1,0);

Vector("Transfer Diti Slot B","10","1",0,1,2,0,1,0);

Vector("Transfer Diti Slot C","10","1",0,1,0,1,1,0);

Vector("Transfer Diti 200 @ Cooling Carrier","8","1",0,1,2,0,1,0);

Vector("Transfer Diti Slot C","10","1",0,1,0,1,1,0);

Vector("Transfer Diti 200 @ Cooling Carrier","8","2",0,1,2,0,1,0);

Vector("Transfer Diti Slot C","10","1",0,1,0,1,1,0);

Vector("Transfer Diti 200 @ Cooling Carrier","21","3",0,1,2,0,1,0);

Comment("Remove Obsolete Magnet Plate");

Vector("SAFE <> Magnet","21","2",0,1,0,1,1,0);

Vector("Dispose OLD Magnet Plate","21","2",0,1,2,0,1,0);

Comment("SKIP");

ROMA(2,80,75,0,0,0,150,1,0);

GroupEnd();

Notification(0,"EVO Team","Assay finish","Assay finish",0);

**---END---**

**5. RNaseH_and_PURIFICATION**

**---START---**

3634539C

20110601_152808 Admin

Administrator

--{ RES }--

V;200

--{ CFG }--

999;219;32;

14;-1;30;8;-1;-1;-1;-1;-1;234;-1;-1;-1;-1;-1;-1;96;-1;-1;-1;-1;-1;34;-1;-1;-1;-1;-1;246;244;245;243;242;238;-1;-1;239;-1;-1;-1;-1;-1;-1;-1;-1;-1;-1;-1;-1;-1;-1;-1;-1;-1;-1;-1;-1;-1;-1;-1;-1;-1;-1;-1;-1;-1;-1;-1;-1;-1;-1;-1;-1;-1;-1;-1;-1;-1;-1;-1;-1;-1;-1;-1;-1;-1;-1;-1;-1;-1;-1;-1;-1;-1;-1;-1;-1;-1;-1;-1;

998;0;

998;3;Wash Station Cleaner shallow;Wash Station Waste;Wash Station Cleaner deep;

998;;;;

998;3;;DiTi 200 ul EtOH USE;DiTi Waste;

998;;Diti 200- EtOH USE;Waste;

998;0;

998;0;

998;0;

998;0;

998;0;

998;4;DiTi 200 ul;DiTi 200 ul;DiTi 200 ul;96 Well PCR Plate;

998;Diti 200 -A;Diti 200 -B;Diti 200 -C;Cold PCR plate;

998;0;

998;0;

998;0;

998;0;

998;0;

998;0;

998;3;DiTi 200 ul MIX1;;;

998;AMP1 and CAPT 1;;;

998;0;

998;0;

998;0;

998;0;

998;0;

998;3;96 Well RT PCR;96 Well MAGNET PCR;;

998;RT PCR plate;MAGNET PCR;;

998;0;

998;0;

998;0;

998;0;

998;0;

998;3;;;;

998;;;;

998;3;;;Trough 100ml;

998;;;E70;

998;3;;;;

998;;;;

998;3;;;;

998;;;;

998;3;Tube 8pos RT Amp 5;;Trough 100ml ET 2;

998;Labware1;;E100;

998;6;0.5 ml Eppendorf Tube Ox;;;;;;

998;RH+RHO;;;;;;

998;0;

998;0;

998;7;Trough 25ml SD;;;;96 Well Microplate HOT;;;

998;SD;;;;Extd Liquid 3 and 6;;;

998;0;

998;0;

998;0;

998;0;

998;0;

998;0;

998;0;

998;0;

998;0;

998;0;

998;0;

998;0;

998;0;

998;0;

998;0;

998;0;

998;0;

998;0;

998;0;

998;0;

998;0;

998;0;

998;0;

998;0;

998;0;

998;0;

998;0;

998;0;

998;0;

998;0;

998;0;

998;0;

998;0;

998;0;

998;0;

998;0;

998;0;

998;0;

998;0;

998;0;

998;0;

998;0;

998;0;

998;0;

998;0;

998;0;

998;0;

998;0;

998;0;

998;0;

998;0;

998;0;

998;0;

998;0;

998;0;

998;0;

998;0;

998;0;

998;0;

998;0;

998;0;

998;0;

998;0;

998;2;

998;86;41;

998;241;10;

998;5;

998;4;0;System;

998;0;3;Magellan;

998;0;0;Thermal Cycler;

998;0;1;Hotel 4Pos DeepWell;

998;0;2;Hotel 2Pos DitiRack 1;

998;3;

998;236;96 Well PCR Plate;

998;86;96 Well PCR Plate;

998;241;DiTi 200 ul;

998;1;

998;1;

998;45;

998;41;

998;10;

996;0;0;

--{ RPG }--

Notification(1,"EVO Team","Error: EVO 1st Information","Error",1);

Comment("Mixing step of Enzyme was changed.Oct 19th Tera");

Comment("50ul Diti has been applied, Oct 7th 2010 Tera");

Comment("Change the name of Loop 23th Feb Kojima");

Comment("Change script (volume main) 23th Feb Kojima");

Comment("Renew the script of AMPure mixing 1st Mar Kojima");

Variable(TEST_MODE,"1",1,"TEST=0, REAL SUMPLE=1",1,0.000000,1.000000,0,2,1,0);

Variable(NC,"12",1,"How many lane？",1,1.000000,12.000000,0,2,1,0);

Set_DITI_Counter2("DiTi 200 ul","8","1","1",0);

Comment("***Start Position : Cooling Rack***");

Group("(7) RNase H");

Comment("RNaseH + RHaseONE 5ul");

Comment("Modified 10.19.2010");

BeginLoop("NC","Add RNaseH mixture to sample ");

Wash(255,1,1,1,0,"2",500,"1",500,10,70,30,1,0,1000,0);

GetDITI2(255,"DiTi 200 ul",1,0,10,70);

Aspirate(255,"Clearing BEAD contents in TIP_RNaseH&ONE2","5","5","5","5","5","5","5","5",0,0,0,0,32,0,1,"0108ｯ1",0,0);

Mix(255,"Enzyme Liquid Level","7.5","7.5","7.5","7.5","7.5","7.5","7.5","7.5",0,0,0,0,32,0,1,"0108ｯ1",2,0,0);

Aspirate(255,"RNaseH Low Volume 5ul aspiration and dispense Liquid Surface2","5","5","5","5","5","5","5","5",0,0,0,0,32,0,1,"0108ｯ1",0,0);

Dispense(255,"RNaseH Low Volume 5ul aspiration and dispense Liquid Surface2","5","5","5","5","5","5","5","5",0,0,0,0,8,3,1,"0C08ｯ1000000000000",1,"Add RNaseH mixture to sample ",0,1,0);

Aspirate(255,"Aspiration Small Vol","3","3","3","3","3","3","3","3",0,0,0,0,8,3,1,"0C08ｯ1000000000000",1,"Add RNaseH mixture to sample ",0,1,0);

BeginLoop("5","Sample mixing");

Aspirate(255,"Mixing RNaseH&ONE inside Plate Slower","32","32","32","32","32","32","32","32",0,0,0,0,8,3,1,"0C08ｯ1000000000000",1,"Add RNaseH mixture to sample ",0,1,0);

Dispense(255,"Mixing RNaseH&ONE inside Plate Slower","32","32","32","32","32","32","32","32",0,0,0,0,8,3,1,"0C08ｯ1000000000000",1,"Add RNaseH mixture to sample ",0,1,0);

EndLoop();

Aspirate(255,"Mixing RNaseH&ONE inside Plate Slower","32","32","32","32","32","32","32","32",0,0,0,0,8,3,1,"0C08ｯ1000000000000",1,"Add RNaseH mixture to sample ",0,1,0);

Dispense(255,"Mixing RNaseH&ONE inside Plate Slower2","32","32","32","32","32","32","32","32",0,0,0,0,8,3,1,"0C08ｯ1000000000000",1,"Add RNaseH mixture to sample ",0,1,0);

Dispense(255,"Aspiration Small Vol","3","3","3","3","3","3","3","3",0,0,0,0,8,3,1,"0C08ｯ1000000000000",1,"Add RNaseH mixture to sample ",0,1,0);

StartTimer("1");

WaitTimer("1","2");

Dispense(255,"Blow out slow_RNaseH&ONE","5","5","5","5","5","5","5","5",0,0,0,0,8,3,1,"0C08ｯ1000000000000",1,"Add RNaseH mixture to sample ",0,1,0);

Aspirate(255,"Clearing BEAD contents in TIP","25","25","25","25","25","25","25","25",0,0,0,0,8,3,1,"0C08ｯ1000000000000",1,"Add RNaseH mixture to sample ",0,1,0);

Dispense(255,"Clearing BEAD contents in TIP_Z-max -9","25","25","25","25","25","25","25","25",0,0,0,0,8,3,1,"0C08ｯ1000000000000",1,"Add RNaseH mixture to sample ",0,1,0);

Detect_Liquid(255,"Mixing Beads with Sample small fast",8,3,1,"0C08ｯ1000000000000",1,"Add RNaseH mixture to sample ",0,1,0);

DropDITI(255,2,2,10,70,0);

EndLoop();

Comment("End Modified 10.19.2010");

Comment("RH + RHO incubation @ 37 degrees for 15 mins");

Comment("Thermal Cycler");

FACTS("ThermalCycler","ThermalCycler_OpenLid","1","0","");

Vector("FourDegreesPlate <-> SAFE","8","4",0,1,0,1,0,0);

Vector("SAFE <> Cycler","45","1",0,1,2,0,0,0);

Vector("SAFE <> Cycler","45","1",0,0,0,1,0,0);

Vector("SAFE <> Cycler","45","1",1,0,0,2,0,0);

FACTS("ThermalCycler","ThermalCycler_CloseLid","1,0","0","");

StartTimer("1");

FACTS("ThermalCycler","ThermalCycler_RunBlock","1,37-15MIN,BLOCK,ON,900","0","");

WaitTimer("1","920");

FACTS("ThermalCycler","ThermalCycler_OpenLid","1","0","");

Vector("SAFE <> Cycler","45","1",0,1,0,1,0,0);

Vector("RT Assay 1 <> SAFE","21","1",0,1,2,0,0,0);

Vector("RT Assay 1 <> PUSHdown","21","1",0,1,0,0,1,0);

ROMA(2,80,75,0,0,0,150,1,0);

Comment("Thermal Cycler End");

GroupEnd();

Comment("Renew the script of AMPure mixing 1st Mar Kojima");

Group("(7) AMPure purification");

Comment("Set LowDiti Eject position to High level");

Wash(255,1,1,1,0,"2",500,"1",500,10,70,30,1,0,1000,0);

PickUp_DITIs2(255,2,1,"0C08000000000ｮ3000",0,"DiTi 200 ul EtOH USE",0);

Aspirate(255,"Aspirate Low Volume Liquid Level","5","5","5","5","5","5","5","5",0,0,0,0,31,0,1,"0108ｯ1",0,0);

IfThen("TEST_MODE",0,"0");

Variable(A,"5",0,"",0,0.000000,0.000000,0,2,0,0);

Else();

Variable(A,"10",0,"",0,0.000000,0.000000,0,2,0,0);

EndIf();

BeginLoop("A","Mix AMPure beads");

Aspirate(255,"Mixing for AMpure in Trough close to Z-max","180","180","180","180","180","180","180","180",0,0,0,0,31,0,1,"0108ｯ1",0,0);

Dispense(255,"Mixing AMPure dropplets high position","180","180","180","180","180","180","180","180",0,0,0,0,31,0,1,"0108ｯ1",0,0);

Mix(255,"Mixing for AMpure in Trough close to Z-max","150","150","150","150","150","150","150","150",0,0,0,0,31,0,1,"0108ｯ1",3,0,0);

EndLoop();

Dispense(255,"Low Volume wet contact dispense with tracking HIGHER Liq Positn","5","5","5","5","5","5","5","5",0,0,0,0,31,0,1,"0108ｯ1",0,0);

Detect_Liquid(255,"Contact dispense with tracking HIGH Liq Positn",31,0,1,"0108ｯ1",0,0);

DropDITI(255,2,2,10,70,0);

BeginLoop("NC","Add AMPure beads to sample and mixing ");

Wash(255,1,1,1,0,"2",500,"1",500,10,70,30,1,0,1000,0);

Subroutine("C:¥Program Files¥TECAN¥EVOware¥database¥Scripts¥Diluter_Aspirate_Air.esc",0);

PickUp_DITIs2(255,15,0,"0C08ｯ1000000000000",1,"Add AMPure beads to sample and mixing ",0,1,"DiTi 200 ul MIX1",0);

MoveLiha(255,15,0,1,"0C08ｯ1000000000000",4,3,25,400,1,"Add AMPure beads to sample and mixing ",0,1,0);

Subroutine("C:¥Program Files¥TECAN¥EVOware¥database¥Scripts¥Diluter_Dispense_Air.esc",0);

BeginLoop("3","AMPure beads mixing");

Aspirate(255,"Mixing for AMpure in Trough close to Z-max","80","80","80","80","80","80","80","80",0,0,0,0,31,0,1,"0108ｯ1",0,0);

Dispense(255,"Mixing AMPure dropplets high position","80","80","80","80","80","80","80","80",0,0,0,0,31,0,1,"0108ｯ1",0,0);

Mix(255,"Mixing for AMpure in Trough close to Z-max","80","80","80","80","80","80","80","80",0,0,0,0,31,0,1,"0108ｯ1",2,0,0);

EndLoop();

Aspirate(255,"AMPure and Beads","126","126","126","126","126","126","126","126",0,0,0,0,31,0,1,"0108ｯ1",0,0);

Detect_Liquid(255,"AMPure and Beads",31,0,1,"0108ｯ1",0,0);

Dispense(255,"AMPure and Beads","126","126","126","126","126","126","126","126",0,0,0,0,21,0,1,"0C08ｯ1000000000000",1,"Add AMPure beads to sample and mixing ",0,1,0);

Detect_Liquid(255,"AMPure and Beads",21,0,1,"0C08ｯ1000000000000",1,"Add AMPure beads to sample and mixing ",0,1,0);

Comment("Renew the script of AMPure mixing 1st Mar Kojima");

Aspirate(255,"Aspirate 2ul Liquid Level","5","5","5","5","5","5","5","5",0,0,0,0,21,0,1,"0C08ｯ1000000000000",1,"Add AMPure beads to sample and mixing ",0,1,0);

Mix(255,"Mixing 4th Ampure inside Plate","140","140","140","140","140","140","140","140",0,0,0,0,21,0,1,"0C08ｯ1000000000000",5,1,"Add AMPure beads to sample and mixing ",0,1,0);

IfThen("TEST_MODE",0,"0");

Variable(A,"3",0,"",0,0.000000,0.000000,0,2,0,0);

Else();

Variable(A,"5",0,"",0,0.000000,0.000000,0,2,0,0);

EndIf();

BeginLoop("A","AMPure beads and Sample mixing");

Aspirate(255,"Mixing 4th Ampure inside Plate Large Volume MIX","140","140","140","140","140","140","140","140",0,0,0,0,21,0,1,"0C08ｯ1000000000000",1,"Add AMPure beads to sample and mixing ",0,1,0);

Dispense(255,"Mixing 4th Ampure inside Plate Large Volume MIX","140","140","140","140","140","140","140","140",0,0,0,0,21,0,1,"0C08ｯ1000000000000",1,"Add AMPure beads to sample and mixing ",0,1,0);

EndLoop();

Dispense(255,"Mixing Beads with Sample small volume aspirate","5","5","5","5","5","5","5","5",0,0,0,0,21,0,1,"0C08ｯ1000000000000",1,"Add AMPure beads to sample and mixing ",0,1,0);

Detect_Liquid(255,"Mixing Ampure inside Plate Large Volume MIX",21,0,1,"0C08ｯ1000000000000",1,"Add AMPure beads to sample and mixing ",0,1,0);

Aspirate(255,"Clearing BEAD contents in TIP","20","20","20","20","20","20","20","20",0,0,0,0,21,0,1,"0C08ｯ1000000000000",1,"Add AMPure beads to sample and mixing ",0,1,0);

Dispense(255,"Clearing BEAD contents in TIP Zmax -17","20","20","20","20","20","20","20","20",0,0,0,0,21,0,1,"0C08ｯ1000000000000",1,"Add AMPure beads to sample and mixing ",0,1,0);

Set_DITIs_Back(255,15,0,"0C08ｯ1000000000000",1,"Add AMPure beads to sample and mixing ",0,1,0);

EndLoop();

Comment("Remove script of 'Mix the AMPure beads and Sample 3 loops ' 1st Mar Kojima");

StartTimer("1");

WaitTimer("1","30 * 60");

Comment(" Move RT Assay Area 1 --> Magnet ( wait 5 mins )");

Vector("Roma FAR RIGHT","35","5",0,1,2,2,0,0);

Vector("RT Assay 1 <> SAFE","21","1",0,1,0,1,1,0);

Vector("SAFE <> Magnet","21","2",0,1,2,0,1,0);

Vector("SAFE <> PUSH down Magnet","21","2",0,1,0,2,1,0);

Vector("Roma FAR RIGHT","35","5",0,1,2,2,1,0);

Comment("Wait for 10 mins");

StartTimer("1");

WaitTimer("1","10 * 60");

Group("Remove supernatant and add 100% EtOH");

Vector("ET 2 LID <> SAFE","31","3",0,1,0,1,1,0);

BeginLoop("NC","Remove supernatant and add 100% EtOH");

Wash(255,1,1,1,0,"2",500,"4.0",500,10,70,30,1,0,1000,0);

Subroutine("C:¥Program Files¥TECAN¥EVOware¥database¥Scripts¥Diluter_Aspirate_Air.esc",0);

PickUp_DITIs2(255,15,0,"0C08ｯ1000000000000",1,"Remove supernatant and add 100% EtOH",0,1,"DiTi 200 ul MIX1",0);

MoveLiha(255,15,0,1,"0C08ｯ1000000000000",4,3,25,400,1,"Remove supernatant and add 100% EtOH",0,1,0);

Subroutine("C:¥Program Files¥TECAN¥EVOware¥database¥Scripts¥Diluter_Dispense_Air.esc",0);

Aspirate(255,"Remove Supernatant","136","136","136","136","136","136","136","136",0,0,0,0,21,1,1,"0C08ｯ1000000000000",1,"Remove supernatant and add 100% EtOH",0,1,0);

Dispense(255,"Clearing contents of TIP","136","136","136","136","136","136","136","136",0,0,0,0,1,1,1,"0108ｯ1",0,0);

Set_DITIs_Back(255,15,0,"0C08ｯ1000000000000",1,"Remove supernatant and add 100% EtOH",0,1,0);

Wash(255,1,1,1,0,"2",500,"1",500,10,70,30,1,0,1000,0);

PickUp_DITIs2(255,2,1,"0C08ｯ1000000000000",0,"DiTi 200 ul EtOH USE",0);

Mix(255,"Solvent Mix","145","145","145","145","145","145","145","145",0,0,0,0,31,2,1,"0108ｯ1",2,0,0);

Aspirate(255,"Solvent for EtOH trough","140","140","140","140","140","140","140","140",0,0,0,0,31,2,1,"0108ｯ1",0,0);

Dispense(255,"Solvent","140","140","140","140","140","140","140","140",0,0,0,0,21,1,1,"0C08ｯ1000000000000",1,"Remove supernatant and add 100% EtOH",0,1,0);

Set_DITIs_Back(255,2,1,"0C08ｯ1000000000000",0,0);

EndLoop();

Vector("ET 2 LID <> SAFE","31","3",0,1,2,0,1,0);

GroupEnd();

Comment("Remove -1");

Comment("REMOVE Liquid from MAGNET plate ADD EtOH and REMOVE");

Vector("ET 1 LID <> SAFE","28","3",0,1,0,1,1,0);

Wash(255,1,1,1,0,"2",500,"1",500,10,70,30,1,0,1000,0);

BeginLoop("NC","Wash the AMPure beads and Add water for sample elution");

Comment(" Remove");

Subroutine("C:¥Program Files¥TECAN¥EVOware¥database¥Scripts¥Diluter_Aspirate_Air.esc",0);

PickUp_DITIs2(255,15,0,"0C08ｯ1000000000000",1,"Wash the AMPure beads and Add water for sample elution",0,1,"DiTi 200 ul MIX1",0);

MoveLiha(255,15,0,1,"0C08ｯ1000000000000",4,3,25,400,1,"Wash the AMPure beads and Add water for sample elution",0,1,0);

Subroutine("C:¥Program Files¥TECAN¥EVOware¥database¥Scripts¥Diluter_Dispense_Air.esc",0);

Aspirate(255,"Aspirate EtOH Low Volume Liquid Level","70","70","70","70","70","70","70","70",0,0,0,0,21,1,1,"0C08ｯ1000000000000",1,"Wash the AMPure beads and Add water for sample elution",0,1,0);

Dispense(255,"Clearing contents of TIP","70","70","70","70","70","70","70","70",0,0,0,0,1,1,1,"0108ｯ1",0,0);

Aspirate(255,"Remove Excess liquid from Magnet Plate","110","110","110","110","110","110","110","110",0,0,0,0,21,1,1,"0C08ｯ1000000000000",1,"Wash the AMPure beads and Add water for sample elution",0,1,0);

Dispense(255,"Clearing contents of TIP","110","110","110","110","110","110","110","110",0,0,0,0,1,1,1,"0108ｯ1",0,0);

Set_DITIs_Back(255,15,0,"0C08ｯ1000000000000",1,"Wash the AMPure beads and Add water for sample elution",0,1,0);

Wash(255,1,1,1,0,"2",500,"1",500,10,70,30,1,0,1000,0);

Comment("EtOH 200ul -1");

PickUp_DITIs2(255,2,1,"0C08ｯ1000000000000",0,"DiTi 200 ul EtOH USE",0);

Mix(255,"Solvent Mix","100","100","100","100","100","100","100","100",0,0,0,0,28,2,1,"0108ｯ1",2,0,0);

Aspirate(255,"Solvent for EtOH trough","100","100","100","100","100","100","100","100",0,0,0,0,28,2,1,"0108ｯ1",0,0);

Dispense(255,"Solvent","100","100","100","100","100","100","100","100",0,0,0,0,21,1,1,"0C08ｯ1000000000000",1,"Wash the AMPure beads and Add water for sample elution",0,1,0);

Aspirate(255,"Solvent for EtOH trough","100","100","100","100","100","100","100","100",0,0,0,0,28,2,1,"0108ｯ1",0,0);

Dispense(255,"Solvent","100","100","100","100","100","100","100","100",0,0,0,0,21,1,1,"0C08ｯ1000000000000",1,"Wash the AMPure beads and Add water for sample elution",0,1,0);

Detect_Liquid(255,"Solvent",28,2,1,"0108ｯ1",0,0);

Set_DITIs_Back(255,2,1,"0C08ｯ1000000000000",0,0);

Wash(255,1,1,1,0,"2",500,"1",500,10,70,30,1,0,1000,0);

Subroutine("C:¥Program Files¥TECAN¥EVOware¥database¥Scripts¥Diluter_Aspirate_Air.esc",0);

PickUp_DITIs2(255,15,0,"0C08ｯ1000000000000",1,"Wash the AMPure beads and Add water for sample elution",0,1,"DiTi 200 ul MIX1",0);

MoveLiha(255,15,0,1,"0C08ｯ1000000000000",4,3,25,400,1,"Wash the AMPure beads and Add water for sample elution",0,1,0);

Subroutine("C:¥Program Files¥TECAN¥EVOware¥database¥Scripts¥Diluter_Dispense_Air.esc",0);

Aspirate(255,"Aspirate EtOH Low Volume Liquid Level","120","120","120","120","120","120","120","120",0,0,0,0,21,1,1,"0C08ｯ1000000000000",1,"Wash the AMPure beads and Add water for sample elution",0,1,0);

Dispense(255,"Clearing contents of TIP","120","120","120","120","120","120","120","120",0,0,0,0,1,1,1,"0108ｯ1",0,0);

Aspirate(255,"Solvent without asp airgap at end","130","130","130","130","130","130","130","130",0,0,0,0,21,1,1,"0C08ｯ1000000000000",1,"Wash the AMPure beads and Add water for sample elution",0,1,0);

Dispense(255,"Clearing contents of TIP","130","130","130","130","130","130","130","130",0,0,0,0,1,1,1,"0108ｯ1",0,0);

Set_DITIs_Back(255,15,0,"0C08ｯ1000000000000",1,"Wash the AMPure beads and Add water for sample elution",0,1,0);

Wash(255,1,1,1,0,"2",500,"1",500,10,70,30,1,0,1000,0);

Comment("Pip EtOH 2");

PickUp_DITIs2(255,2,1,"0C08ｯ1000000000000",0,"DiTi 200 ul EtOH USE",0);

Aspirate(255,"Solvent for EtOH trough","100","100","100","100","100","100","100","100",0,0,0,0,28,2,1,"0108ｯ1",0,0);

Dispense(255,"Solvent","100","100","100","100","100","100","100","100",0,0,0,0,21,1,1,"0C08ｯ1000000000000",1,"Wash the AMPure beads and Add water for sample elution",0,1,0);

Aspirate(255,"Solvent for EtOH trough","100","100","100","100","100","100","100","100",0,0,0,0,28,2,1,"0108ｯ1",0,0);

Dispense(255,"Solvent","100","100","100","100","100","100","100","100",0,0,0,0,21,1,1,"0C08ｯ1000000000000",1,"Wash the AMPure beads and Add water for sample elution",0,1,0);

Detect_Liquid(255,"Solvent",28,2,1,"0108ｯ1",0,0);

Set_DITIs_Back(255,2,1,"0C08ｯ1000000000000",0,0);

Wash(255,1,1,1,0,"2",500,"1",500,10,70,30,1,0,1000,0);

Comment("Pip Top layer 2");

Subroutine("C:¥Program Files¥TECAN¥EVOware¥database¥Scripts¥Diluter_Aspirate_Air.esc",0);

PickUp_DITIs2(255,15,0,"0C08ｯ1000000000000",1,"Wash the AMPure beads and Add water for sample elution",0,1,"DiTi 200 ul MIX1",0);

MoveLiha(255,15,0,1,"0C08ｯ1000000000000",4,3,25,400,1,"Wash the AMPure beads and Add water for sample elution",0,1,0);

Subroutine("C:¥Program Files¥TECAN¥EVOware¥database¥Scripts¥Diluter_Dispense_Air.esc",0);

Aspirate(255,"Aspirate EtOH Low Volume Liquid Level","120","120","120","120","120","120","120","120",0,0,0,0,21,1,1,"0C08ｯ1000000000000",1,"Wash the AMPure beads and Add water for sample elution",0,1,0);

Dispense(255,"Clearing contents of TIP","120","120","120","120","120","120","120","120",0,0,0,0,1,1,1,"0108ｯ1",0,0);

Aspirate(255,"Solvent without asp airgap at end","70","70","70","70","70","70","70","70",0,0,0,0,21,1,1,"0C08ｯ1000000000000",1,"Wash the AMPure beads and Add water for sample elution",0,1,0);

Dispense(255,"Clearing contents of TIP","70","70","70","70","70","70","70","70",0,0,0,0,1,1,1,"0108ｯ1",0,0);

Aspirate(255,"Solvent","70","70","70","70","70","70","70","70",0,0,0,0,21,1,1,"0C08ｯ1000000000000",1,"Wash the AMPure beads and Add water for sample elution",0,1,0);

Dispense(255,"Clearing contents of TIP","70","70","70","70","70","70","70","70",0,0,0,0,1,1,1,"0108ｯ1",0,0);

Aspirate(255,"Clearing contents of TIP","100","100","100","100","100","100","100","100",0,0,0,0,1,1,1,"0108ｯ1",0,0);

Dispense(255,"Clearing contents of TIP variation","100","100","100","100","100","100","100","100",0,0,0,0,1,1,1,"0108ｯ1",0,0);

Set_DITIs_Back(255,15,0,"0C08ｯ1000000000000",1,"Wash the AMPure beads and Add water for sample elution",0,1,0);

Comment("Water 42ul");

Wash(255,1,1,1,0,"2",500,"1",500,10,70,30,1,0,1000,0);

PickUp_DITIs2(255,2,1,"0C0800ｬ70000000000",0,"DiTi 200 ul EtOH USE",0);

Aspirate(255,"SD Water free dispense","42","42","42","42","42","42","42","42",0,0,0,0,35,0,1,"0108ｯ1",0,0);

Dispense(255,"SD Water free dispense","42","42","42","42","42","42","42","42",0,0,0,0,21,1,1,"0C08ｯ1000000000000",1,"Wash the AMPure beads and Add water for sample elution",0,1,0);

Set_DITIs_Back(255,2,1,"0C0800ｬ70000000000",0,0);

EndLoop();

Vector("ET 1 LID <> SAFE","28","3",0,1,2,0,1,0);

Comment("Move from Magnet --> to SHIFT position");

Vector("SAFE <> Magnet","21","2",0,1,0,1,1,0);

Vector("MAGNET SHIFTING","21","2",0,0,2,2,1,0);

StartTimer("1");

WaitTimer("1","60");

Vector("MAGNET SHIFTING","21","2",1,0,2,2,1,0);

Vector("RT Assay 1 <> SAFE","21","1",0,1,2,0,1,0);

Vector("RT Assay 1 <> PUSHdown","21","1",0,1,0,0,1,0);

Vector("Roma FAR RIGHT","35","5",0,1,2,2,0,0);

Comment("Mix 42 SD on RT");

Comment("Move from Magnet --> RT Assay Area 1 ( wait 5 mins)");

Notification(1,"EVO Team","Confirmation:Plate stand: EVO 1st Information","Check Plate! About 30 min before",0);

BeginLoop("NC","Mix the sample for elution");

Wash(255,1,1,1,0,"2",500,"1",500,10,70,30,1,0,1000,0);

GetDITI2(255,"DiTi 200 ul",1,0,10,70);

Aspirate(255,"Mixing Beads with Sample small volume aspirate","5","5","5","5","5","5","5","5",0,0,0,0,21,0,1,"0C08ｯ1000000000000",1,"Mix the sample for elution",0,1,0);

IfThen("TEST_MODE",0,"0");

Variable(D,"5",0,"",0,0.000000,0.000000,0,2,0,0);

Else();

Variable(D,"30",0,"",0,0.000000,0.000000,0,2,0,0);

EndIf();

BeginLoop("D","Sample mixing");

Aspirate(255,"Mixing Beads with Sample small fast","25","25","25","25","25","25","25","25",0,0,0,0,21,0,1,"0C08ｯ1000000000000",1,"Mix the sample for elution",0,1,0);

Dispense(255,"Mixing Beads with Sample small fast","25","25","25","25","25","25","25","25",0,0,0,0,21,0,1,"0C08ｯ1000000000000",1,"Mix the sample for elution",0,1,0);

EndLoop();

Dispense(255,"Mixing Beads with Sample small volume aspirate","5","5","5","5","5","5","5","5",0,0,0,0,21,0,1,"0C08ｯ1000000000000",1,"Mix the sample for elution",0,1,0);

Detect_Liquid(255,"Mixing Beads with Sample small fast",21,0,1,"0C08ｯ1000000000000",1,"Mix the sample for elution",0,1,0);

Aspirate(255,"Clearing BEAD contents in TIP","20","20","20","20","20","20","20","20",0,0,0,0,21,0,1,"0C08ｯ1000000000000",1,"Mix the sample for elution",0,1,0);

Dispense(255,"Clearing BEAD contents in TIP_Z-max -9","20","20","20","20","20","20","20","20",0,0,0,0,21,0,1,"0C08ｯ1000000000000",1,"Mix the sample for elution",0,1,0);

Detect_Liquid(255,"Mixing Beads with Sample small fast",21,0,1,"0C08ｯ1000000000000",1,"Mix the sample for elution",0,1,0);

DropDITI(255,2,2,10,70,0);

EndLoop();

Wash(255,1,1,1,0,"2",500,"1",500,10,70,30,1,0,1000,0);

FACTS("ThermalCycler","ThermalCycler_OpenLid","1","0","");

Vector("RT Assay 1 <> SAFE","21","1",0,1,0,1,1,0);

Vector("SAFE <> Cycler","45","1",0,1,2,0,1,0);

FACTS("ThermalCycler","ThermalCycler_CloseLid","1,0","0","");

Comment("10.25 Stand(Position) was changed");

Notification(1,"EVO Team","Confirmation:Plate stand: EVO 1st Information","Check Plate!",0);

Vector("ExtractedLiquid <> SAFE","35","5",0,1,0,1,1,0);

Vector("RT Assay 1 <> SAFE","21","1",0,1,2,0,1,0);

Vector("RT Assay 1 <> PUSHdown","21","1",0,1,0,0,1,0);

Vector("RT Assay 1 <> SAFE","21","1",0,0,0,1,1,0);

Vector("RT Assay 1 <> SAFE","21","1",1,0,0,2,1,0);

ROMA(2,80,75,0,0,0,150,1,0);

Comment("10.25 Stand(Position) was changed");

IfThen("TEST_MODE",0,"0");

StartTimer("1");

WaitTimer("1","10");

Else();

StartTimer("1");

FACTS("ThermalCycler","ThermalCycler_RunBlock","1,SD42,BLOCK,ON,300","0","");

WaitTimer("1","315");

EndIf();

FACTS("ThermalCycler","ThermalCycler_OpenLid","1","0","");

Vector("SAFE <> Cycler","45","1",0,1,0,1,1,0);

Vector("SAFE <> Magnet","21","2",0,1,2,0,1,0);

Vector("SAFE <> PUSH down Magnet","21","2",0,1,0,2,1,0);

Comment("Move RT Assay Area 1 --> Magnet ( wait 5 mins )");

Vector("Roma FAR RIGHT","35","5",0,1,2,2,0,0);

StartTimer("1");

WaitTimer("1","10 * 60");

Comment("Remove -4");

BeginLoop("NC","Transfer the sample to New plate");

Wash(255,1,1,1,0,"2",500,"1",500,10,70,30,1,0,1000,0);

GetDITI2(255,"DiTi 200 ul",0,0,10,70);

Aspirate(255,"Left-over","47","47","47","47","47","47","47","47",0,0,0,0,21,1,1,"0C08ｯ1000000000000",1,"Transfer the sample to New plate",0,1,0);

Dispense(255,"AMpure Collection","47","47","47","47","47","47","47","47",0,0,0,0,21,0,1,"0C08ｯ1000000000000",1,"Transfer the sample to New plate",0,1,0);

DropDITI(255,2,2,10,70,0);

EndLoop();

Comment("Set LowDiti Eject position back to Original");

Comment("37 Incubate 10min.");

Comment("10.25 Stand(Position) was changed");

Vector("RT Assay 1 <> SAFE","21","1",0,1,0,1,0,0);

Vector("ExtractedLiquid <> SAFE","35","5",0,1,2,0,1,0);

Vector("ExtractedLiquid <> PUSHdown","35","5",0,1,0,0,1,0);

ROMA(2,80,75,0,0,0,150,1,0);

Comment("10.25 Stand(Position) was changed");

StartTimer("1");

WaitTimer("1","10 * 60");

Vector("ExtractedLiquid <> SAFE","35","5",0,1,0,1,1,0);

Vector("FourDegreesPlate <-> SAFE","8","4",0,1,2,0,1,0);

ROMA(2,80,75,0,0,0,150,1,0);

If("1",0,"1","HERE");

Vector("Transfer Diti 200 @ Cooling Carrier","8","1",0,1,0,1,1,0);

Vector("Transfer Diti Slot C","10","1",0,1,2,0,1,0);

Vector("Transfer Diti 200 @ Cooling Carrier","8","2",0,1,0,1,1,0);

Vector("Transfer Diti Slot C","10","1",0,1,2,0,1,0);

Vector("Transfer Diti 200 @ Cooling Carrier","8","3",0,1,0,1,1,0);

Vector("Transfer Diti Slot C","10","1",0,1,2,0,1,0);

Vector("Transfer Diti Slot D","10","1",0,1,0,1,1,0);

Vector("Transfer Diti 200 @ Cooling Carrier","8","1",0,1,2,0,1,0);

Vector("Transfer Diti Slot D","10","1",0,1,0,1,1,0);

Vector("Transfer Diti 200 @ Cooling Carrier","8","2",0,1,2,0,1,0);

Vector("Transfer Diti Slot D","10","1",0,1,0,1,1,0);

Vector("Transfer Diti 200 @ Cooling Carrier","8","3",0,1,2,0,1,0);

Comment("Dispose Obsolete plate from Magnet");

Vector("SAFE <> Magnet","21","2",0,1,0,1,1,0);

Vector("Dispose OLD Magnet Plate","21","2",0,1,2,0,1,0);

Comment("HERE");

ROMA(2,80,75,0,0,0,150,1,0);

GroupEnd();

Notification(0,"EVO Team","Assay finish","Assay finish",0);

**---END---**

**6. RNaseONE_and_PURIFICATION**

**---START---**

E953EBC9

20110601_153339 Admin

Administrator

--{ RES }--

V;200

--{ CFG }--

999;219;32;

14;-1;30;8;-1;-1;-1;-1;-1;234;-1;-1;-1;-1;-1;-1;96;-1;-1;-1;-1;-1;34;-1;-1;-1;-1;-1;246;244;245;243;242;238;-1;-1;239;-1;-1;-1;-1;-1;-1;-1;-1;-1;-1;-1;-1;-1;-1;-1;-1;-1;-1;-1;-1;-1;-1;-1;-1;-1;-1;-1;-1;-1;-1;-1;-1;-1;-1;-1;-1;-1;-1;-1;-1;-1;-1;-1;-1;-1;-1;-1;-1;-1;-1;-1;-1;-1;-1;-1;-1;-1;-1;-1;-1;-1;-1;-1;

998;0;

998;3;Wash Station Cleaner shallow;Wash Station Waste;Wash Station Cleaner deep;

998;;;;

998;3;;DiTi 200 ul EtOH USE;DiTi Waste;

998;;Diti 200- EtOH USE;Waste;

998;0;

998;0;

998;0;

998;0;

998;0;

998;4;DiTi 200 ul;DiTi 200 ul;DiTi 50ul 2;96 Well PCR Plate;

998;Diti 200 -A;Diti 200 -B;DiTi 50ul;Cold PCR plate;

998;0;

998;0;

998;0;

998;0;

998;0;

998;0;

998;3;;DiTi 200 ul MIX1;;

998;;AMP1 and CAPT 1;;

998;0;

998;0;

998;0;

998;0;

998;0;

998;3;96 Well RT PCR;96 Well MAGNET PCR;;

998;RT PCR plate;MAGNET PCR;;

998;0;

998;0;

998;0;

998;0;

998;0;

998;3;;;;

998;;;;

998;3;;;Trough 100ml;

998;;;E1;

998;3;;;;

998;;;;

998;3;;;;

998;;;;

998;3;Tube 8pos RT Amp 6;;Trough 100ml ET 2;

998;Labware3;;E2;

998;6;;;0.5 ml Eppendorf Tube ROM;;;;

998;;;Rom;;;;

998;0;

998;0;

998;7;Trough 25ml SD;;;;96 Well Microplate HOT;;;

998;SD;;;;Labware4;;;

998;0;

998;0;

998;0;

998;0;

998;0;

998;0;

998;0;

998;0;

998;0;

998;0;

998;0;

998;0;

998;0;

998;0;

998;0;

998;0;

998;0;

998;0;

998;0;

998;0;

998;0;

998;0;

998;0;

998;0;

998;0;

998;0;

998;0;

998;0;

998;0;

998;0;

998;0;

998;0;

998;0;

998;0;

998;0;

998;0;

998;0;

998;0;

998;0;

998;0;

998;0;

998;0;

998;0;

998;0;

998;0;

998;0;

998;0;

998;0;

998;0;

998;0;

998;0;

998;0;

998;0;

998;0;

998;0;

998;0;

998;0;

998;0;

998;0;

998;0;

998;0;

998;0;

998;0;

998;2;

998;86;41;

998;241;10;

998;5;

998;4;0;System;

998;0;3;Magellan;

998;0;0;Thermal Cycler;

998;0;1;Hotel 4Pos DeepWell;

998;0;2;Hotel 2Pos DitiRack 1;

998;3;

998;236;96 Well PCR Plate;

998;86;96 Well PCR Plate;

998;241;DiTi 200 ul;

998;1;

998;1;

998;45;

998;41;

998;10;

996;0;0;

--{ RPG }--

Notification(1,"EVO Team","Error: EVO 1st Information","Error",1);

Comment("Mixing step of Enzyme was changed.Oct 19th Tera");

Comment("50ul Diti has been applied, Oct 7th 2010 Tera");

Comment("Change the name of Loop 23th Feb Kojima");

Comment("Change the Script (volume main) 23th Feb Kojima");

Comment("Renew the Script of AMPure 24th Feb Kojima ");

Comment("Change the name of Loop 2nd Mar Kojima");

Variable(TEST_MODE,"1",1,"TEST=0, REAL SUMPLE=1",1,0.000000,1.000000,0,2,1,0);

Variable(NC,"12",1,"How many lane？",1,1.000000,12.000000,0,2,1,0);

Vector("Roma FAR RIGHT","35","5",0,1,2,2,1,0);

Set_DITI_Counter2("DiTi 200 ul","8","1","1",0);

Set_DITI_Counter2("DiTi 50ul 2","8","3","1",0);

Command("C5SSP800,90,0",1,1,,,2,2,0);

Comment("***Start Position : Cooling Rack***");

Group("(8) RNase One");

Comment("RNase One mixture 5ul");

Comment("Modified 10.19.2010");

BeginLoop("NC","Add RNase One mixture to sample ");

Comment("Mixing step of Enzyme was changed.Oct 19th Tera");

Wash(255,1,1,1,0,"5",500,"1",500,10,70,30,1,0,1000,0);

GetDITI2(255,"DiTi 50ul 2",1,0,10,70);

Aspirate(255,"Clearing BEAD contents in TIP","5","5","5","5","5","5","5","5",0,0,0,0,32,2,1,"0108ｯ1",0,0);

Mix(255,"Enzyme Liquid Level","7","7","7","7","7","7","7","7",0,0,0,0,32,2,1,"0108ｯ1",2,0,0);

Aspirate(255,"ROM 5ul wet contact dispense2","5","5","5","5","5","5","5","5",0,0,0,0,32,2,1,"0108ｯ1",0,0);

Dispense(255,"ROM 5ul wet contact dispense2","5","5","5","5","5","5","5","5",0,0,0,0,8,3,1,"0C08ｯ1000000000000",1,"Add RNase One mixture to sample ",0,1,0);

Aspirate(255,"Aspiration Small Vol","3","3","3","3","3","3","3","3",0,0,0,0,8,3,1,"0C08ｯ1000000000000",1,"Add RNase One mixture to sample ",0,1,0);

BeginLoop("5","Sample mixing");

Aspirate(255,"Mixing RNaseONE inside Plate Slower","30","30","30","30","30","30","30","30",0,0,0,0,8,3,1,"0C08ｯ1000000000000",1,"Add RNase One mixture to sample ",0,1,0);

Dispense(255,"Mixing RNaseONE inside Plate Slower","30","30","30","30","30","30","30","30",0,0,0,0,8,3,1,"0C08ｯ1000000000000",1,"Add RNase One mixture to sample ",0,1,0);

EndLoop();

Aspirate(255,"Mixing RNaseONE inside Plate Slower","30","30","30","30","30","30","30","30",0,0,0,0,8,3,1,"0C08ｯ1000000000000",1,"Add RNase One mixture to sample ",0,1,0);

Dispense(255,"Mixing RNaseONE inside Plate Slower2","30","30","30","30","30","30","30","30",0,0,0,0,8,3,1,"0C08ｯ1000000000000",1,"Add RNase One mixture to sample ",0,1,0);

Dispense(255,"Aspiration Small Vol","3","3","3","3","3","3","3","3",0,0,0,0,8,3,1,"0C08ｯ1000000000000",1,"Add RNase One mixture to sample ",0,1,0);

StartTimer("1");

WaitTimer("1","2");

Dispense(255,"Blow out slow","5","5","5","5","5","5","5","5",0,0,0,0,8,3,1,"0C08ｯ1000000000000",1,"Add RNase One mixture to sample ",0,1,0);

Aspirate(255,"Clearing BEAD contents in TIP","25","25","25","25","25","25","25","25",0,0,0,0,8,3,1,"0C08ｯ1000000000000",1,"Add RNase One mixture to sample ",0,1,0);

Dispense(255,"Clearing BEAD contents in TIP","25","25","25","25","25","25","25","25",0,0,0,0,8,3,1,"0C08ｯ1000000000000",1,"Add RNase One mixture to sample ",0,1,0);

Detect_Liquid(255,"Detect liqid",8,3,1,"0C08ｯ1000000000000",1,"Add RNase One mixture to sample ",0,1,0);

DropDITI(255,2,2,10,70,0);

EndLoop();

Comment("End Modified 10.19.2010");

FACTS("ThermalCycler","ThermalCycler_OpenLid","1","0","");

Vector("FourDegreesPlate <-> SAFE","8","4",0,1,0,1,0,0);

Vector("SAFE <> Cycler","45","1",0,1,2,0,0,0);

Vector("SAFE <> Cycler","45","1",0,0,0,1,0,0);

Vector("SAFE <> Cycler","45","1",1,0,0,2,0,0);

FACTS("ThermalCycler","ThermalCycler_CloseLid","1,0","0","");

StartTimer("1");

FACTS("ThermalCycler","ThermalCycler_RunBlock1","37-30MIN,BLOCK,ON,1800","0","");

WaitTimer("1","1820");

FACTS("ThermalCycler","ThermalCycler_OpenLid","1","0","");

Vector("SAFE <> Cycler","45","1",0,1,0,1,0,0);

Vector("RT Assay 1 <> SAFE","21","1",0,1,2,0,0,0);

Vector("RT Assay 1 <> PUSHdown","21","1",0,1,0,0,1,0);

ROMA(2,80,75,0,0,0,150,1,0);

GroupEnd();

Comment("Renew the Script of AMPure 24th Feb Kojima ");

Group("(8) AMPure purification");

Comment("Set LowDiti Eject position to High level");

Wash(255,1,1,1,0,"5",500,"1",500,10,70,30,1,0,1000,0);

PickUp_DITIs2(255,2,1,"0C080000000000ｬ700",0,"DiTi 200 ul EtOH USE",0);

Aspirate(255,"Aspirate Low Volume Liquid Level","5","5","5","5","5","5","5","5",0,0,0,0,31,0,1,"0108ｯ1",0,0);

IfThen("TEST_MODE",0,"0");

Variable(A,"5",0,"",0,0.000000,0.000000,0,2,0,0);

Else();

Variable(A,"10",0,"",0,0.000000,0.000000,0,2,0,0);

EndIf();

BeginLoop("A","Mix AMPure beads");

Aspirate(255,"Mixing for AMpure in Trough close to Z-max","180","180","180","180","180","180","180","180",0,0,0,0,31,0,1,"0108ｯ1",0,0);

Dispense(255,"Mixing AMPure dropplets high position","180","180","180","180","180","180","180","180",0,0,0,0,31,0,1,"0108ｯ1",0,0);

Mix(255,"Mixing for AMpure in Trough close to Z-max","150","150","150","150","150","150","150","150",0,0,0,0,31,0,1,"0108ｯ1",3,0,0);

EndLoop();

Dispense(255,"Low Volume wet contact dispense with tracking HIGHER Liq Positn","5","5","5","5","5","5","5","5",0,0,0,0,31,0,1,"0108ｯ1",0,0);

Detect_Liquid(255,"Contact dispense with tracking HIGH Liq Positn",31,0,1,"0108ｯ1",0,0);

DropDITI(255,2,2,10,70,0);

BeginLoop("NC","Add AMPure beads to sample and mixing");

Wash(255,1,1,1,0,"2",500,"1",500,10,70,30,1,0,1000,0);

Subroutine("C:¥Program Files¥TECAN¥EVOware¥database¥Scripts¥Diluter_Aspirate_Air.esc",0);

PickUp_DITIs2(255,15,1,"0C08ｯ1000000000000",1,"Add AMPure beads to sample and mixing",0,1,"DiTi 200 ul MIX1",0);

MoveLiha(255,15,1,1,"0C08ｯ1000000000000",4,3,25,400,1,"Add AMPure beads to sample and mixing",0,1,0);

Subroutine("C:¥Program Files¥TECAN¥EVOware¥database¥Scripts¥Diluter_Dispense_Air.esc",0);

BeginLoop("3","AMPure beads mixing");

Aspirate(255,"Mixing for AMpure in Trough close to Z-max","80","80","80","80","80","80","80","80",0,0,0,0,31,0,1,"0108ｯ1",0,0);

Dispense(255,"Mixing AMPure dropplets high position","80","80","80","80","80","80","80","80",0,0,0,0,31,0,1,"0108ｯ1",0,0);

Mix(255,"Mixing for AMpure in Trough close to Z-max","80","80","80","80","80","80","80","80",0,0,0,0,31,0,1,"0108ｯ1",2,0,0);

EndLoop();

Aspirate(255,"AMPure and Beads","81","81","81","81","81","81","81","81",0,0,0,0,31,0,1,"0108ｯ1",0,0);

Detect_Liquid(255,"AMPure and Beads",31,0,1,"0108ｯ1",0,0);

Dispense(255,"AMPure and Beads","81","81","81","81","81","81","81","81",0,0,0,0,21,0,1,"0C08ｯ1000000000000",1,"Add AMPure beads to sample and mixing",0,1,0);

Detect_Liquid(255,"AMPure and Beads",21,0,1,"0C08ｯ1000000000000",1,"Add AMPure beads to sample and mixing",0,1,0);

Comment("Renew the Script of AMPure 24th Feb Kojima ");

Aspirate(255,"Aspirate 2ul Liquid Level","5","5","5","5","5","5","5","5",0,0,0,0,21,0,1,"0C08ｯ1000000000000",1,"Add AMPure beads to sample and mixing",0,1,0);

Mix(255,"Mixing 4th Ampure inside Plate","100","100","100","100","100","100","100","100",0,0,0,0,21,0,1,"0C08ｯ1000000000000",5,1,"Add AMPure beads to sample and mixing",0,1,0);

IfThen("TEST_MODE",0,"0");

Variable(A,"3",0,"",0,0.000000,0.000000,0,2,0,0);

Else();

Variable(A,"5",0,"",0,0.000000,0.000000,0,2,0,0);

EndIf();

BeginLoop("A","AMPure beads and Sample mixing");

Aspirate(255,"Mixing 5th Ampure inside Plate Large Volume MIX","100","100","100","100","100","100","100","100",0,0,0,0,21,0,1,"0C08ｯ1000000000000",1,"Add AMPure beads to sample and mixing",0,1,0);

Dispense(255,"Mixing 5th Ampure inside Plate Large Volume MIX","100","100","100","100","100","100","100","100",0,0,0,0,21,0,1,"0C08ｯ1000000000000",1,"Add AMPure beads to sample and mixing",0,1,0);

EndLoop();

Dispense(255,"Mixing Beads with Sample small volume aspirate","5","5","5","5","5","5","5","5",0,0,0,0,21,0,1,"0C08ｯ1000000000000",1,"Add AMPure beads to sample and mixing",0,1,0);

Detect_Liquid(255,"Mixing Ampure inside Plate Large Volume MIX",21,0,1,"0C08ｯ1000000000000",1,"Add AMPure beads to sample and mixing",0,1,0);

Aspirate(255,"Clearing BEAD contents in TIP","20","20","20","20","20","20","20","20",0,0,0,0,21,0,1,"0C08ｯ1000000000000",1,"Add AMPure beads to sample and mixing",0,1,0);

Dispense(255,"Clearing BEAD contents in TIP Zmax -17","20","20","20","20","20","20","20","20",0,0,0,0,21,0,1,"0C08ｯ1000000000000",1,"Add AMPure beads to sample and mixing",0,1,0);

Set_DITIs_Back(255,15,1,"0C08ｯ1000000000000",1,"Add AMPure beads to sample and mixing",0,1,0);

EndLoop();

Comment("Remove script of 'Mix the AMPure beads and Sample 3 loops ' 24th Feb Kojima");

StartTimer("1");

WaitTimer("1","30 * 60");

Comment(" Move RT Assay Area 1 --> Magnet ( wait 5 mins )");

Vector("RT Assay 1 <> SAFE","21","1",0,1,0,1,1,0);

Vector("SAFE <> Magnet","21","2",0,1,2,0,1,0);

Vector("SAFE <> PUSH down Magnet","21","2",0,1,0,2,1,0);

Vector("Roma FAR RIGHT","35","5",0,1,2,2,1,0);

Comment("Wait for 10 mins");

StartTimer("1");

WaitTimer("1","10 * 60");

Group("Remove supernatant and add 100% EtOH");

Vector("ET 2 LID <> SAFE","31","3",0,1,0,1,1,0);

BeginLoop("NC","Remove supernatant and add 100% EtOH");

Wash(255,1,1,1,0,"2",500,"1",500,10,70,30,1,0,1000,0);

Subroutine("C:¥Program Files¥TECAN¥EVOware¥database¥Scripts¥Diluter_Aspirate_Air.esc",0);

PickUp_DITIs2(255,15,1,"0C08ｯ1000000000000",1,"Remove supernatant and add 100% EtOH",0,1,"DiTi 200 ul MIX1",0);

MoveLiha(255,15,1,1,"0C08ｯ1000000000000",4,3,25,400,1,"Remove supernatant and add 100% EtOH",0,1,0);

Subroutine("C:¥Program Files¥TECAN¥EVOware¥database¥Scripts¥Diluter_Dispense_Air.esc",0);

Aspirate(255,"Remove Supernatant","66","66","66","66","66","66","66","66",0,0,0,0,21,1,1,"0C08ｯ1000000000000",1,"Remove supernatant and add 100% EtOH",0,1,0);

Dispense(255,"Clearing contents of TIP","66","66","66","66","66","66","66","66",0,0,0,0,1,1,1,"0108ｯ1",0,0);

Set_DITIs_Back(255,15,1,"0C08ｯ1000000000000",1,"Remove supernatant and add 100% EtOH",0,1,0);

Wash(255,1,1,1,0,"2",500,"1",500,10,70,30,1,0,1000,0);

PickUp_DITIs2(255,2,1,"0C08ｯ1000000000000",0,"DiTi 200 ul EtOH USE",0);

Mix(255,"Solvent Mix","145","145","145","145","145","145","145","145",0,0,0,0,31,2,1,"0108ｯ1",2,0,0);

Aspirate(255,"Solvent for EtOH trough","140","140","140","140","140","140","140","140",0,0,0,0,31,2,1,"0108ｯ1",0,0);

Dispense(255,"Solvent","140","140","140","140","140","140","140","140",0,0,0,0,21,1,1,"0C08ｯ1000000000000",1,"Remove supernatant and add 100% EtOH",0,1,0);

Set_DITIs_Back(255,2,1,"0C08ｯ1000000000000",0,0);

EndLoop();

Vector("ET 2 LID <> SAFE","31","3",0,1,2,0,1,0);

GroupEnd();

Comment("Remove -1");

Comment("REMOVE Liquid from MAGNET plate ADD EtOH and REMOVE");

Vector("ET 1 LID <> SAFE","28","3",0,1,0,1,1,0);

Wash(255,1,1,1,0,"2",500,"1",500,10,70,30,1,0,1000,0);

BeginLoop("NC","Wash the AMPure beads and Add water for sample elution");

Comment(" Remove");

Subroutine("C:¥Program Files¥TECAN¥EVOware¥database¥Scripts¥Diluter_Aspirate_Air.esc",0);

PickUp_DITIs2(255,15,1,"0C08ｯ1000000000000",1,"Wash the AMPure beads and Add water for sample elution",0,1,"DiTi 200 ul MIX1",0);

MoveLiha(255,15,1,1,"0C08ｯ1000000000000",4,3,25,400,1,"Wash the AMPure beads and Add water for sample elution",0,1,0);

Subroutine("C:¥Program Files¥TECAN¥EVOware¥database¥Scripts¥Diluter_Dispense_Air.esc",0);

Aspirate(255,"Aspirate EtOH Low Volume Liquid Level","70","70","70","70","70","70","70","70",0,0,0,0,21,1,1,"0C08ｯ1000000000000",1,"Wash the AMPure beads and Add water for sample elution",0,1,0);

Dispense(255,"Clearing contents of TIP","70","70","70","70","70","70","70","70",0,0,0,0,1,1,1,"0108ｯ1",0,0);

Aspirate(255,"Remove Excess liquid from Magnet Plate","110","110","110","110","110","110","110","110",0,0,0,0,21,1,1,"0C08ｯ1000000000000",1,"Wash the AMPure beads and Add water for sample elution",0,1,0);

Dispense(255,"Clearing contents of TIP","110","110","110","110","110","110","110","110",0,0,0,0,1,1,1,"0108ｯ1",0,0);

Set_DITIs_Back(255,15,1,"0C08ｯ1000000000000",1,"Wash the AMPure beads and Add water for sample elution",0,1,0);

Wash(255,1,1,1,0,"2",500,"1",500,10,70,30,1,0,1000,0);

Comment("EtOH 200ul -1");

PickUp_DITIs2(255,2,1,"0C08ｯ1000000000000",0,"DiTi 200 ul EtOH USE",0);

Mix(255,"Solvent Mix","100","100","100","100","100","100","100","100",0,0,0,0,28,2,1,"0108ｯ1",2,0,0);

Aspirate(255,"Solvent for EtOH trough","100","100","100","100","100","100","100","100",0,0,0,0,28,2,1,"0108ｯ1",0,0);

Dispense(255,"Solvent","100","100","100","100","100","100","100","100",0,0,0,0,21,1,1,"0C08ｯ1000000000000",1,"Wash the AMPure beads and Add water for sample elution",0,1,0);

Aspirate(255,"Solvent for EtOH trough","100","100","100","100","100","100","100","100",0,0,0,0,28,2,1,"0108ｯ1",0,0);

Dispense(255,"Solvent","100","100","100","100","100","100","100","100",0,0,0,0,21,1,1,"0C08ｯ1000000000000",1,"Wash the AMPure beads and Add water for sample elution",0,1,0);

Detect_Liquid(255,"Solvent",28,2,1,"0108ｯ1",0,0);

Set_DITIs_Back(255,2,1,"0C08ｯ1000000000000",0,0);

Wash(255,1,1,1,0,"2",500,"1",500,10,70,30,1,0,1000,0);

Subroutine("C:¥Program Files¥TECAN¥EVOware¥database¥Scripts¥Diluter_Aspirate_Air.esc",0);

PickUp_DITIs2(255,15,1,"0C08ｯ1000000000000",1,"Wash the AMPure beads and Add water for sample elution",0,1,"DiTi 200 ul MIX1",0);

MoveLiha(255,15,1,1,"0C08ｯ1000000000000",4,3,25,400,1,"Wash the AMPure beads and Add water for sample elution",0,1,0);

Subroutine("C:¥Program Files¥TECAN¥EVOware¥database¥Scripts¥Diluter_Dispense_Air.esc",0);

Aspirate(255,"Aspirate EtOH Low Volume Liquid Level","120","120","120","120","120","120","120","120",0,0,0,0,21,1,1,"0C08ｯ1000000000000",1,"Wash the AMPure beads and Add water for sample elution",0,1,0);

Dispense(255,"Clearing contents of TIP","120","120","120","120","120","120","120","120",0,0,0,0,1,1,1,"0108ｯ1",0,0);

Aspirate(255,"Solvent without asp airgap at end","130","130","130","130","130","130","130","130",0,0,0,0,21,1,1,"0C08ｯ1000000000000",1,"Wash the AMPure beads and Add water for sample elution",0,1,0);

Dispense(255,"Clearing contents of TIP","130","130","130","130","130","130","130","130",0,0,0,0,1,1,1,"0108ｯ1",0,0);

Set_DITIs_Back(255,15,1,"0C08ｯ1000000000000",1,"Wash the AMPure beads and Add water for sample elution",0,1,0);

Wash(255,1,1,1,0,"2",500,"1",500,10,70,30,1,0,1000,0);

Comment("Pip EtOH 2");

PickUp_DITIs2(255,2,1,"0C08ｯ1000000000000",0,"DiTi 200 ul EtOH USE",0);

Aspirate(255,"Solvent for EtOH trough","100","100","100","100","100","100","100","100",0,0,0,0,28,2,1,"0108ｯ1",0,0);

Dispense(255,"Solvent","100","100","100","100","100","100","100","100",0,0,0,0,21,1,1,"0C08ｯ1000000000000",1,"Wash the AMPure beads and Add water for sample elution",0,1,0);

Aspirate(255,"Solvent for EtOH trough","100","100","100","100","100","100","100","100",0,0,0,0,28,2,1,"0108ｯ1",0,0);

Dispense(255,"Solvent","100","100","100","100","100","100","100","100",0,0,0,0,21,1,1,"0C08ｯ1000000000000",1,"Wash the AMPure beads and Add water for sample elution",0,1,0);

Detect_Liquid(255,"Solvent",28,2,1,"0108ｯ1",0,0);

Set_DITIs_Back(255,2,1,"0C08ｯ1000000000000",0,0);

Wash(255,1,1,1,0,"2",500,"1",500,10,70,30,1,0,1000,0);

Comment("Pip Top layer 2");

Subroutine("C:¥Program Files¥TECAN¥EVOware¥database¥Scripts¥Diluter_Aspirate_Air.esc",0);

PickUp_DITIs2(255,15,1,"0C08ｯ1000000000000",1,"Wash the AMPure beads and Add water for sample elution",0,1,"DiTi 200 ul MIX1",0);

MoveLiha(255,15,1,1,"0C08ｯ1000000000000",4,3,25,400,1,"Wash the AMPure beads and Add water for sample elution",0,1,0);

Subroutine("C:¥Program Files¥TECAN¥EVOware¥database¥Scripts¥Diluter_Dispense_Air.esc",0);

Aspirate(255,"Aspirate EtOH Low Volume Liquid Level","120","120","120","120","120","120","120","120",0,0,0,0,21,1,1,"0C08ｯ1000000000000",1,"Wash the AMPure beads and Add water for sample elution",0,1,0);

Dispense(255,"Clearing contents of TIP","120","120","120","120","120","120","120","120",0,0,0,0,1,1,1,"0108ｯ1",0,0);

Aspirate(255,"Solvent without asp airgap at end","70","70","70","70","70","70","70","70",0,0,0,0,21,1,1,"0C08ｯ1000000000000",1,"Wash the AMPure beads and Add water for sample elution",0,1,0);

Dispense(255,"Clearing contents of TIP","70","70","70","70","70","70","70","70",0,0,0,0,1,1,1,"0108ｯ1",0,0);

Aspirate(255,"Solvent","70","70","70","70","70","70","70","70",0,0,0,0,21,1,1,"0C08ｯ1000000000000",1,"Wash the AMPure beads and Add water for sample elution",0,1,0);

Dispense(255,"Clearing contents of TIP","70","70","70","70","70","70","70","70",0,0,0,0,1,1,1,"0108ｯ1",0,0);

Aspirate(255,"Clearing contents of TIP","100","100","100","100","100","100","100","100",0,0,0,0,1,1,1,"0108ｯ1",0,0);

Dispense(255,"Clearing contents of TIP variation","100","100","100","100","100","100","100","100",0,0,0,0,1,1,1,"0108ｯ1",0,0);

Set_DITIs_Back(255,15,1,"0C08ｯ1000000000000",1,"Wash the AMPure beads and Add water for sample elution",0,1,0);

Comment("Water 48ul");

Wash(255,1,1,1,0,"2",500,"1",500,10,70,30,1,0,1000,0);

PickUp_DITIs2(255,2,1,"0C0800ｬ70000000000",0,"DiTi 200 ul EtOH USE",0);

Aspirate(255,"SD Water free dispense","48","48","48","48","48","48","48","48",0,0,0,0,35,0,1,"0108ｯ1",0,0);

Dispense(255,"SD Water free dispense","48","48","48","48","48","48","48","48",0,0,0,0,21,1,1,"0C08ｯ1000000000000",1,"Wash the AMPure beads and Add water for sample elution",0,1,0);

Set_DITIs_Back(255,2,1,"0C0800ｬ70000000000",0,0);

EndLoop();

Vector("ET 1 LID <> SAFE","28","3",0,1,2,0,1,0);

Comment("Move from Magnet --> to SHIFT position");

Vector("SAFE <> Magnet","21","2",0,1,0,1,1,0);

Vector("MAGNET SHIFTING","21","2",0,0,2,2,1,0);

StartTimer("1");

WaitTimer("1","60");

Vector("MAGNET SHIFTING","21","2",1,0,2,2,1,0);

Vector("RT Assay 1 <> SAFE","21","1",0,1,2,0,1,0);

Vector("RT Assay 1 <> PUSHdown","21","1",0,1,0,0,1,0);

Vector("Roma FAR RIGHT","35","5",0,1,2,2,0,0);

Comment("Mix 48 SD on RT");

Comment("Move from Magnet --> RT Assay Area 1 ( wait 5 mins)");

Notification(1,"EVO Team","Confirmation:Plate stand: EVO 1st Information","Check Plate! About 30 min before",0);

BeginLoop("NC","Mix the sample for elution");

Wash(255,1,1,1,0,"2",500,"1",500,10,70,30,1,0,1000,0);

GetDITI2(255,"DiTi 200 ul",1,0,10,70);

Aspirate(255,"Mixing Beads with Sample small volume aspirate","5","5","5","5","5","5","5","5",0,0,0,0,21,0,1,"0C08ｯ1000000000000",1,"Mix the sample for elution",0,1,0);

IfThen("TEST_MODE",0,"0");

Variable(D,"5",0,"",0,0.000000,0.000000,0,2,0,0);

Else();

Variable(D,"30",0,"",0,0.000000,0.000000,0,2,0,0);

EndIf();

BeginLoop("D","Sample mixing");

Aspirate(255,"Mixing Beads with Sample small fast","25","25","25","25","25","25","25","25",0,0,0,0,21,0,1,"0C08ｯ1000000000000",1,"Mix the sample for elution",0,1,0);

Dispense(255,"Mixing Beads with Sample small fast","25","25","25","25","25","25","25","25",0,0,0,0,21,0,1,"0C08ｯ1000000000000",1,"Mix the sample for elution",0,1,0);

EndLoop();

Dispense(255,"Mixing Beads with Sample small volume aspirate","5","5","5","5","5","5","5","5",0,0,0,0,21,0,1,"0C08ｯ1000000000000",1,"Mix the sample for elution",0,1,0);

Detect_Liquid(255,"Mixing Beads with Sample small fast",21,0,1,"0C08ｯ1000000000000",1,"Mix the sample for elution",0,1,0);

Aspirate(255,"Clearing BEAD contents in TIP","20","20","20","20","20","20","20","20",0,0,0,0,21,0,1,"0C08ｯ1000000000000",1,"Mix the sample for elution",0,1,0);

Dispense(255,"Clearing BEAD contents in TIP_Z-max -9","20","20","20","20","20","20","20","20",0,0,0,0,21,0,1,"0C08ｯ1000000000000",1,"Mix the sample for elution",0,1,0);

Detect_Liquid(255,"Mixing Beads with Sample small fast",21,0,1,"0C08ｯ1000000000000",1,"Mix the sample for elution",0,1,0);

DropDITI(255,2,2,10,70,0);

EndLoop();

Wash(255,1,1,1,0,"2",500,"1",500,10,70,30,1,0,1000,0);

FACTS("ThermalCycler","ThermalCycler_OpenLid","1","0","");

Vector("RT Assay 1 <> SAFE","21","1",0,1,0,1,1,0);

Vector("SAFE <> Cycler","45","1",0,1,2,0,1,0);

FACTS("ThermalCycler","ThermalCycler_CloseLid","1,0","0","");

Comment("10.25 Stand(Position) was changed");

Notification(1,"EVO Team","Confirmation:Plate stand: EVO 1st Information","Check Plate!",0);

Vector("ExtractedLiquid <> SAFE","35","5",0,1,0,1,1,0);

Vector("RT Assay 1 <> SAFE","21","1",0,1,2,0,1,0);

Vector("RT Assay 1 <> PUSHdown","21","1",0,1,0,0,1,0);

Vector("RT Assay 1 <> SAFE","21","1",0,0,0,1,1,0);

Vector("RT Assay 1 <> SAFE","21","1",1,0,0,2,1,0);

ROMA(2,80,75,0,0,0,150,1,0);

Comment("10.25 Stand(Position) was changed");

IfThen("TEST_MODE",0,"0");

StartTimer("1");

WaitTimer("1","10");

Else();

StartTimer("1");

FACTS("ThermalCycler","ThermalCycler_RunBlock","1,SD42,BLOCK,ON,300","0","");

WaitTimer("1","315");

EndIf();

FACTS("ThermalCycler","ThermalCycler_OpenLid","1","0","");

Vector("SAFE <> Cycler","45","1",0,1,0,1,1,0);

Vector("SAFE <> Magnet","21","2",0,1,2,0,1,0);

Vector("SAFE <> PUSH down Magnet","21","2",0,1,0,2,1,0);

Comment("Move RT Assay Area 1 --> Magnet ( wait 5 mins )");

Vector("Roma FAR RIGHT","35","5",0,1,2,2,0,0);

StartTimer("1");

WaitTimer("1","10 * 60");

Comment("Remove -4");

BeginLoop("NC","Transfer the sample to New plate");

Wash(255,1,1,1,0,"2",500,"1",500,10,70,30,1,0,1000,0);

GetDITI2(255,"DiTi 200 ul",0,0,10,70);

Aspirate(255,"Left-over","53","53","53","53","53","53","53","53",0,0,0,0,21,1,1,"0C08ｯ1000000000000",1,"Transfer the sample to New plate",0,1,0);

Dispense(255,"AMpure Collection","53","53","53","53","53","53","53","53",0,0,0,0,21,0,1,"0C08ｯ1000000000000",1,"Transfer the sample to New plate",0,1,0);

Detect_Liquid(255,"Mixing Ampure inside Plate Large Volume MIX",21,0,1,"0C08ｯ1000000000000",1,"Transfer the sample to New plate",0,1,0);

DropDITI(255,2,2,10,70,0);

EndLoop();

Comment("Set LowDiti Eject position back to Original");

Comment("37 Incubate 10min.");

Comment("10.25 Stand(Position) was changed");

Vector("RT Assay 1 <> SAFE","21","1",0,1,0,1,0,0);

Vector("ExtractedLiquid <> SAFE","35","5",0,1,2,0,1,0);

Vector("ExtractedLiquid <> PUSHdown","35","5",0,1,0,0,1,0);

ROMA(2,80,75,0,0,0,150,1,0);

Comment("10.25 Stand(Position) was changed");

StartTimer("1");

WaitTimer("1","10 * 60");

GroupEnd();

Vector("ExtractedLiquid <> SAFE","35","5",0,1,0,1,1,0);

Vector("FourDegreesPlate <-> SAFE","8","4",0,1,2,0,1,0);

ROMA(2,80,75,0,0,0,150,1,0);

Notification(0,"EVO Team","Assay finish","Assay finish",0);

**---END---**

**7. OLIGREEN_NORMALIZATION_Ver5**

**---START---**

D3E53D0C

20110627_110644 Admin

Administrator

--{ RES }--

V;200

--{ CFG }--

999;219;32;

14;-1;30;248;-1;-1;-1;-1;-1;234;-1;-1;-1;-1;-1;-1;96;-1;-1;-1;-1;-1;34;-1;-1;-1;-1;-1;246;244;245;243;242;238;-1;-1;239;-1;-1;-1;-1;-1;-1;-1;-1;-1;-1;-1;-1;-1;-1;-1;-1;-1;-1;-1;-1;-1;-1;-1;-1;-1;-1;-1;-1;-1;-1;-1;-1;-1;-1;-1;-1;-1;-1;-1;-1;-1;-1;-1;-1;-1;-1;-1;-1;-1;-1;-1;-1;-1;-1;-1;-1;-1;-1;-1;-1;-1;-1;-1;

998;0;

998;3;Wash Station Cleaner shallow;Wash Station Waste;Wash Station Cleaner deep;

998;;;;

998;3;DiTi 50ul;DiTi 50ul;DiTi Waste;

998;DiTi 50ul-1;DiTi 50ul-2;Waste;

998;0;

998;0;

998;0;

998;0;

998;0;

998;4;DiTi 50ul G8;DiTi 50ul G8;DiTi 50ul G8;Axgen 96 PCR 2;

998;DiTi 50ul G8-1;DiTi 50ul G8-2;DiTi 50ul G8-3;Destination;

998;0;

998;0;

998;0;

998;0;

998;0;

998;0;

998;3;;;;

998;;;;

998;0;

998;0;

998;0;

998;0;

998;0;

998;3;96 PCR for Normalization;96 Well MAGNET PCR;384 Well Plate;

998;Sample;MAGNET;384 plate;

998;0;

998;0;

998;0;

998;0;

998;0;

998;3;;;;

998;;;;

998;3;;;;

998;;;;

998;3;;;;

998;;;;

998;3;;;;

998;;;;

998;3;Trough 100ml 96 position;Diluent Normalization;;

998;Oligreen;Diluent;;

998;6;;;;;;;

998;;;;;;;

998;0;

998;0;

998;7;;;;;;;;

998;;;;;;;;

998;0;

998;0;

998;0;

998;0;

998;0;

998;0;

998;0;

998;0;

998;0;

998;0;

998;0;

998;0;

998;0;

998;0;

998;0;

998;0;

998;0;

998;0;

998;0;

998;0;

998;0;

998;0;

998;0;

998;0;

998;0;

998;0;

998;0;

998;0;

998;0;

998;0;

998;0;

998;0;

998;0;

998;0;

998;0;

998;0;

998;0;

998;0;

998;0;

998;0;

998;0;

998;0;

998;0;

998;0;

998;0;

998;0;

998;0;

998;0;

998;0;

998;0;

998;0;

998;0;

998;0;

998;0;

998;0;

998;0;

998;0;

998;0;

998;0;

998;0;

998;0;

998;0;

998;0;

998;0;

998;3;

998;4;0;System;

998;0;1;Magellan;

998;0;0;Thermal Cycler;

998;2;

998;73;384 Well Plate;

998;236;96 Well PCR Plate;

998;1;

998;43;

998;45;

996;0;0;

--{ RPG }--

Notification(1,"EVO Team","Error: EVO 1st Information","Error",1);

Comment("Change script 20th Apr Kojima");

Comment("Change program of 'ConvertMagellanFile3.vbs' 13th May Kojima");

Variable(NUM_COLUMN,"12",1,"How many columns?",1,1.000000,12.000000,0,2,1,0);

Variable(TEST_MODE,"1",1,"TEST=0, Assay=1",0,0.000000,1.000000,0,2,1,0);

Command("C5SSP800,90,0",1,1,,,2,2,0);

Set_DITI_Counter2("DiTi 50ul","2","1","1",0);

Set_DITI_Counter2("DiTi 50ul G8","8","1","1",0);

UserPrompt("Please delete 「Result.asc」 file from 「Short cut to asc」 folder on desk top.",1,-1);

Wizard;Quantitation(5:OLIGREEN_FULL.mth,|1:Sample,Axgen 96 PCR,21,1,12,8,Reagent,255,96,0,1,21.Sample,^Sample^21^1^12^8^Axgen 96 PCR^MP 3Pos Fixed^27^,|22:,,,0,|23:384 plate,16,24,21,3,384 Well Plate,2,8,MP 3Pos Fixed,|SrcPos:1,qty,1,,|11:Oligreen,Trough 100ml 96 position,Reagent,1,Reagent,1,|Mix:8,5,Mixing 50ul DiTi,|W1:2,1,Wash Station Waste (1),Wash Station Cleaner shallow (1),1,0,0,1,1,|Wash:,,,,,,,1,|4:,,,,0,1,0,0,|41:,0,0,-1,1,|21:0,|3:,,,,0,,0,,|Diti:200,1,Waste,2,2,|)

Variable(NormalizationWorklistFile,"201011912586",0,"Prompt",0,1.000000,10.000000,1,2,0,0);

Variable(NormalizationRunName,"qty",0,"Prompt",0,1.000000,10.000000,1,2,0,0);

Variable(NormalizationQuantPlateName,"384_plate",0,"Prompt",0,1.000000,10.000000,1,2,0,0);

Variable(NormalizationMethod,"OLIGREEN_2.mth",0,"Prompt",0,1.000000,10.000000,1,2,0,0);

Variable(NormalizationVolumeFile,"C:¥Program_Files¥TECAN¥EVOware¥output¥201011912586.vol",0,"Prompt",0,1.000000,10.000000,1,2,0,0);

Group("Add Oligreen to 384 black plate");

Wash(255,1,1,1,0,"2",500,"1",500,10,70,30,1,0,1000,0);

GetDITI2(255,"DiTi 50ul G8",1,0,10,70);

BeginLoop("NUM_COLUMN","Add Oligreen to 384 black plate");

Aspirate(255,"Reagent","16","16","16","16","16","16","16","16",0,0,0,0,31,0,1,"0C08ｯ1000000000000",0,0);

Dispense(255,"Reagent 7","16","16","16","16","16","16","16","16",0,0,0,0,21,2,2,"1810・10000000000000000000000000000000000000000000000000000",1,"Add Oligreen to 384 black plate",0,1,0);

Detect_Liquid(255,"Detect liquid",21,2,2,"1810・10000000000000000000000000000000000000000000000000000",1,"Add Oligreen to 384 black plate",0,1,0);

EndLoop();

DropDITI(255,2,2,10,70,0);

GroupEnd();

Group("Add sample to 384 black plate");

BeginLoop("NUM_COLUMN","Add sample to 384 black plate");

Wash(255,1,1,1,0,"2",500,"1",500,10,70,30,1,0,1000,0);

GetDITI2(255,"DiTi 50ul G8",1,0,10,70);

Aspirate(255,"Reagent 4","4","4","4","4","4","4","4","4",0,0,0,0,21,0,1,"0C08ｯ1000000000000",1,"Add sample to 384 black plate",0,1,0);

Detect_Liquid(255,"Detect liquid",21,0,1,"0C08ｯ1000000000000",1,"Add sample to 384 black plate",0,1,0);

Dispense(255,"Reagent 7","4","4","4","4","4","4","4","4",0,0,0,0,21,2,2,"1810・10000000000000000000000000000000000000000000000000000",1,"Add sample to 384 black plate",0,1,0);

Mix(255,"Mixing 50ul DiTi","16","16","16","16","16","16","16","16",0,0,0,0,21,2,2,"1810・10000000000000000000000000000000000000000000000000000",7,1,"Add sample to 384 black plate",0,1,0);

Aspirate(255,"Reagent 5","9","9","9","9","9","9","9","9",0,0,0,0,21,2,2,"1810・10000000000000000000000000000000000000000000000000000",1,"Add sample to 384 black plate",0,1,0);

Dispense(255,"Reagent 5","9","9","9","9","9","9","9","9",0,0,0,0,21,2,2,"1810Z・0000000000000000000000000000000000000000000000000000",1,"Add sample to 384 black plate",0,1,0);

Detect_Liquid(255,"Detect liquid",21,2,2,"1810Z・0000000000000000000000000000000000000000000000000000",1,"Add sample to 384 black plate",0,1,0);

DropDITI(255,2,2,10,70,0);

EndLoop();

GroupEnd();

Group("Add Oligreen to Control sample ");

Wash(3,1,1,1,0,"2",500,"1",500,10,70,30,1,0,1000,0);

GetDITI2(3,"DiTi 50ul G8",0,0,10,70);

Aspirate(3,"Reagent 3","16","16",0,0,0,0,0,0,0,0,0,0,31,0,1,"0C0830000000000000",0,0);

Dispense(3,"Reagent 7","16","16",0,0,0,0,0,0,0,0,0,0,21,2,2,"181000000000000000000000000000000P1000000000000000000000000",0,0);

Mix(3,"Mixing 50ul DiTi","12","12",0,0,0,0,0,0,0,0,0,0,21,2,2,"181000000000000000000000000000000P1000000000000000000000000",5,0,0);

Aspirate(3,"Reagent 5","9","9",0,0,0,0,0,0,0,0,0,0,21,2,2,"181000000000000000000000000000000P1000000000000000000000000",0,0);

Dispense(3,"Reagent 5","9","9",0,0,0,0,0,0,0,0,0,0,21,2,2,"181000000000000000000000000000000p2000000000000000000000000",0,0);

Detect_Liquid(3,"Detect liquid",21,2,2,"181000000000000000000000000000000p2000000000000000000000000",0,0);

DropDITI(3,2,2,10,70,0);

GroupEnd();

Group("Add Oligreen to Standard sample ");

Wash(255,1,1,1,0,"2",500,"1",500,10,70,30,1,0,1000,0);

GetDITI2(31,"DiTi 50ul G8",0,0,10,70);

Aspirate(31,"Reagent","15","15","15","15","15",0,0,0,0,0,0,0,31,0,1,"0C08O0000000000000",0,0);

Dispense(31,"Reagent 7","15","15","15","15","15",0,0,0,0,0,0,0,21,2,3,"1810000000000000000000000000000xT20000000000000000000000000",0,0);

Mix(31,"Mixing 50ul DiTi","13","13","13","13","13",0,0,0,0,0,0,0,21,2,3,"1810000000000000000000000000000xT20000000000000000000000000",7,0,0);

Aspirate(31,"Reagent 6","9","9","9","9","9",0,0,0,0,0,0,0,21,2,3,"1810000000000000000000000000000xT20000000000000000000000000",0,0);

Dispense(31,"Reagent 6","9","9","9","9","9",0,0,0,0,0,0,0,21,2,3,"1810000000000000000000000000000@y40000000000000000000000000",0,0);

Detect_Liquid(31,"Detect liquid",21,2,3,"1810000000000000000000000000000@y40000000000000000000000000",0,0);

Comment("YSpacing Reset");

MoveLiha(31,21,2,2,"1810000000000000000000000000000XE00000000000000000000000000",3,0,0,20,0,0);

Comment("YSpacing Reset end");

Aspirate(31,"Reagent 6","9","9","9","9","9",0,0,0,0,0,0,0,21,2,3,"1810000000000000000000000000000xT20000000000000000000000000",0,0);

Dispense(31,"Reagent 6","9","9","9","9","9",0,0,0,0,0,0,0,21,2,3,"1810000000000000000000000000000PB90000000000000000000000000",0,0);

Detect_Liquid(31,"Detect liquid",21,2,3,"1810000000000000000000000000000PB90000000000000000000000000",0,0);

DropDITI(31,2,2,10,70,0);

GroupEnd();

FACTS("Magellan","Magellan_Open","","0","");

Comment("Place the plate moving commands here");

Transfer_Rack("21","43",1,0,0,0,0,"","384 Well Plate","Narrow","","","MP 3Pos Fixed","","Magellan","3","(Not defined)","1");

FACTS("Magellan","Magellan_Measure","C:¥Program Files¥TECAN¥EVOware¥output¥<YYYYMMDD-NNN>.wsp,OLIGREEN_2.mth,0,0","0","");

FACTS("Magellan","Magellan_Open","","0","");

Comment("Place the plate moving commands here");

Transfer_Rack("43","21",1,0,0,0,0,"","384 Well Plate","Narrow","","","Magellan","","MP 3Pos Fixed","1","(Not defined)","3");

ROMA(2,80,75,0,0,0,150,1,0);

FACTS("Magellan","Magellan_Close","","0","");

Comment("The parameters are: VolumeFilename QuantPlateFilename NormalizationPlateID");

Variable(UpdateVolumes,"",0,"Prompt",0,1.000000,10.000000,1,2,0,0);

Execute("C:¥Program Files¥TECAN¥EVOware¥Drivers¥Quantitation¥UpdateVolumes.exe ~NormalizationWorklistFile~ ~NormalizationQuantPlateName~ ~NormalizationRunName~",6,"UpdateVolumes",2);

EXECUTE_VBSCRIPT("C:¥Documents and Settings¥Administrator¥デスクトップ¥ConvertMagellanFile3.vbs",0);

WizardEnd();

UserPrompt("Please start speed vac for sample concentration.

Please set the plate when the treatment is finished.

If you click OK, the step will be restarted.",1,-1);

Group("Add water for DNA resuspension");

Comment("Add water for DNA resuspension");

BeginLoop("NUM_COLUMN","Add water for DNA resuspension");

Wash(255,1,1,1,0,"2",500,"1",500,10,70,30,1,0,1000,0);

GetDITI2(255,"DiTi 50ul G8",0,0,10,70);

Aspirate(255,"Reagent 8","10","10","10","10","10","10","10","10",0,0,0,0,31,1,1,"0108ｯ1",0,0);

Dispense(255,"Reagent 8","10","10","10","10","10","10","10","10",0,0,0,0,21,0,1,"0C08ｯ1000000000000",1,"Add water for DNA resuspension",0,1,0);

Detect_Liquid(255,"Detect liquid 2",21,0,1,"0C08ｯ1000000000000",1,"Add water for DNA resuspension",0,1,0);

DropDITI(255,2,2,10,70,0);

EndLoop();

UserPrompt("Please spin down sample plate.1000rpm 1sec",1,-1);

GroupEnd();

Group("37dc incubation");

FACTS("ThermalCycler","ThermalCycler_OpenLid","1","0","");

Vector("RT Assay 1 <> SAFE","21","1",0,1,0,1,1,0);

Vector("SAFE <> Cycler","45","1",0,1,2,0,1,0);

FACTS("ThermalCycler","ThermalCycler_CloseLid","1,0","0","");

IfThen("TEST_MODE",0,"1");

StartTimer("1");

FACTS("ThermalCycler","ThermalCycler_RunBlock1","HEAT37,BLOCK,ON,300","0","");

WaitTimer("1","315");

Else();

StartTimer("1");

WaitTimer("1","10");

EndIf();

FACTS("ThermalCycler","ThermalCycler_OpenLid","1","0","");

Vector("SAFE <> Cycler","45","1",0,1,0,1,1,0);

Vector("RT Assay 1 <> SAFE","21","1",0,1,2,0,1,0);

Vector("RT Assay 1 <> PUSHdown","21","1",0,1,0,0,0,0);

Vector("Roma FAR RIGHT","35","5",0,1,2,2,0,0);

GroupEnd();

Group("DNA resuspension");

Comment("Sample pipetting for DNA resuspension");

Comment("Change Mix position (0mm --> -1mm) 30th Mar Kojima");

BeginLoop("NUM_COLUMN","Sample pipetting for DNA resuspension");

Wash(255,1,1,1,0,"2",500,"1",500,10,70,30,1,0,1000,0);

GetDITI2(255,"DiTi 50ul",0,0,10,70);

Mix(255,"Mixing 50ul DiTi 2","8","8","8","8","8","8","8","8",0,0,0,0,21,0,1,"0C08ｯ1000000000000",10,1,"Sample pipetting for DNA resuspension",0,1,0);

Detect_Liquid(255,"Detect liquid",21,0,1,"0C08ｯ1000000000000",1,"Sample pipetting for DNA resuspension",0,1,0);

DropDITI(255,2,2,10,70,0);

EndLoop();

UserPrompt("Please spin down sample plate.1000rpm 1sec",1,-1);

UserPrompt("Please replace 「Result.asc」 file in 「Short cut to asc」 folder on desk top.

",1,-1);

GroupEnd();

Wizard;Normalization(PFBhcmFtZXRlclN0b3JlPjxHd2xOYW1lPkM6XFByb2dyYW0gRmlsZXNcVEVDQU5cRVZPd2FyZVxkYXRhYmFzZVx3b3JrbGlzdHNcTm9ybWFsaXphdGlvbldpemFyZFxOb3JtYWxpemF0aW9uXE5XXzIwMTAxMTA5MTMwNDU5MzkwNi5nd2w8L0d3bE5hbWU+PEpvYkxpc3RQbGFpbkZpbGVOYW1lPk5XXzIwMTAxMTA5MTMwNDU5MzkwNjwvSm9iTGlzdFBsYWluRmlsZU5hbWU+PFNPRVdpemFyZERhdGFGaWxlTmFtZT5DOlxQcm9ncmFtIEZpbGVzXFRFQ0FOXEVWT3dhcmVcRHJpdmVyc1xOb3JtYWxpemF0aW9uXFdpemFyZFxkYXRhXE5XX1NQREFUQV8yMDEwMTEwOTEzMDQ1OTM5MDYueG1sPC9TT0VXaXphcmREYXRhRmlsZU5hbWU+PFNhbXBsZUxhYndhcmVOYW1lIGxhYndhcmVUeXBlPSJBeGdlbiA5NiBQQ1IiIGNhcnJpZXJUeXBlPSJNUCAzUG9zIEZpeGVkIiBjYXJyaWVySUQ9IjI3IiBjYXJyaWVyTnVtYmVyT2ZTaXRlcz0iMyIgbGFid2FyZVhXZWxscz0iMTIiIGxhYndhcmVZV2VsbHM9IjgiPlNhbXBsZTwvU2FtcGxlTGFid2FyZU5hbWU+PFNvdXJjZUxpcXVpZGNsYXNzPlJlYWdlbnQ8L1NvdXJjZUxpcXVpZGNsYXNzPjxUYXJnZXRDb25jZW50cmF0aW9uPjE8L1RhcmdldENvbmNlbnRyYXRpb24+PE1pblNhbXBsZVRvVHJhbnNmZXI+MTwvTWluU2FtcGxlVG9UcmFuc2Zlcj48TWF4U2FtcGxlVG9UcmFuc2Zlcj4xMDwvTWF4U2FtcGxlVG9UcmFuc2Zlcj48UnVuc1RvZ2V0aGVyV2l0aFF1YW50aXRhdGlvbj5GYWxzZTwvUnVuc1RvZ2V0aGVyV2l0aFF1YW50aXRhdGlvbj48UHJvbXB0VXNlckZvckltcG9ydEZpbGU+VHJ1ZTwvUHJvbXB0VXNlckZvckltcG9ydEZpbGU+PEltcG9ydERpcmVjdG9yeT5DOlxQcm9ncmFtIEZpbGVzXFRFQ0FOXEVWT3dhcmVcb3V0cHV0XGFzY1w8L0ltcG9ydERpcmVjdG9yeT48UGxhaW5Db25jRmlsZW5hbWU+PC9QbGFpbkNvbmNGaWxlbmFtZT48SW1wb3J0VHlwZT5NYXRyaXg8L0ltcG9ydFR5cGU+PE1hdHJpeEZpbGVIYXNIZWFkZXJzPkZhbHNlPC9NYXRyaXhGaWxlSGFzSGVhZGVycz48VGlwVHlwZT5EaXRpPC9UaXBUeXBlPjxUaXBNYXNrPjExMTExMTExPC9UaXBNYXNrPjxEaWx1ZW50TGFid2FyZU5hbWUgbGFid2FyZVR5cGU9IkRpbHVlbnQgTm9ybWFsaXphdGlvbiIgY2FycmllclR5cGU9IkxJIC0gVHJvdWdoIDNQb3MgMTAwbWwgc2l0ZUIiIGNhcnJpZXJJRD0iOTMzIiBjYXJyaWVyTnVtYmVyT2ZTaXRlcz0iMyIgbGFid2FyZVhXZWxscz0iMSIgbGFid2FyZVlXZWxscz0iMSI+RGlsdWVudDwvRGlsdWVudExhYndhcmVOYW1lPjxEaWx1ZW50TGlxdWlkY2xhc3M+UmVhZ2VudDwvRGlsdWVudExpcXVpZGNsYXNzPjxEaXNwZW5zZVRvZ2V0aGVyPlRydWU8L0Rpc3BlbnNlVG9nZXRoZXI+PERpc3BlbnNlVG9nZXRoZXJMaXF1aWRjbGFzcz5SZWFnZW50PC9EaXNwZW5zZVRvZ2V0aGVyTGlxdWlkY2xhc3M+PE1peERpbHVlbnRBbmRTYW1wbGU+RmFsc2U8L01peERpbHVlbnRBbmRTYW1wbGU+PE1peFZvbHVtZT4wPC9NaXhWb2x1bWU+PE1peEN5Y2xlcz4wPC9NaXhDeWNsZXM+PE1peExpcXVpZGNsYXNzPjwvTWl4TGlxdWlkY2xhc3M+PElzSW5TaXR1PkZhbHNlPC9Jc0luU2l0dT48Tm9ybWFsaXphdGlvbkxhYndhcmVOYW1lIGxhYndhcmVUeXBlPSJBeGdlbiA5NiBQQ1IgMiIgY2FycmllclR5cGU9Ik1QIDRQb3MgQ29vbGluZyIgY2FycmllcklEPSIwIiBjYXJyaWVyTnVtYmVyT2ZTaXRlcz0iNCIgbGFid2FyZVhXZWxscz0iMTIiIGxhYndhcmVZV2VsbHM9IjgiPkRlc3RpbmF0aW9uPC9Ob3JtYWxpemF0aW9uTGFid2FyZU5hbWU+PFRhcmdldFdlbGxWb2x1bWU+MTA8L1RhcmdldFdlbGxWb2x1bWU+PFByaW50UmVwb3J0PlRydWU8L1ByaW50UmVwb3J0PjxTYXZlVG9Dc3Y+RmFsc2U8L1NhdmVUb0Nzdj48TWF4TnVtYmVyT2ZXZWxsc0luRXJyb3I+OTY8L01heE51bWJlck9mV2VsbHNJbkVycm9yPjxTa2lwU2FtcGxlc0lmQ29uY2VudHJhdGlvbklzVG9vTG93PjI8L1NraXBTYW1wbGVzSWZDb25jZW50cmF0aW9uSXNUb29Mb3c+PFNraXBTYW1wbGVzSWZDb25jZW50cmF0aW9uSXNUb29oSGlnaD5GYWxzZTwvU2tpcFNhbXBsZXNJZkNvbmNlbnRyYXRpb25Jc1Rvb2hIaWdoPjxXYXN0ZVZvbHVtZT4yPC9XYXN0ZVZvbHVtZT48Q2xlYW5lclZvbHVtZT4yPC9DbGVhbmVyVm9sdW1lPjxXYXN0ZUxhYndhcmVOYW1lIGxhYndhcmVUeXBlPSJ1bmtub3duIiBjYXJyaWVyVHlwZT0idW5rbm93biIgY2FycmllcklEPSJ1bmtub3duIiBjYXJyaWVyTnVtYmVyT2ZTaXRlcz0iMCIgbGFid2FyZVhXZWxscz0iMCIgbGFid2FyZVlXZWxscz0iMCIgZ3JpZD0iMSIgc2l0ZT0iMiI+V2FzaCBTdGF0aW9uIFdhc3RlICgxKTwvV2FzdGVMYWJ3YXJlTmFtZT48Q2xlYW5lckxhYndhcmVOYW1lIGxhYndhcmVUeXBlPSJ1bmtub3duIiBjYXJyaWVyVHlwZT0idW5rbm93biIgY2FycmllcklEPSJ1bmtub3duIiBjYXJyaWVyTnVtYmVyT2ZTaXRlcz0iMCIgbGFid2FyZVhXZWxscz0iMCIgbGFid2FyZVlXZWxscz0iMCIgZ3JpZD0iMSIgc2l0ZT0iMSI+V2FzaCBTdGF0aW9uIENsZWFuZXIgc2hhbGxvdyAoMSk8L0NsZWFuZXJMYWJ3YXJlTmFtZT48VXNlRmFzdFdhc2g+VHJ1ZTwvVXNlRmFzdFdhc2g+PFVzZVBpbmNoVmFsdmU+RmFsc2U8L1VzZVBpbmNoVmFsdmU+PFBlcmlvZGljV2FzaD4xPC9QZXJpb2RpY1dhc2g+PERlY29udGFtaW5hdGlvbldhc2hOZWVkZWQ+RmFsc2U8L0RlY29udGFtaW5hdGlvbldhc2hOZWVkZWQ+PERpdGlUeXBlc1RvVXNlPjUwPC9EaXRpVHlwZXNUb1VzZT48RGl0aVR5cGVzVG9Vc2VGb3JEaWx1ZW50PjUwPC9EaXRpVHlwZXNUb1VzZUZvckRpbHVlbnQ+PFJldHJ5RmV0Y2hpbmczVGltZXM+VHJ1ZTwvUmV0cnlGZXRjaGluZzNUaW1lcz48RGl0aVdhc3RlTGFid2FyZU5hbWUgbGFid2FyZVR5cGU9IiIgY2FycmllclR5cGU9IiIgY2FycmllcklEPSIiIGNhcnJpZXJOdW1iZXJPZlNpdGVzPSIwIiBsYWJ3YXJlWFdlbGxzPSIwIiBsYWJ3YXJlWVdlbGxzPSIwIj5XYXN0ZTwvRGl0aVdhc3RlTGFid2FyZU5hbWU+PERlY29udGFtaW5hdGlvbldhc2hWb2x1bWU+MDwvRGVjb250YW1pbmF0aW9uV2FzaFZvbHVtZT48RGVjb250YW1pbmF0aW9uV2FzaFNvdXJjZUxhYndhcmVOYW1lIGxhYndhcmVUeXBlPSIiIGNhcnJpZXJUeXBlPSIiIGNhcnJpZXJJRD0iIiBjYXJyaWVyTnVtYmVyT2ZTaXRlcz0iMCIgbGFid2FyZVhXZWxscz0iMCIgbGFid2FyZVlXZWxscz0iMCI+PC9EZWNvbnRhbWluYXRpb25XYXNoU291cmNlTGFid2FyZU5hbWU+PERlY29udGFtaW5hdGlvbldhc2hMaXF1aWRDbGFzcz48L0RlY29udGFtaW5hdGlvbldhc2hMaXF1aWRDbGFzcz48RGVjb250YW1pbmF0aW9uV2FzaERlbGF5QWZ0ZXJBc3BpcmF0ZT4xPC9EZWNvbnRhbWluYXRpb25XYXNoRGVsYXlBZnRlckFzcGlyYXRlPjxJc1ByZWRpbHV0aW9uPkZhbHNlPC9Jc1ByZWRpbHV0aW9uPjxQcmVkaWx1dGlvbldlbGxWb2x1bWU+PC9QcmVkaWx1dGlvbldlbGxWb2x1bWU+PFByZWRpbHV0aW9uV2VsbERlYWRWb2x1bWU+PC9QcmVkaWx1dGlvbldlbGxEZWFkVm9sdW1lPjxOdW1iZXJPZlNhbXBsZVRyYW5zZmVyc0JlZm9yZVdhc2g+MTwvTnVtYmVyT2ZTYW1wbGVUcmFuc2ZlcnNCZWZvcmVXYXNoPjxOdW1iZXJPZkRpbHVlbnRUcmFuc2ZlcnNCZWZvcmVXYXNoPjE8L051bWJlck9mRGlsdWVudFRyYW5zZmVyc0JlZm9yZVdhc2g+PE51bWJlck9mRGlzcGVuc2VzQmVmb3JlV2FzaD4xPC9OdW1iZXJPZkRpc3BlbnNlc0JlZm9yZVdhc2g+PERpbHVlbnRWb2x1bWVJc1Rvb1NtYWxsRXJyb3JIYW5kbGluZz4xPC9EaWx1ZW50Vm9sdW1lSXNUb29TbWFsbEVycm9ySGFuZGxpbmc+PExlYXZlU2tpcHBlZFdlbGxzRW1wdHk+RmFsc2U8L0xlYXZlU2tpcHBlZFdlbGxzRW1wdHk+PE1pblRhcmdldENvbmNlbnRyYXRpb25Ob3RTa2lwcGVkPjwvTWluVGFyZ2V0Q29uY2VudHJhdGlvbk5vdFNraXBwZWQ+PE1heFRhcmdldENvbmNlbnRyYXRpb25Ob3RTa2lwcGVkPjwvTWF4VGFyZ2V0Q29uY2VudHJhdGlvbk5vdFNraXBwZWQ+PC9QYXJhbWV0ZXJTdG9yZT4=)

Group("Normalization Variables");

Variable(NormalizationRunName,"qty",0,"",0,0.000000,0.000000,1,2,0,0);

Variable(NormalizationStartOffsetDestination,"0",0,"",0,0.000000,0.000000,0,2,0,0);

Variable(NormalizationStartOffsetSource,"0",0,"",0,0.000000,0.000000,0,2,0,0);

GroupEnd();

Group("Normalization Smart Pipette");

FACTS("Normalization","Normalization_GenSMPData","PFBhcmFtZXRlclN0b3JlPjxHd2xOYW1lPkM6XFByb2dyYW0gRmlsZXNcVEVDQU5cRVZPd2FyZVxkYXRhYmFzZVx3b3JrbGlzdHNcTm9ybWFsaXphdGlvbldpemFyZFxOb3JtYWxpemF0aW9uXE5XXzIwMTAxMTA5MTMwNDU5MzkwNi5nd2w8L0d3bE5hbWU+PEpvYkxpc3RQbGFpbkZpbGVOYW1lPk5XXzIwMTAxMTA5MTMwNDU5MzkwNjwvSm9iTGlzdFBsYWluRmlsZU5hbWU+PFNPRVdpemFyZERhdGFGaWxlTmFtZT5DOlxQcm9ncmFtIEZpbGVzXFRFQ0FOXEVWT3dhcmVcRHJpdmVyc1xOb3JtYWxpemF0aW9uXFdpemFyZFxkYXRhXE5XX1NQREFUQV8yMDEwMTEwOTEzMDQ1OTM5MDYueG1sPC9TT0VXaXphcmREYXRhRmlsZU5hbWU+PFNhbXBsZUxhYndhcmVOYW1lIGxhYndhcmVUeXBlPSJBeGdlbiA5NiBQQ1IiIGNhcnJpZXJUeXBlPSJNUCAzUG9zIEZpeGVkIiBjYXJyaWVySUQ9IjI3IiBjYXJyaWVyTnVtYmVyT2ZTaXRlcz0iMyIgbGFid2FyZVhXZWxscz0iMTIiIGxhYndhcmVZV2VsbHM9IjgiPlNhbXBsZTwvU2FtcGxlTGFid2FyZU5hbWU+PFNvdXJjZUxpcXVpZGNsYXNzPlJlYWdlbnQ8L1NvdXJjZUxpcXVpZGNsYXNzPjxUYXJnZXRDb25jZW50cmF0aW9uPjE8L1RhcmdldENvbmNlbnRyYXRpb24+PE1pblNhbXBsZVRvVHJhbnNmZXI+MTwvTWluU2FtcGxlVG9UcmFuc2Zlcj48TWF4U2FtcGxlVG9UcmFuc2Zlcj4xMDwvTWF4U2FtcGxlVG9UcmFuc2Zlcj48UnVuc1RvZ2V0aGVyV2l0aFF1YW50aXRhdGlvbj5GYWxzZTwvUnVuc1RvZ2V0aGVyV2l0aFF1YW50aXRhdGlvbj48UHJvbXB0VXNlckZvckltcG9ydEZpbGU+VHJ1ZTwvUHJvbXB0VXNlckZvckltcG9ydEZpbGU+PEltcG9ydERpcmVjdG9yeT5DOlxQcm9ncmFtIEZpbGVzXFRFQ0FOXEVWT3dhcmVcb3V0cHV0XGFzY1w8L0ltcG9ydERpcmVjdG9yeT48UGxhaW5Db25jRmlsZW5hbWU+PC9QbGFpbkNvbmNGaWxlbmFtZT48SW1wb3J0VHlwZT5NYXRyaXg8L0ltcG9ydFR5cGU+PE1hdHJpeEZpbGVIYXNIZWFkZXJzPkZhbHNlPC9NYXRyaXhGaWxlSGFzSGVhZGVycz48VGlwVHlwZT5EaXRpPC9UaXBUeXBlPjxUaXBNYXNrPjExMTExMTExPC9UaXBNYXNrPjxEaWx1ZW50TGFid2FyZU5hbWUgbGFid2FyZVR5cGU9IkRpbHVlbnQgTm9ybWFsaXphdGlvbiIgY2FycmllclR5cGU9IkxJIC0gVHJvdWdoIDNQb3MgMTAwbWwgc2l0ZUIiIGNhcnJpZXJJRD0iOTMzIiBjYXJyaWVyTnVtYmVyT2ZTaXRlcz0iMyIgbGFid2FyZVhXZWxscz0iMSIgbGFid2FyZVlXZWxscz0iMSI+RGlsdWVudDwvRGlsdWVudExhYndhcmVOYW1lPjxEaWx1ZW50TGlxdWlkY2xhc3M+UmVhZ2VudDwvRGlsdWVudExpcXVpZGNsYXNzPjxEaXNwZW5zZVRvZ2V0aGVyPlRydWU8L0Rpc3BlbnNlVG9nZXRoZXI+PERpc3BlbnNlVG9nZXRoZXJMaXF1aWRjbGFzcz5SZWFnZW50PC9EaXNwZW5zZVRvZ2V0aGVyTGlxdWlkY2xhc3M+PE1peERpbHVlbnRBbmRTYW1wbGU+RmFsc2U8L01peERpbHVlbnRBbmRTYW1wbGU+PE1peFZvbHVtZT4wPC9NaXhWb2x1bWU+PE1peEN5Y2xlcz4wPC9NaXhDeWNsZXM+PE1peExpcXVpZGNsYXNzPjwvTWl4TGlxdWlkY2xhc3M+PElzSW5TaXR1PkZhbHNlPC9Jc0luU2l0dT48Tm9ybWFsaXphdGlvbkxhYndhcmVOYW1lIGxhYndhcmVUeXBlPSJBeGdlbiA5NiBQQ1IgMiIgY2FycmllclR5cGU9Ik1QIDRQb3MgQ29vbGluZyIgY2FycmllcklEPSIwIiBjYXJyaWVyTnVtYmVyT2ZTaXRlcz0iNCIgbGFid2FyZVhXZWxscz0iMTIiIGxhYndhcmVZV2VsbHM9IjgiPkRlc3RpbmF0aW9uPC9Ob3JtYWxpemF0aW9uTGFid2FyZU5hbWU+PFRhcmdldFdlbGxWb2x1bWU+MTA8L1RhcmdldFdlbGxWb2x1bWU+PFByaW50UmVwb3J0PlRydWU8L1ByaW50UmVwb3J0PjxTYXZlVG9Dc3Y+RmFsc2U8L1NhdmVUb0Nzdj48TWF4TnVtYmVyT2ZXZWxsc0luRXJyb3I+OTY8L01heE51bWJlck9mV2VsbHNJbkVycm9yPjxTa2lwU2FtcGxlc0lmQ29uY2VudHJhdGlvbklzVG9vTG93PjI8L1NraXBTYW1wbGVzSWZDb25jZW50cmF0aW9uSXNUb29Mb3c+PFNraXBTYW1wbGVzSWZDb25jZW50cmF0aW9uSXNUb29oSGlnaD5GYWxzZTwvU2tpcFNhbXBsZXNJZkNvbmNlbnRyYXRpb25Jc1Rvb2hIaWdoPjxXYXN0ZVZvbHVtZT4yPC9XYXN0ZVZvbHVtZT48Q2xlYW5lclZvbHVtZT4yPC9DbGVhbmVyVm9sdW1lPjxXYXN0ZUxhYndhcmVOYW1lIGxhYndhcmVUeXBlPSJ1bmtub3duIiBjYXJyaWVyVHlwZT0idW5rbm93biIgY2FycmllcklEPSJ1bmtub3duIiBjYXJyaWVyTnVtYmVyT2ZTaXRlcz0iMCIgbGFid2FyZVhXZWxscz0iMCIgbGFid2FyZVlXZWxscz0iMCIgZ3JpZD0iMSIgc2l0ZT0iMiI+V2FzaCBTdGF0aW9uIFdhc3RlICgxKTwvV2FzdGVMYWJ3YXJlTmFtZT48Q2xlYW5lckxhYndhcmVOYW1lIGxhYndhcmVUeXBlPSJ1bmtub3duIiBjYXJyaWVyVHlwZT0idW5rbm93biIgY2FycmllcklEPSJ1bmtub3duIiBjYXJyaWVyTnVtYmVyT2ZTaXRlcz0iMCIgbGFid2FyZVhXZWxscz0iMCIgbGFid2FyZVlXZWxscz0iMCIgZ3JpZD0iMSIgc2l0ZT0iMSI+V2FzaCBTdGF0aW9uIENsZWFuZXIgc2hhbGxvdyAoMSk8L0NsZWFuZXJMYWJ3YXJlTmFtZT48VXNlRmFzdFdhc2g+VHJ1ZTwvVXNlRmFzdFdhc2g+PFVzZVBpbmNoVmFsdmU+RmFsc2U8L1VzZVBpbmNoVmFsdmU+PFBlcmlvZGljV2FzaD4xPC9QZXJpb2RpY1dhc2g+PERlY29udGFtaW5hdGlvbldhc2hOZWVkZWQ+RmFsc2U8L0RlY29udGFtaW5hdGlvbldhc2hOZWVkZWQ+PERpdGlUeXBlc1RvVXNlPjUwPC9EaXRpVHlwZXNUb1VzZT48RGl0aVR5cGVzVG9Vc2VGb3JEaWx1ZW50PjUwPC9EaXRpVHlwZXNUb1VzZUZvckRpbHVlbnQ+PFJldHJ5RmV0Y2hpbmczVGltZXM+VHJ1ZTwvUmV0cnlGZXRjaGluZzNUaW1lcz48RGl0aVdhc3RlTGFid2FyZU5hbWUgbGFid2FyZVR5cGU9IiIgY2FycmllclR5cGU9IiIgY2FycmllcklEPSIiIGNhcnJpZXJOdW1iZXJPZlNpdGVzPSIwIiBsYWJ3YXJlWFdlbGxzPSIwIiBsYWJ3YXJlWVdlbGxzPSIwIj5XYXN0ZTwvRGl0aVdhc3RlTGFid2FyZU5hbWU+PERlY29udGFtaW5hdGlvbldhc2hWb2x1bWU+MDwvRGVjb250YW1pbmF0aW9uV2FzaFZvbHVtZT48RGVjb250YW1pbmF0aW9uV2FzaFNvdXJjZUxhYndhcmVOYW1lIGxhYndhcmVUeXBlPSIiIGNhcnJpZXJUeXBlPSIiIGNhcnJpZXJJRD0iIiBjYXJyaWVyTnVtYmVyT2ZTaXRlcz0iMCIgbGFid2FyZVhXZWxscz0iMCIgbGFid2FyZVlXZWxscz0iMCI+PC9EZWNvbnRhbWluYXRpb25XYXNoU291cmNlTGFid2FyZU5hbWU+PERlY29udGFtaW5hdGlvbldhc2hMaXF1aWRDbGFzcz48L0RlY29udGFtaW5hdGlvbldhc2hMaXF1aWRDbGFzcz48RGVjb250YW1pbmF0aW9uV2FzaERlbGF5QWZ0ZXJBc3BpcmF0ZT4xPC9EZWNvbnRhbWluYXRpb25XYXNoRGVsYXlBZnRlckFzcGlyYXRlPjxJc1ByZWRpbHV0aW9uPkZhbHNlPC9Jc1ByZWRpbHV0aW9uPjxQcmVkaWx1dGlvbldlbGxWb2x1bWU+PC9QcmVkaWx1dGlvbldlbGxWb2x1bWU+PFByZWRpbHV0aW9uV2VsbERlYWRWb2x1bWU+PC9QcmVkaWx1dGlvbldlbGxEZWFkVm9sdW1lPjxOdW1iZXJPZlNhbXBsZVRyYW5zZmVyc0JlZm9yZVdhc2g+MTwvTnVtYmVyT2ZTYW1wbGVUcmFuc2ZlcnNCZWZvcmVXYXNoPjxOdW1iZXJPZkRpbHVlbnRUcmFuc2ZlcnNCZWZvcmVXYXNoPjE8L051bWJlck9mRGlsdWVudFRyYW5zZmVyc0JlZm9yZVdhc2g+PE51bWJlck9mRGlzcGVuc2VzQmVmb3JlV2FzaD4xPC9OdW1iZXJPZkRpc3BlbnNlc0JlZm9yZVdhc2g+PERpbHVlbnRWb2x1bWVJc1Rvb1NtYWxsRXJyb3JIYW5kbGluZz4xPC9EaWx1ZW50Vm9sdW1lSXNUb29TbWFsbEVycm9ySGFuZGxpbmc+PExlYXZlU2tpcHBlZFdlbGxzRW1wdHk+RmFsc2U8L0xlYXZlU2tpcHBlZFdlbGxzRW1wdHk+PE1pblRhcmdldENvbmNlbnRyYXRpb25Ob3RTa2lwcGVkPjwvTWluVGFyZ2V0Q29uY2VudHJhdGlvbk5vdFNraXBwZWQ+PE1heFRhcmdldENvbmNlbnRyYXRpb25Ob3RTa2lwcGVkPjwvTWF4VGFyZ2V0Q29uY2VudHJhdGlvbk5vdFNraXBwZWQ+PC9QYXJhbWV0ZXJTdG9yZT4=","0","");

FACTS("NormSmartPipette","NormSmartPipette_ReadWizardData","NW_SPDATA_201011091304593906.xml*C:¥Program Files¥TECAN¥EVOware¥Drivers¥Normalization¥Wizard¥data¥NW_SPDATA_201011091304593906.xml","0","");

FACTS("NormSmartPipette","NormSmartPipette_GenerateGWL","C:¥Program Files¥TECAN¥EVOware¥database¥worklists¥NormalizationWizard¥Normalization¥NW_201011091304593906.gwl","0","");

Periodic_Wash(255,1,1,1,0,"2",500,"2",500,10,70,30,1,0,"1",1000,0);

Worklist(0,C:¥Program Files¥TECAN¥EVOware¥database¥worklists¥NormalizationWizard¥Normalization¥NW_201011091304593906_PRE.gwl,255,"Water",0,0);Wash(255,1,1,1,0,"2",500,"2",500,10,70,30,1,0,1000,0);GetDITI(255,0,1,0,0,0);DropDITI(255,2,2,10,70,0);

Worklist(1,,255,"Water",0,0);GetDITI(255,0,1,0,0,0);DropDITI(255,2,2,10,70,0);

Worklist(0,C:¥Program Files¥TECAN¥EVOware¥database¥worklists¥NormalizationWizard¥Normalization¥NW_201011091304593906_POST.gwl,255,"Water",0,0);Wash(255,1,1,1,0,"2",500,"2",500,10,70,30,1,0,1000,0);GetDITI(255,0,1,0,0,0);DropDITI(255,2,2,10,70,0);

Worklist(1,,255,"Water",0,0);GetDITI(255,0,1,0,0,0);DropDITI(255,2,2,10,70,0);

Worklist(0,C:¥Program Files¥TECAN¥EVOware¥database¥worklists¥NormalizationWizard¥Normalization¥NW_201011091304593906.gwl,255,"Water",0,0);Wash(255,1,1,1,0,"2",500,"2",500,10,70,30,1,0,1000,0);GetDITI(255,0,1,0,0,0);DropDITI(255,2,2,10,70,0);

Worklist(1,,255,"Water",0,0);GetDITI(255,0,1,0,0,0);DropDITI(255,2,2,10,70,0);

GroupEnd();

Wash(255,1,1,1,0,"2",500,"2",500,10,70,30,1,0,1000,0);

FACTS("Normalization","Normalization_GenCalcReport","","0","");

Comment("Print the Report/Save CSV file (parameters: -f NormalizationPlateID -p -s)");

Execute("C:¥Program Files¥TECAN¥EVOware¥Drivers¥Normalization¥Driver¥NormalizationReport.exe -f ~NormalizationRunName~ -p",2,"",2);

WizardEnd();

Notification(0,"EVO Team","Assay finish","Assay finish",0);

**---END---**

**8. POLY_A_TAILING**

**---START---**

-663E8EFC

20110627_161430 Admin

Administrator

--{ RES }--

V;200

--{ CFG }--

999;219;32;

14;-1;30;8;-1;-1;-1;-1;-1;234;-1;-1;-1;-1;-1;-1;96;-1;-1;-1;-1;-1;34;-1;-1;-1;-1;-1;246;244;245;243;242;238;-1;-1;239;-1;-1;-1;-1;-1;-1;-1;-1;-1;-1;-1;-1;-1;-1;-1;-1;-1;-1;-1;-1;-1;-1;-1;-1;-1;-1;-1;-1;-1;-1;-1;-1;-1;-1;-1;-1;-1;-1;-1;-1;-1;-1;-1;-1;-1;-1;-1;-1;-1;-1;-1;-1;-1;-1;-1;-1;-1;-1;-1;-1;-1;-1;-1;

998;0;

998;3;Wash Station Cleaner shallow;Wash Station Waste;Wash Station Cleaner deep;

998;;;;

998;3;;;DiTi Waste;

998;;;Waste;

998;0;

998;0;

998;0;

998;0;

998;0;

998;4;DiTi 50ul G8;DiTi 50ul G8;DiTi 50ul G8;Axgen 96 PCR 2;

998;DiTi 50ul G8-1;DiTi 50ul G8-2;DiTi 50ul G8-3;Sample;

998;0;

998;0;

998;0;

998;0;

998;0;

998;0;

998;3;;;;

998;;;;

998;0;

998;0;

998;0;

998;0;

998;0;

998;3;;96 Well MAGNET PCR;;

998;;MAGNET;;

998;0;

998;0;

998;0;

998;0;

998;0;

998;3;;;;

998;;;;

998;3;;;;

998;;;;

998;3;;;;

998;;;;

998;3;;;;

998;;;;

998;3;;;;

998;;;;

998;6;0.5 ml Eppendorf Tube Ox;0.5 ml Eppendorf Tube Enz2;0.5 ml Eppendorf Tube Enz2;;;;

998;TB;TR;BL;;;;

998;0;

998;0;

998;7;;;;;;;;

998;;;;;;;;

998;0;

998;0;

998;0;

998;0;

998;0;

998;0;

998;0;

998;0;

998;0;

998;0;

998;0;

998;0;

998;0;

998;0;

998;0;

998;0;

998;0;

998;0;

998;0;

998;0;

998;0;

998;0;

998;0;

998;0;

998;0;

998;0;

998;0;

998;0;

998;0;

998;0;

998;0;

998;0;

998;0;

998;0;

998;0;

998;0;

998;0;

998;0;

998;0;

998;0;

998;0;

998;0;

998;0;

998;0;

998;0;

998;0;

998;0;

998;0;

998;0;

998;0;

998;0;

998;0;

998;0;

998;0;

998;0;

998;0;

998;0;

998;0;

998;0;

998;0;

998;0;

998;0;

998;0;

998;2;

998;86;41;

998;241;10;

998;5;

998;4;0;System;

998;0;3;Magellan;

998;0;0;Thermal Cycler;

998;0;1;Hotel 4Pos DeepWell;

998;0;2;Hotel 2Pos DitiRack 1;

998;3;

998;236;96 Well PCR Plate;

998;86;96 Well PCR Plate;

998;241;DiTi 200 ul;

998;1;

998;43;

998;45;

998;41;

998;10;

996;0;0;

--{ RPG }--

Notification(1,"EVO Team","Error: EVO 1st Information","Error",1);

Comment("Change script 20th Apr Kojima");

Set_DITI_Counter2("DiTi 50ul G8","8","1","1",0);

Command("C5SSP800,90,0",1,1,,,2,2,0);

Variable(NUM_COLUMNS,"12",1,"How many lane ?",1,1.000000,12.000000,0,2,1,0);

Variable(TEST_MODE,"1",1,"TEST=0, Assay=1",0,0.000000,1.000000,0,2,1,0);

If("1",0,"1","HERE");

Comment("HERE");

FACTS("ThermalCycler","ThermalCycler_CloseLid","1,0","0","");

Subroutine("C:¥Program Files¥TECAN¥EVOware¥database¥Scripts¥PRE_HEATING.esc",1);

Group("Buffer");

Wash(255,1,1,1,0,"2",500,"1",500,10,70,30,1,0,1000,0);

BeginLoop("NUM_COLUMNS","Add Blocking mixture to sample ");

GetDITI2(255,"DiTi 50ul G8",0,0,10,70);

Aspirate(255,"Reagent 2","4","4","4","4","4","4","4","4",0,0,0,0,32,0,1,"0108ｯ1",0,0);

Detect_Liquid(255,"Reagent",32,0,1,"0108ｯ1",0,0);

Dispense(255,"Reagent 3","4","4","4","4","4","4","4","4",0,0,0,0,8,3,1,"0C08ｯ1000000000000",1,"Add Blocking mixture to sample ",0,1,0);

Mix(255,"Mixing 50ul DiTi","10","10","10","10","10","10","10","10",0,0,0,0,8,3,1,"0C08ｯ1000000000000",5,1,"Add Blocking mixture to sample ",0,1,0);

Detect_Liquid(255,"Reagent",8,3,1,"0C08ｯ1000000000000",1,"Add Blocking mixture to sample ",0,1,0);

DropDITI(255,2,2,10,70,0);

EndLoop();

UserPrompt("Please seal to plate",1,-1);

WaitTimer("1","75");

FACTS("ThermalCycler","ThermalCycler_OpenLid","1","0","");

Vector("FourDegreesPlate <-> SAFE","8","4",0,1,0,1,1,0);

Vector("AX SAFE <> Cycler","45","1",0,1,2,0,1,0);

Vector("AX SAFE <> Cycler","45","1",0,0,0,1,1,0);

Vector("AX SAFE <> Cycler","45","1",1,0,0,2,1,0);

FACTS("ThermalCycler","ThermalCycler_CloseLid","1,0","0","");

Comment("HEAT SHOCK 5min @ 95C");

IfThen("TEST_MODE",0,"0");

StartTimer("1");

WaitTimer("1","10");

Else();

StartTimer("1");

FACTS("ThermalCycler","ThermalCycler_RunBlock","1,HEAT95,BLOCK,ON,300","0","");

WaitTimer("1","405");

EndIf();

FACTS("ThermalCycler","ThermalCycler_OpenLid","1","0","");

Vector("AX SAFE <> Cycler","45","1",0,1,0,1,1,0);

Vector("AX FourDegreesPlate <-> SAFE","8","4",0,1,2,0,1,0);

Vector("AX FourDegreesPlate <->PUSHdown","8","4",0,1,0,2,0,0);

ROMA(2,80,75,0,0,0,150,1,0);

GroupEnd();

StartTimer("1");

WaitTimer("1","120");

UserPrompt("Please remove the seal from plate",1,-1);

Group("PolyA tailing Reaction Mix");

Wash(255,1,1,1,0,"2",500,"1",500,10,70,30,1,0,1000,0);

BeginLoop("NUM_COLUMNS","Add PolyA tailing mixture to sample");

GetDITI2(255,"DiTi 50ul G8",0,0,10,70);

Aspirate(255,"Reagent 2","6.0","6.0","6.0","6.0","6.0","6.0","6.0","6.0",0,0,0,0,32,1,1,"0108ｯ1",0,0);

Detect_Liquid(255,"Reagent",32,1,1,"0108ｯ1",0,0);

Dispense(255,"Reagent 3","6.0","6.0","6.0","6.0","6.0","6.0","6.0","6.0",0,0,0,0,8,3,1,"0C08ｯ1000000000000",1,"Add PolyA tailing mixture to sample",0,1,0);

Mix(255,"Mixing 50ul DiTi","18","18","18","18","18","18","18","18",0,0,0,0,8,3,1,"0C08ｯ1000000000000",5,1,"Add PolyA tailing mixture to sample",0,1,0);

Detect_Liquid(255,"Reagent",8,3,1,"0C08ｯ1000000000000",1,"Add PolyA tailing mixture to sample",0,1,0);

DropDITI(255,2,2,10,70,0);

EndLoop();

UserPrompt("Please seal to plate",1,-1);

FACTS("ThermalCycler","ThermalCycler_OpenLid","1","0","");

Vector("AX FourDegreesPlate <-> SAFE","8","4",0,1,0,1,1,0);

Vector("AX SAFE <> Cycler","45","1",0,1,2,0,1,0);

Vector("AX SAFE <> Cycler","45","1",0,0,0,1,1,0);

Vector("AX SAFE <> Cycler","45","1",1,0,0,2,1,0);

FACTS("ThermalCycler","ThermalCycler_CloseLid","1,0","0","");

IfThen("TEST_MODE",0,"0");

StartTimer("1");

WaitTimer("1","10");

Else();

Comment("Tailing reaction");

StartTimer("1");

FACTS("ThermalCycler","ThermalCycler_RunBlock","1,TAILING,BLOCK,ON,3900","0","");

WaitTimer("1","4000");

EndIf();

GroupEnd();

FACTS("ThermalCycler","ThermalCycler_OpenLid","1","0","");

Vector("SAFE <> Cycler","45","1",0,1,0,1,1,0);

Vector("FourDegreesPlate <-> SAFE","8","4",0,1,2,0,1,0);

Vector("FourDegreesPlate <->PUSHdown","8","4",0,1,0,2,1,0);

ROMA(2,80,75,0,0,0,150,1,0);

StartTimer("1");

WaitTimer("1","120");

UserPrompt("Please remove the seal from plate",1,-1);

Group("Blocking");

Wash(255,1,1,1,0,"2",500,"1",500,10,70,30,1,0,1000,0);

BeginLoop("NUM_COLUMNS","Add to Blocking mixture to sample ");

GetDITI2(255,"DiTi 50ul G8",0,0,10,70);

Aspirate(255,"Reagent 2","10","10","10","10","10","10","10","10",0,0,0,0,32,2,1,"0108ｯ1",0,0);

Detect_Liquid(255,"Reagent",32,2,1,"0108ｯ1",0,0);

Dispense(255,"Reagent 3","10","10","10","10","10","10","10","10",0,0,0,0,8,3,1,"0C08ｯ1000000000000",1,"Add to Blocking mixture to sample ",0,1,0);

Mix(255,"Mixing 50ul DiTi","25","25","25","25","25","25","25","25",0,0,0,0,8,3,1,"0C08ｯ1000000000000",5,1,"Add to Blocking mixture to sample ",0,1,0);

Detect_Liquid(255,"Reagent",8,3,1,"0C08ｯ1000000000000",1,"Add to Blocking mixture to sample ",0,1,0);

DropDITI(255,2,2,10,70,0);

EndLoop();

FACTS("ThermalCycler","ThermalCycler_OpenLid","1","0","");

UserPrompt("Please seal to plate ",1,-1);

Vector("AX FourDegreesPlate <-> SAFE","8","4",0,1,0,1,1,0);

Vector("AX SAFE <> Cycler","45","1",0,1,2,0,1,0);

Vector("AX SAFE <> Cycler","45","1",0,0,0,1,1,0);

Vector("AX SAFE <> Cycler","45","1",1,0,0,2,1,0);

FACTS("ThermalCycler","ThermalCycler_CloseLid","1,0","0","");

IfThen("TEST_MODE",0,"0");

StartTimer("1");

WaitTimer("1","10");

Else();

Comment("Blocking reaction");

StartTimer("1");

FACTS("ThermalCycler","ThermalCycler_RunBlock","1,BLOCKING,BLOCK,ON,4800","0","");

WaitTimer("1","4830");

EndIf();

FACTS("ThermalCycler","ThermalCycler_OpenLid","1","0","");

Vector("AX SAFE <> Cycler","45","1",0,1,0,1,1,0);

Vector("AX FourDegreesPlate <-> SAFE","8","4",0,1,2,0,1,0);

Vector("AX FourDegreesPlate <->PUSHdown","8","4",0,1,0,2,0,0);

ROMA(2,80,75,0,0,0,150,1,0);

GroupEnd();

Notification(0,"EVO Team","Assay finish","Assay finish",0);

**--END---**
