# Supplementary material for: Automated Workflow for Preparation of cDNA for Cap Analysis of Gene Expression on a Single Molecule Sequencer
Source: PLoS One. 2012 Jan 30;7(1):e30809. doi: 10.1371/journal.pone.0030809 (PMC3268765; doi:10.1371/journal.pone.0030809)
Supplement: Text S2 — Simplified CAGE cDNA preparation protocol for HeliScope sequencing by manual. The protocol for the manual preparation of HeliScope CAGE cDNA using 8-channel multi-pipette is described. Double exclamation mark starting sentences in red are attention in this protocol. Sharp mark sentences in green are the hints. Asterisk mark sentences in purple are safe rest points to suspend the protocol. (DOC) [file pone.0030809.s008.doc]

High Throughput HeliScope CAGE cDNA Preparation Protocol for Manual Preparation

Protocol For:

Automated workflow for preparation of cDNA for cap analysis gene expression on a single molecule sequencer

**Masayoshi Itoh, Miki Kojima, Sayaka Nagao-Sato, Eri Saijo, Timo Lassmann, Mutsumi Kanamori Katayama, Ai Kaiho, Hideya Kawaji, Marina Lizio, Piero Carninci, Alistair R. R. Forrest, Yoshihide Hayashizaki.**

*Omics Science Center, RIKEN Yokohama Institute*

*PLoS ONE DOI:* *10.1371/journal.pone.0030809 (2012)*

**LEGEND**

***!! ATTENTION***

***# HINT***

**** REST***

**REAGENTS**

Random N15 primer (Custom, Operon, Huntsville, AL, USA)

10 mM dNTP (18427-088, Life Technologies, Carlsbad, CA, USA)

Sorbitol (85529-250G, Sigma-Aldrich, St. Louis, MO, USA)

Trehalose (90208, Fluka)

***# Molecular biology grade is available from Life Sciences Advanced Technologies (FL, USA), TDH033***

SuperScriptIII (18080-085, Life Technologies)

Sodium periodate (20504, Thermo Fisher Scientific, Waltham, MA, USA, 311448, Sigma-Aldrich, St. Louis, MO, USA)

Biotin long arm hydrazide (SP1100, Vector Laboratories, Burlingame, CA, USA)

RNase ONE (M4261, Promega, Madison, WI, USA)

tRNA (R1753, Sigma-Aldrich)

MPG streptavidin beads (6124A, Takara Bio, Otsu, Shiga, Japan)

RNase H (2150A, Takara Bio, 18021071, Life Technologies, Carlsbad, CA, USA)

Quant-iT OliGreen ssDNA Reagent and kit (O11492, Life Technologies)

AMPure RNAClean XP beads (A63987, Beckman Coulter, Brea, CA, USA)

AMPure XP beads (A63881, Beckman Coulter)

Chelex 100 resin (143-2832, Bio-Rad, Hercules, CA, USA)

RQ1 RNase-free DNase (M6101, Promega)

Proteinase K (19131, QIAGEN, Hilden, NRW, Germany)

**PROCEDURE**

***!! USE ALL RNase-FREE SOLUTIONS***

***!! USE HIGH RECOVERY TUBES AND TIPS***

***!! USE ICE-W******ATER SLURRY TO MAKE SURE TO CHILL COMPLETELY***

***!! T******OTAL RNA SHOULD HAVE RIN VALUE ≥8 AND CONCENTRATION ≥0.5 µG/µL***

***!! TOTAL RNA ABSORBANCE RATIO OF 260/230 SHOULD BE ≥2***

***# Contamination of chaotropic ion like guanidine inhibits RT reaction. Low 260/230 ratio indicates contamination of guanidine ion.***

1ST STRAND SYNTHESIS

1. Mix 5 µg of total RNA and 1 µl of Random primer N15 and fill up to 6 µl
2. Incubate at 65°C for 5 min
3. Chill on ice for 2 min
4. Prepare RT Enzyme Mix as described in RECIPE and keep on ice-water slurry
5. Add RT Enzyme Mix to RNA/primer mix and pipetting
6. Incubate by the following thermal program
   1. 25°C for 30 sec
   2. 42°C for 30 min
   3. 50°C for 10 min
   4. 56°C for 10 min
   5. 60°C for 10 min
   6. 4°C forever

***# Recommend to program on thermal cycler***

1. Add 1.8 volume of AMPure RNAClean XP slurry and mix by pipetting

***!! PRE-WARM AMPURE RNAClean XP SLURRY BEFORE USE AT ROOM TEMPERATURE TO ENSURE DESPENSING CORRECT VOLUME***

***# The slurry volume should be 68.4 µl***

1. Leave at room temperature for 10 min
2. Mix by pipetting 10 times
3. Repeat 2 times of 10 min leaving and 10 times mixing
4. Centrifuge at 1,000xg, room temp for 5 min
5. Leave on Magnetic stand for 5 min
6. Remove supernatant

***!! ENSURE NOT SUCK ANY MAGNETIC BEADS***

1. Add 200 µl of 70% ethanol

***!! USE FRESHLY PREPARED 70% ETHANOL***

***# No need to suspend magnet beads, but wash tube wall***

1. Remove supernatant
2. Repeat 70% ethanol wash

***!! ENSURE NOT ETHANOL REMAINED***

***!! NEVER DRY UP MAGNET BEADS TO AVOID LOW RECOVERY***

1. Move tube from magnet stand to normal tube stand
2. Add 42 µl of 37°C pre-heated water and mix by pipetting 60 times
3. Leave in 37°C aluminum block for 5 min

***# Thermal cycler is also available***

1. Set on Magnet stand and leave for 5 min
2. Remove supernatant and transfer to new tube

***!! ENSURE NOT SUCK ANY MAGNETIC BEADS***

1. Incubate at 37°C for 10 min

**** RNA/cDNA solution can be stored at -20°C***

OXIDATION OF DIOL IN CAP STRUCTURE

1. Add 2 µl of 1 M NaOAc (pH 4.5)

***!! pH OF NaOAc IS CRITICAL FOR OXIDATION***

1. Add 2 µl of 250 mM NaIO_4_ and mix by pipetting

***!! USE FRESHLY PREPARED SOLUTION BY DISSOLVE 1 MG OF SODIUM PERIODATE IN 18.7 µL WATER***

1. Leave on ice-water slurry in the dark for 45 min
2. Add 2 µl of 40% glycerol and mix well by pipetting
3. Add 14 µl of 1 M Tris-HCl (pH 8.5) and mix by pipetting
4. Add 1.8 volume of AMPure RNAClean XP slurry and mix by pipetting

***!! PRE-WARM AMPURE RNAClean XP SLURRY BEFORE USE AT ROOM TEMPERATURE TO ENSURE DESPENSING CORRECT VOLUME***

***# The slurry volume should be 108 µl***

1. Leave at room temperature for 10 min
2. Mix by pipetting 10 times
3. Repeat 2 times of 10 min leaving and 10 times mixing
4. Centrifude at 1,000xg, room temp for 5 min
5. Leave on Magnetic stand for 5 min
6. Remove supernatant

***!! ENSURE NOT SUCK ANY MAGNETIC BEADS***

1. Add 200 µl of 70% ethanol

***!! USE FRESHLY PREPARED 70% ETHANOL***

***# No need to suspend magnet beads, but wash tube wall***

1. Remove supernatant
2. Repeat 70% ethanol wash

***!! ENSURE NOT ETHANOL REMAINED***

***!! NEVER DRY UP MAGNET BEADS TO AVOID LOW RECOVERY***

1. Move tube from magnet stand to normal tube stand
2. Add 42 µl of 37°C pre-heated water and mix by pipetting 60 times
3. Leave in 37°C aluminum block for 5 min

***# Thermal cycler is also available***

1. Set on Magnet stand and leave for 5 min
2. Remove supernatant and transfer to new tube

***!! ENSURE NOT SUCK ANY MAGNETIC BEADS***

1. Incubate at 37°C for 10 min

**** Oxidized RNA/cDNA solution can be stored at -20°C***

BIOTINYLATION

1. Add 4 µl of 1 M NaOAc (pH 6.0)
2. Add 4 µl of 10 mM Biotin (long arm) hydrazide in DMSO and mix by pipetting
3. Incubate at 23 °C for 2 hr in the dark
4. Add 2.25 volume of AMPure RNAClean XP slurry and mix by pipetting

***!! PRE-WARM AMPURE RNAClean XP SLURRY BEFORE USE AT ROOM TEMPERATURE TO ENSURE DESPENSING CORRECT VOLUME***

***# The slurry volume should be 108 µl***

1. Add 0.25 original volume of isopropanol and mix by pipetting

***# The isopropanol volume should be 12 µl***

1. Leave at room temperature for 10 min
2. Mix by pipetting 10 times
3. Repeat 2 times of 10 min leaving and 10 times mixing
4. Centrifuge at 1,000xg, room temp for 5 min
5. Leave on Magnetic stand for 5 min
6. Remove supernatant

***!! ENSURE NOT SUCK ANY MAGNETIC BEADS***

1. Add 200 µl of 70% ethanol

***!! USE FRESHLY PREPARED 70% ETHANOL***

***# No need to suspend magnet beads, but wash tube wall***

1. Remove supernatant
2. Repeat 70% ethanol wash

***!! ENSURE NOT ETHANOL REMAINED***

***!! NEVER DRY UP MAGNET BEADS TO AVOID LOW RECOVERY***

1. Move tube from magnet stand to normal tube stand
2. Add 42 µl of 37°C pre-heated water and mix by pipetting 60 times
3. Leave in 37°C aluminum block for 5 min

***# Thermal cycler is also available***

1. Set on Magnet stand and leave for 5 min
2. Remove supernatant and transfer to new tube

***!! ENSURE NOT SUCK ANY MAGNETIC BEADS***

1. Incubate at 37°C for 10 min

**** Biotinylated RNA/cDNA solution can be stored at -20°C***

CAP-TRAPPING

1. Prepare RNase I Mix by mixing of 4.5 µl of 10x RNase I buffer and 0.5 µl of 10 unit/µl of RNase I
2. Add 5 µl of RNase I Mix mix and pipetting
3. Incubate at 37°C for 30 min
4. Leave on ice-water slurry till the preparation of streptavidin beads
5. Dispense 50 µl of MPG streptavidin beads slurry
6. Add 0.63 µl of 20 µg/µl tRNA
7. Leave in ice-water slurry with occasional shaking for 30 min
8. Set on magnet stand and leave for 3 min
9. Remove supernatant
10. Add 50 µl of Wash Buffer 1 and mix by tapping
11. Set on magnet stand and leave for 3 min
12. Repeat wash with Wash buffer 1
13. Prepare Wash buffer 1 with tRNA by mixing 105 µl of Wash buffer 1 and 0.63 µl of 20 µg/µl tRNA
14. Resuspend beads in 105 µl of Wash buffer 1 with tRNA

***!! WASHED MPG STREPTAVIDIN BEADS MUST BE USED WITHIN 30 MIN***

***# Beads washing can be done during RNase I digestion***

1. Mix RNase I digested biotinylated RNA/cDNA sample with 105 µl of MPG streptavidin beads slurry
2. Incubate at 37°C for 30 min
3. Set on magnet stand and leave for 3 min
4. Remove supernatant
5. Wash 150 µl of following Wash Buffers by mixing and remove supernatant on magnet stand
   1. Wash buffer 1 1 time
   2. Wash buffer 2 1 time
   3. Wash buffer 3 2 times
   4. Wash buffer 4 2 times
6. Suspend beads in 35 µl of 1x RNase I buffer

***!! NEVER USE 10X RNase I BUFFER***

1. Incubate at 95°C for 5 min and chill on ice-water slurry immediately
2. Set on magnet stand and leave for 3 min
3. Collect supernatant into new tube
4. Resuspend beads in 30 µl of 1x RNase I buffer

***!! NEVER USE 10X RNase I BUFFER***

1. Set on magnet stand and leave for 3 min
2. Collect supernatant
3. Add 3 µl of RNase H and 2 µl of RNase I to recovered supernatant and mix by pipetting
4. Incubate at 37°C for 15 min
5. Add 1.8 volume of AMPure XP slurry

***!! PRE-WARM AMPURE XP SLURRY BEFORE USE AT ROOM TEMPERATURE TO ENSURE DESPENSING CORRECT VOLUME***

***# The slurry volume should be 126 µl***

1. Leave at room temperature for 10 min
2. Mix by pipetting 10 times
3. Repeat 2 times of 10 min leaving and 10 times mixing
4. Centrifuge at 1,000xg, room temp for 5 min
5. Leave on Magnetic stand for 5 min
6. Remove supernatant

***!! ENSURE NOT SUCK ANY MAGNETIC BEADS***

1. Add 200 µl of 70% ethanol

***!! USE FRESHLY PREPARED 70% ETHANOL***

***# No need to suspend magnet beads, but wash tube wall***

1. Remove supernatant
2. Repeat 70% ethanol wash

***!! ENSURE NOT ETHANOL REMAINED***

***!! NEVER DRY UP MAGNET BEADS TO AVOID LOW RECOVERY***

1. Move tube from magnet stand to normal tube stand
2. Add 42 µl of 37°C pre-heated water and mix by pipetting 60 times
3. Leave in 37°C aluminum block for 5 min

***# Thermal cycler is also available***

1. Set on Magnet stand and leave for 5 min
2. Remove supernatant and transfer to new tube

***!! ENSURE NOT SUCK ANY MAGNETIC BEADS***

1. Incubate at 37°C for 10 min

**** Released cDNA solution can be stored at -20°C***

RNASE I DIGESTION

1. Prepare RNase I Mix by mixing 4.5 µl of 10x RNase I buffer and 0.5 µl of 10 unit/µl of RNase I
2. Add 5 µl of RNase I Mix and mix by pipetting
3. Incubate at 37°C for 30 min
4. Add 1.8 volume of AMPure XP slurry

***!! PRE-WARM AMPURE XP SLURRY BEFORE USE AT ROOM TEMPERATURE TO ENSURE DESPENSING CORRECT VOLUME***

***# The slurry volume should be 81 µl***

1. Leave at room temperature for 10 min
2. Mix by pipetting 10 times
3. Repeat 2 times of 10 min leaving and 10 times mixing
4. Centrifuge at 1,000xg, room temp for 5 min
5. Leave on Magnetic stand for 5 min
6. Remove supernatant

***!! ENSURE NOT SUCK ANY MAGNETIC BEADS***

1. Add 200 µl of 70% ethanol

***!! USE FRESHLY PREPARED 70% ETHANOL***

***# No need to suspend magnet beads, but wash tube wall***

1. Remove supernatant
2. Repeat 70% ethanol wash

***!! ENSURE NOT ETHANOL REMAINED***

***!! NEVER DRY UP MAGNET BEADS TO AVOID LOW RECOVERY***

1. Move tube from magnet stand to normal tube stand
2. Add 42 µl of 37°C pre-heated water and mix by pipetting 60 times
3. Leave in 37°C aluminum block for 5 min

***# Thermal cycler is also available***

1. Set on Magnet stand and leave for 5 min
2. Remove supernatant and transfer to new tube

***!! ENSURE NOT SUCK ANY MAGNETIC BEADS***

1. Incubate at 37°C for 10 min

**** Released cDNA solution can be stored at -20°C***

***# Prepared cDNA can be used for quantification and quality check by qPCR***

SAMPLE CONCENTRATION

1. Seal open tube with parafilm and pierce by needle
2. Set in SpeedVac rotor
3. Concentrate less than 12 µl

***!! IF COMPETELY DRIED, DISSOLVE cDNA WITH 12 µL OF WATER BY PIPETTING ON THE TUBE WALL***

1. Use 1 µl for quantification by OliGreen fluorescence assay
2. Use 1 µl for qPCR quality check
3. Use 10 µl for poly-A tailing/blocking

QUANTIFICATION BY OLIGREEN FLUORESCENCE ASSAY

1. Prepare Oligomer working standard by dilution of 4 µl of Oligomer stock for standard with 196 µl of TE
2. Prepare OliGreen working solution by dilution of 5 µl of OliGreen stock solution with 995 µl of TE
3. Make standard curve as follows;
   1. Prepare standard dilution series
      1. Blank: 100 µl of TE
      2. 10 ng/ml: 1 µl of Oligomer working standard + 99 µl of TE
      3. 100 ng/ml: 10 µl of Oligomer working standard + 90 µl of TE
      4. 500 ng/ml: 50 µl of Oligomer working standard + 50 µl of TE
      5. 1000 ng/ml: 100 µl of Oligomer working standard
   2. Add 100 µl of OliGreen working solution
   3. Transfer triplicate of each 9 µl to 384 reader plate
   4. Leave 5 min in the dark
4. Transfer 1 µl of concentrated cDNA to 8-tube strip
5. Add 1 µl of water
6. Add 18 µl of OliGreen working solution
7. Transfer duplicate of each sample to 384 well reader plate
8. Leave 5 min in the dark
9. Read the plate by fluorescence plate reader
10. Estimate sample concentrations using standard curve

***!! MEASURE FLUORESCENCE BY PLATE READER AS SOON AS AFTER LEAVING IN THE DARK***

***# Typical cDNA yield might be 10~20 ng***

**RECIPES**

4.9 M Sorbitol solution (20 ml)

Sorbitol 17.8 g

Dissolve in water and fill up to 20 ml, then autoclave at 121°C for 30 min

Saturated Trehalose solution (10 ml)

Trehalose 7.27 g

Dissolve in water and fill up to 10 ml, then autoclave at 121°C for 30 min

***# Prepare in autoclavable conical 50 ml tube. Add 1-2 ml of water in empty tube before throw trehalose in. Trehalose will dissolve after autoclave even not dissolved just in water.***

Sorbitol/Trehalose (total volume)

Mix Saturated Trehalose solution and 4.9 M Sorbitol solution at 1:2.

***# Mix before cool down.***

Add 1 ml of Chelex 100 resin slurry and vortex, then shake at 45°C for 3-4 hr. The resin was removed by filtration.

***!! STORE AT ROOM TEMPERATURE, NEVER FREEZE***

RT Enzyme Mix (32 µl/sample)

5x SuperScriptIII buffer 7.6 µl 1x final

10 mM dNTP 1 µl 260 µM final

Sorbitol/Trehalose 7.6 µl 0.65 M/0.14 M final

0.1x DTT 1.9 µl 0.005x final

water 3.8 µl

SuperScriptIII (200 unit/µl) 3.8 µl 0.53 unit/µl final

***!! PREPARE FRSHLY***

1 M NaOAc (pH 4.5)

Adjust pH with acetic acid precisely

40% glycerol

w/v in water

1 M Tris-HCl (pH 8.5)

1 M NaOAc (pH 6.0)

Adjust pH with acetic acid precisely

10 mM Biotin long arm hydrazide (270 µl)

Biotin long arm hydrazide 1 mg 10 mM final

DMSO 270 µl

***# Dispense some aliquots for appropriate sample number and store at –80°C***

20 µg/µl tRNA (400 µl)

Add 500 µl of water in tRNA reagent bottle to dissolve tRNA powder by pipetting. The solution was transferred to new siliconized 1.5 ml tube. Add 500 µl of water to empty tRNA reagent bottle to wash the wall, then transferred to former solution tube. Divide the solution into 2 of siliconized 1.5 ml tubes.

Add 45 µl of 10x DNase buffer and 30 µl of DNase in each tube. After incubation at 37°C for 2 hr, Add 10 µl of 0.5 M EDTA (pH 8.0), 10 µl of 10% SDS, and 10 µl of Proteinase K, then incubate at 45°C for 30 min. The solution was extracted with Phenol/Chloroform, Chloroform, and then add 25 µl of 5 M NaCl and 525 µl of isopropanol. The solution was centrifuged at 15,000 rpm, 4°C for 15 min, wash with 80% ethanol twice, then the precipitate was solved in 200 µl of water. The both solution was joined into one tube, quantified by absorbance at 260 nm (such like NanoDrop), then finally adjust the concentration at 20 µg/µl.

Wash buffer 1 (50 ml)

5 M NaCl 45 ml 4.5 M final

0.5 M EDTA (pH 8.0) 5 ml 50 mM final

Wash buffer 2 (50 mL)

5 M NaCl 3 ml 0.3 M final

0.5 M EDTA (pH 8.0) 0.1 ml 1 mM final

fill up to 50 ml with water

Wash buffer 3 (50 ml)

0.5 M EDTA (pH 8.0) 0.1 ml 1 mM final

10% SDS 2 mL 0.4% final

1 M NaOAc (pH 6.0) 25 ml 0.5 M final

1 M Tris-HCl (pH 8.5) 1 ml 20 mM final

fill up to 50 ml with water

Wash buffer 4 (50 ml)

0.5 M EDTA (pH 8.0) 0.1 ml 1 mM final

1 M NaOAc (pH 6.0) 25 ml 0.5 M final

1 M Tris-HCl (pH 8.5) 0.5 ml 10 mM final

fill up to 50 ml with water

70% ethanol

v/v with water

**TROUBLESHOOTING**

LOW RECOVERY

Low quality of total RNA: Purify RNA by some adsorption column

Dry up at AMPure purification: Elute before dry up beads

HIGH RIBOSOMAL READS CONTENT

Excess streptavidin beads: Suspend MPG streptavidin beads slurry completely

**EQUIPMENT**

Tetrad DNA Engine Thermal Cycler PTC-225 (Bio-Rad)

Desktop centrifuge evaporator miVAC DNA (Genevac, Gardiner, NY, USA)

8-tube strip centrifugator WKN-2816 (Wakenyaku, Kyoto, Japan)

Plate centrifugator Allegra 6KR (Beckman Coulter)

96-well ring magnet plate DynaMag-96 Side Skirted (Life Technologies)

Fluorescence plate reader ARVO SX 1420 (Perkin Elmer)
